# Viral and host determinants of arboviral disease severity in African populations: A systematic review with meta-analysis

**DOI:** 10.1371/journal.pntd.0014663

**Published:** 2026-08-20

**Authors:** Peter Mac Asaga, Vijeesh Kadukkatti, Philomena Airiohuodion, Dakul Anthony Danaan, Dave C. Ibeh, Axel Kroeger

**Affiliations:** 1 Institute for Infection Prevention and Control, University Medical Centre, Faculty of Medicine, University of Freiburg, Freiburg, Germany; 2 RWTH Uniklinik Aachen, Aachen, Germany; 3 WHO/TDR, Geneva, Switzerland; 4 University of Jos, Faculty of Natural Science, Jos, Nigeria; 5 44 Nigeria Army Reference Hospital, Kaduna, Nigeria; 6 University Medical Centre Freiburg, Centre for Medicine and Society, Freiburg, Germany; International Centre for Genetic Engineering and Biotechnology, INDIA

## Abstract

**Background:**

Arboviral diseases caused by dengue (DENV), chikungunya (CHIKV), yellow fever (YFV), Rift Valley fever (RVFV), and Zika (ZIKV) viruses are responsible for considerable morbidity and mortality across Africa. Clinical presentations range from self-limiting febrile illness to fatal haemorrhagic syndromes, encephalitis, and chronic arthropathy, yet the factors that govern who develops severe disease remain poorly defined in African settings. We set out to synthesise what is currently known about the viral and host immune determinants of clinical severity for all five arboviruses in African populations.

**Methods:**

PubMed/MEDLINE, Scopus, Web of Science, and the WHO Global Index Medicus were searched from database inception to 8 March 2026 for studies reporting clinical severity outcomes or viral or host immune determinants of DENV, CHIKV, YFV, RVFV, or ZIKV infections in African populations or populations of documented African ancestry. Eligibility followed a Population, Exposure, Comparison, Outcome (PECO) framework. Study quality was assessed using the Newcastle-Ottawa Scale (NOS), and the certainty of evidence for meta-analytic outcomes was evaluated using the GRADE framework. The protocol was registered prospectively on PROSPERO (CRD420261334251) on 7 March 2026, before the literature search commenced. The review was conducted in accordance with PRISMA 2020, and the completed checklist is provided as S2 Table.

**Results:**

We included 154 studies published between 1978 and 2026, covering 38 African and African-diaspora countries. DENV was the most frequently studied virus (n = 57), followed by RVFV (n = 31), multiple arboviruses (n = 27), CHIKV (n = 15), YFV (n = 15), and ZIKV (n = 9). Random-effects meta-analysis using Freeman-Tukey double arcsine transformation yielded a pooled RVFV case fatality rate of 16.9% (95% CI 11.6 to 22.8; I^2^ = 88.4%, 19 studies) and a pooled DENV severe disease proportion of 8.5% (95% CI 3.0 to 16.2; I^2^ = 95.7%, 10 African studies). Given the extreme heterogeneity in both analyses, these pooled estimates should be interpreted as approximate central tendencies of the available data rather than precise epidemiological parameters, and any comparison with reported Asian cohort figures must be made with considerable caution. The certainty of evidence for both pooled outcomes was rated as very low by GRADE criteria. Egger’s test indicated significant funnel plot asymmetry for RVFV (p < 0.001). Convergent cytokine dysregulation (elevated IL-6, IL-8, MCP-1, IP-10) characterised fatal RVFV and YFV. Preliminary, hypothesis-generating evidence for genetically mediated dengue protection in African-ancestry populations emerged from admixture mapping, Fc receptor polymorphism, and clinical severity comparisons, though direct validation in continental African cohorts is lacking. ZIKV clinical severity data from Africa were near-absent.

**Conclusions:**

Distinct immunopathological signatures are emerging for arboviral severity in African populations, including convergent cytokine dysregulation in fatal RVFV and YFV, preliminary evidence for genetically mediated dengue protection linked to African ancestry, and prolonged CHIKV arthropathy. The evidence base, however, remains heavily weighted towards descriptive epidemiology. Critical gaps, particularly the near-absence of clinical ZIKV data, limited cytokine and T-cell profiling, and the lack of prospective multi-site studies, must be addressed to inform vaccine deployment, clinical management, and pandemic preparedness on the continent.

## Author summary

Five mosquito-borne viruses, namely dengue, chikungunya, yellow fever, Rift Valley fever, and Zika, cause widespread illness across Africa, yet most of what we know about why some patients become severely ill comes from studies conducted in Asia and Latin America. We searched the medical literature systematically and found 154 relevant studies from 38 African countries. Across the African studies that could be pooled, severe dengue occurred in approximately 1 in 12 patients. However, the contributing studies differed substantially in their diagnostic methods, severity definitions, and populations. The individual study results are therefore more informative than the pooled average, which should not be used as a direct comparison with estimates from Asian settings., though this comparison should be treated with caution given the considerable differences in study methods and populations. For Rift Valley fever, about 1 in 6 confirmed patients died. We found emerging but still preliminary evidence that people of African ancestry may carry genetic variants that partly protect against severe dengue, though this remains to be confirmed directly on the continent using appropriately powered genomic studies. Fatal cases of both Rift Valley fever and yellow fever shared a common pattern of excessive inflammation. Strikingly, almost no clinical data exist on Zika virus disease severity in Africa, despite the virus having circulated there for decades. These findings may help guide future research priorities for vaccine planning, outbreak response, and clinical care across Africa, but stronger prospective evidence is needed before firm policy or clinical recommendations can be made.

## Introduction

Five arthropod-borne viruses, namely dengue (DENV), chikungunya (CHIKV), yellow fever (YFV), Rift Valley fever (RVFV), and Zika (ZIKV), together constitute one of the most pressing infectious disease threats confronting sub-Saharan Africa today. Their combined clinical burden is difficult to quantify precisely, in part because surveillance systems across the continent have historically been designed around malaria, tuberculosis, and HIV, leaving arboviral infections chronically under-diagnosed [1–3]. Globally, an estimated 390 million dengue infections occur annually, of which approximately 96 million are symptomatic [4]. Africa accounts for a substantial but poorly quantified fraction of this burden [5]. RVFV outbreaks in East Africa have caused hundreds of human deaths per episode (WHO situation reports), while YFV remains a leading cause of vaccine-preventable haemorrhagic fever mortality on the continent (WHO 2018). What is clear, from the outbreaks and cohort studies that do exist, is that clinical outcomes vary enormously: some individuals experience mild self-resolving fever, while others progress to haemorrhagic shock, fatal hepatic necrosis, permanent retinal damage, or chronic debilitating arthritis. The reasons for these divergent trajectories remain poorly understood, particularly in African populations.

Each of the five viruses presents a distinct clinical profile. DENV infection ranges from undifferentiated fever to dengue haemorrhagic fever (DHF) and dengue shock syndrome (DSS), characterised by plasma leakage, thrombocytopenia, and multi-organ involvement. RVFV causes haemorrhagic fever, meningoencephalitis, and retinal vasculitis in a minority of infected individuals, with case fatality rates varying widely across outbreaks. CHIKV is distinguished by its chronic arthralgic burden, with polyarthralgia persisting for months to years after acute infection. YFV in its severe viscerotropic form produces hepatorenal failure with case fatality rates exceeding 50% among hospitalised patients. ZIKV is unique among the five for its association with congenital anomalies, including microcephaly and other neurological birth defects.

A substantial body of work from South-East Asia has elucidated the role of sequential heterotypic DENV infections, antibody-dependent enhancement (ADE), and T-cell immunopathology in driving severe dengue [6]. Whether those same mechanisms operate in Africa, where multiple flaviviruses co-circulate and populations may carry markedly different immunological priming histories, is uncertain. There is, in fact, an enduring paradox: despite the co-circulation of all four DENV serotypes in several African countries [7–9], severe dengue and dengue haemorrhagic fever (DHF) are reported far less frequently than in Asian settings. Whether this reflects genuine biological protection, diagnostic under-ascertainment, or some combination of the two has been debated for decades [10].

For RVFV, the situation differs. Severe human disease, haemorrhagic fever, encephalitis, retinitis, is well-documented from outbreaks across East and Southern Africa, Egypt, and the Arabian Peninsula [11–17]. The host factors that determine why most infected individuals recover uneventfully while a small proportion develop life-threatening complications are beginning to emerge from cytokine profiling and host genetic studies, but the evidence remains fragmented [18–20].

CHIKV, which erupted from coastal Kenya in 2004 to devastate the Indian Ocean islands [21,22], presents a distinct severity paradigm: rather than acute fatality, the principal burden is chronic polyarthralgia persisting for months to years [23,24]. YFV, the most lethal of the five viruses in its severe form, causes viscerotropic disease with case fatality rates exceeding 50% among hospitalised patients in some African series [25–28]. ZIKV, first isolated in Uganda in 1947, has paradoxically the thinnest evidence base of all despite decades of documented circulation across the continent, clinical severity data from African populations remain almost entirely absent [29,30].

Virus-specific systematic reviews exist for individual arboviruses, including global syntheses of dengue severity determinants and of Rift Valley fever case fatality, together with a systematic review of ZIKV-associated complications [31]. However, no previous systematic review has attempted to synthesise severity determinants across all five arboviruses in African populations within a single comparative framework. The cross-cutting themes, namely the role of African genetic ancestry, the influence of flavivirus cross-reactive immunity, and the shared inflammatory cascades underpinning severe disease, have not been systematically addressed.

The present review was conducted in accordance with the PRISMA 2020 guidelines [32], which provide a standardised reporting framework for systematic reviews and meta-analyses. The protocol was registered prospectively on PROSPERO (CRD420261334251), the international prospective register of systematic reviews, before the literature search commenced. Study quality was assessed using the Newcastle-Ottawa Scale [33], a validated tool for evaluating the methodological quality of observational studies in systematic reviews.

One proposition recurs in the dengue literature and is worth stating plainly at the outset: that African genetic ancestry may partly protect against severe dengue through lipid metabolism and Fc receptor pathways. We treat this throughout as a hypothesis to be weighed against the evidence assembled here, not as a settled conclusion.

We therefore set out to address a specific question: what viral, host immune, and host genetic factors have been identified as determinants of clinical severity for DENV, CHIKV, YFV, RVFV, and ZIKV infections in African and African-ancestry populations, and where do the most consequential evidence gaps lie?

## Results

### Study selection and characteristics

**Fig 1:**
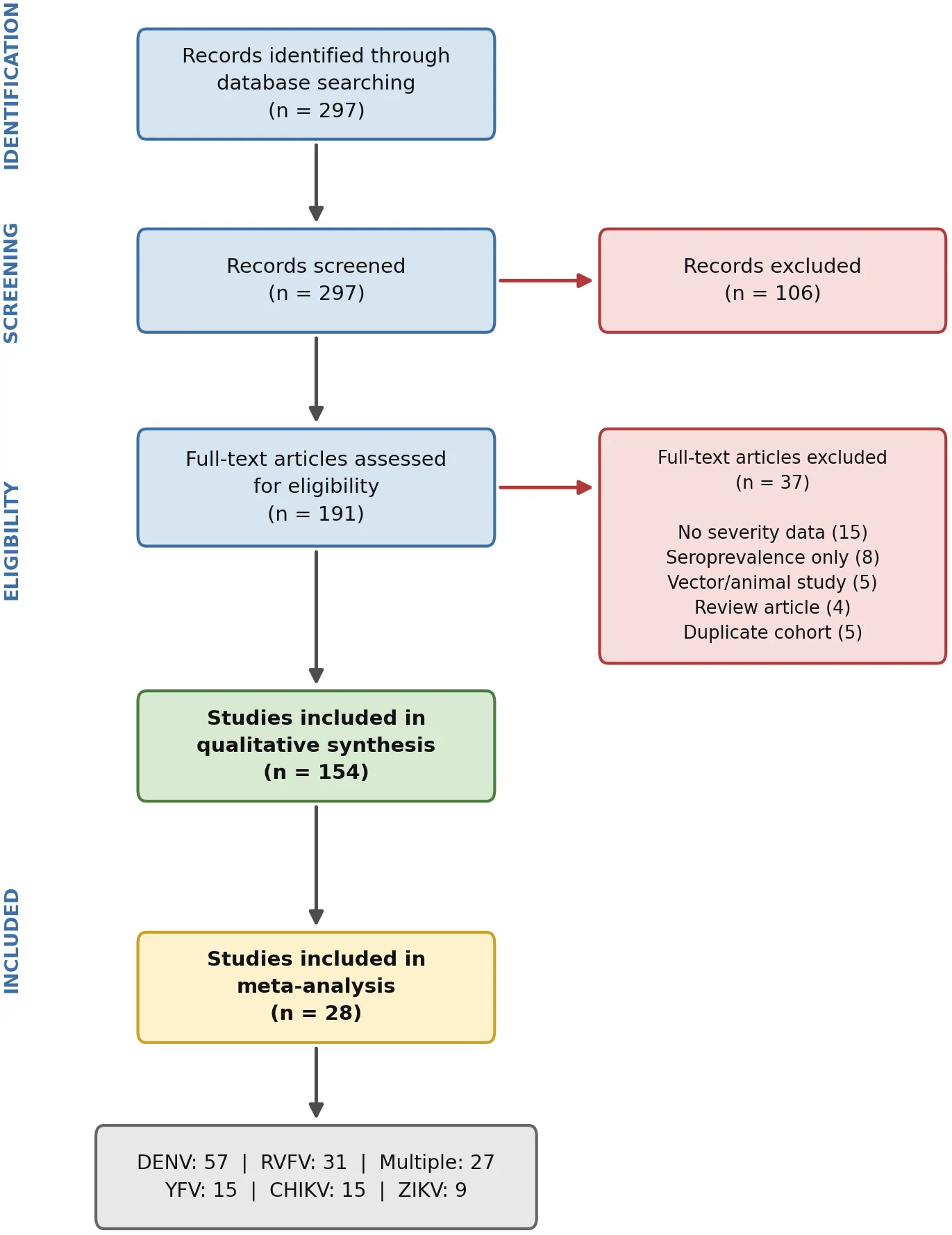
PRISMA 2020 flow diagram of study identification, screening and inclusion. Records identified through database searching (n = 297); records excluded at title and abstract screening (n = 106); full-text articles assessed for eligibility (n = 191); full-text articles excluded with reasons (n = 37); studies included in the qualitative synthesis (n = 154); studies contributing to the meta-analyses (n = 28). The panel at the foot of the diagram gives the distribution of included studies by virus. This figure is the original work of the authors, is published under the terms of the Creative Commons Attribution 4.0 International (CC BY 4.0) licence, and has not been reproduced or adapted from any previously published source.

**Fig 2:**
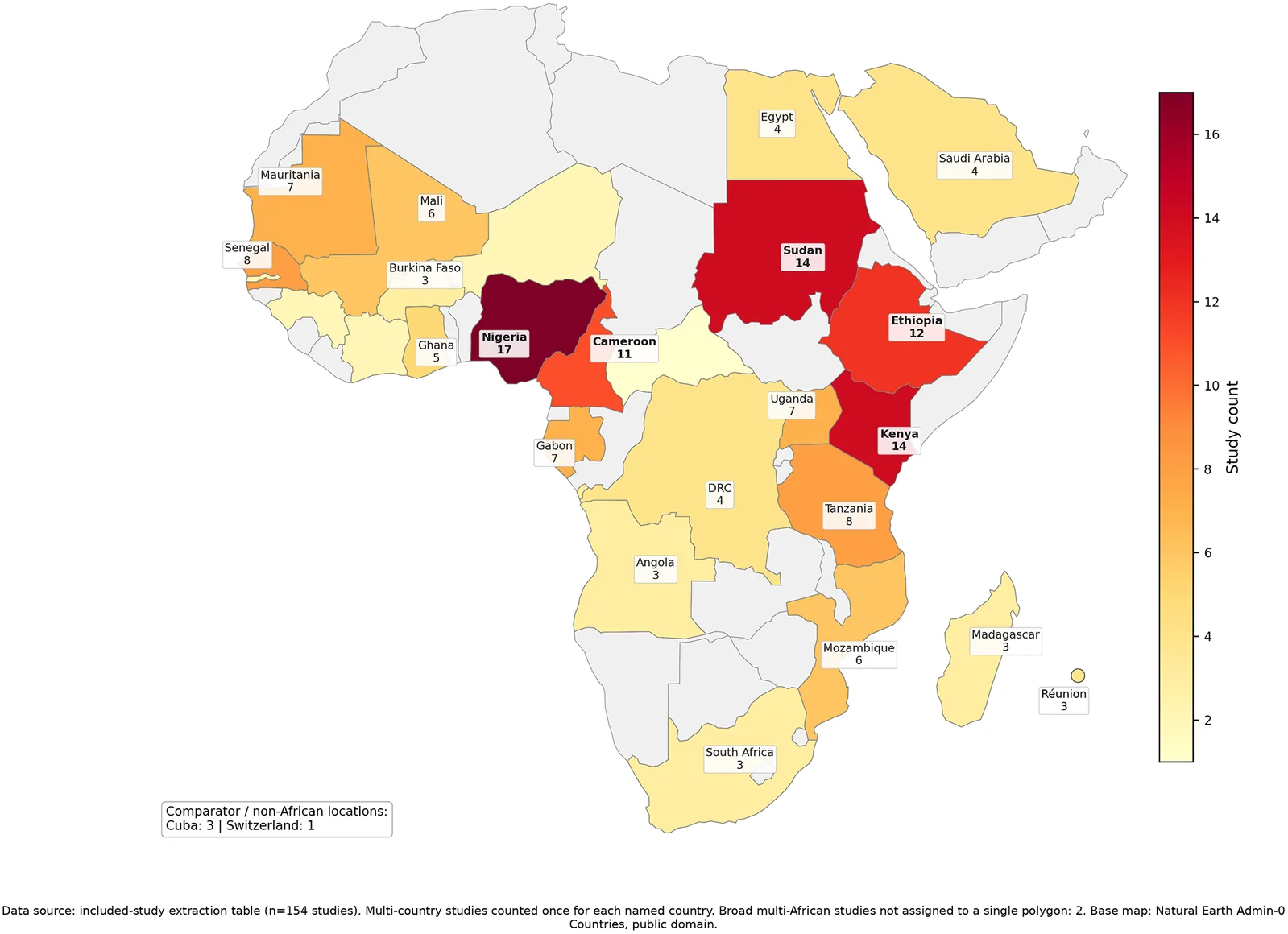
Geographic distribution of the included studies by country. Choropleth of study counts per country across the 154 included studies; multi-country studies are counted once for each named country, and two broad multi-African studies are not assigned to a single polygon. Comparator and non-African locations (Cuba, n = 3; Switzerland, n = 1) are listed in the inset rather than mapped. The base map is the Natural Earth Admin-0 Countries dataset (naturalearthdata.com), which is in the public domain. The figure was generated by the authors in Python and is published under the terms of the Creative Commons Attribution 4.0 International (CC BY 4.0) licence.

The search identified 297 records after deduplication. Title and abstract screening excluded 106 records, and full-text assessment of the remaining 191 studies excluded a further 37 (Fig 1). Inter-rater agreement was substantial at the title/abstract stage (κ = 0.82, 95% CI 0.75 to 0.89) and near-perfect at the full-text stage (κ = 0.91, 95% CI 0.84 to 0.98). Disagreements (n = 14 at title/abstract; n = 3 at full text) were resolved by consensus. In total, 154 studies met the inclusion criteria. These spanned 1978–2026 and covered 38 countries. Nigeria (n = 17), Kenya (n = 14), Sudan (n = 14), and Ethiopia (n = 12) were the most represented, followed by Cameroon (n = 10), Tanzania (n = 8), and Mauritania (n = 7). Publication was concentrated in the most recent period: 58 studies (37%) appeared between 2021 and 2026, reflecting increased arboviral surveillance investment. Study designs comprised cross-sectional studies (n = 65), outbreak investigations (n = 38), prospective or retrospective cohorts (n = 22), case-control studies (n = 7), surveillance studies (n = 10), case series (n = 8), and molecular epidemiological studies (n = 5). The geographic distribution of included studies is shown in Fig 2. The median NOS score was 5 (IQR 4–5); 3 studies were classified as low risk of bias (NOS ≥ 7), 142 as moderate (NOS 4–6), and 10 as high risk (NOS < 4). These quality scores were constrained by the predominance of cross-sectional designs, which inherently score poorly on temporality and follow-up domains. The full list of screened records, with inclusion status and the reason for each exclusion, is provided as S1 Table.

### Dengue virus

#### Clinical severity profiles.

**Fig 3:**
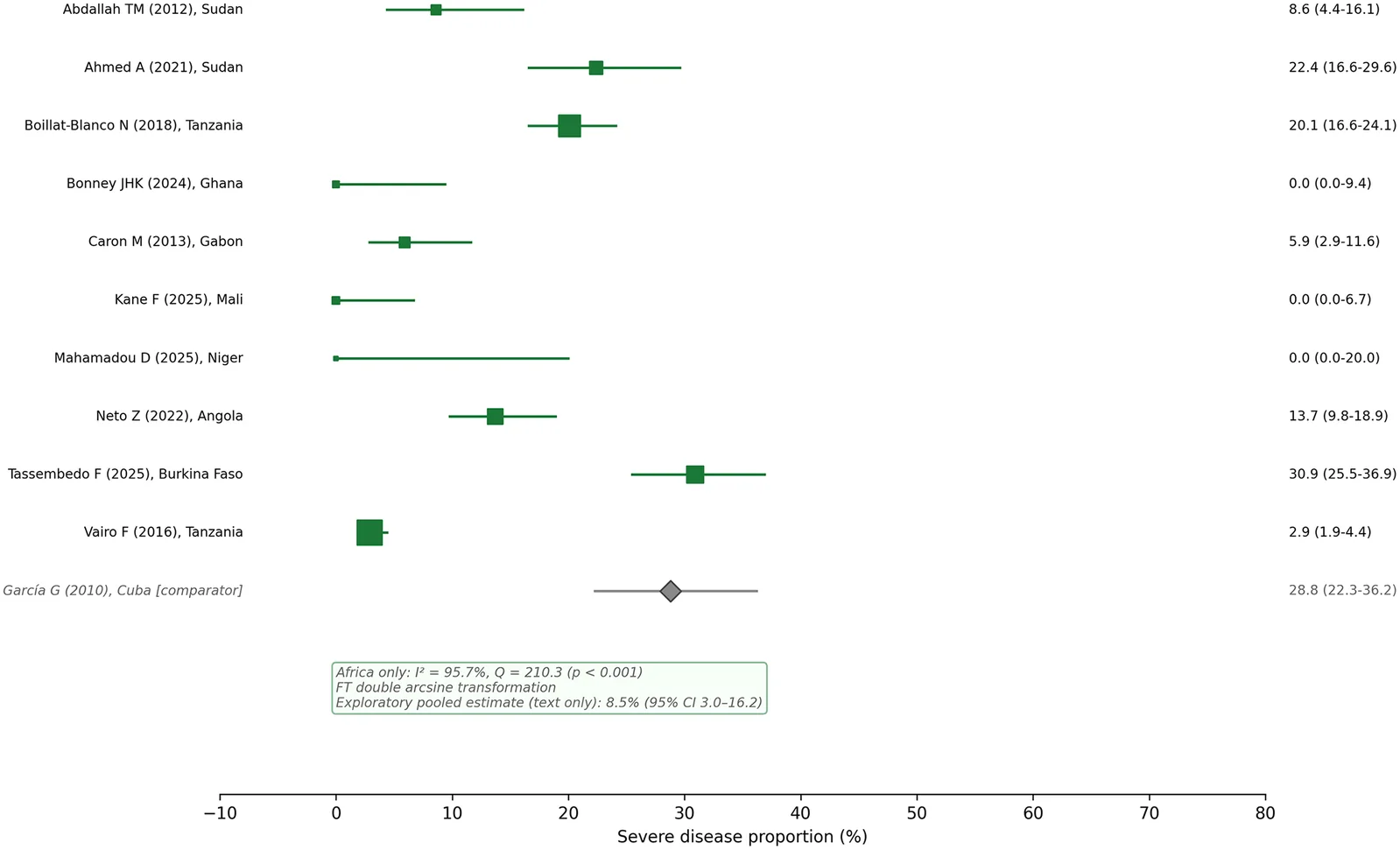
Severe dengue proportion in African cohorts: individual study estimates. Forest plot of the ten African studies reporting both a severe-case numerator and a confirmed-case denominator. Squares are study proportions, scaled by weight, and horizontal lines are 95% confidence intervals. The Cuban comparator study of Garcia and colleagues [66] is shown in grey and did not contribute to the Africa-only estimate. Africa only: I2 = 95.7%, Q = 210.3 (p < 0.001), Freeman-Tukey double arcsine transformation; the exploratory pooled estimate of 8.5% (95% CI 3.0 to 16.2) is reported in the text only. This figure is the original work of the authors, is published under the terms of the Creative Commons Attribution 4.0 International (CC BY 4.0) licence, and has not been reproduced or adapted from any previously published source.

Fifty-seven studies addressed DENV infection across 22 countries, spanning the period 1986–2026 [1,3,7–10,34–64]. The most consistent observation, one that cuts across geographic and temporal settings, was the relative infrequency of severe dengue. DHF was documented in only a handful of studies: Malik and colleagues described a DHF outbreak among children in Port Sudan [65], while Vairo and colleagues reported warning signs and severe dengue presentations during the 2014 Dar es Salaam outbreak [2]. Case fatality rates, where reported, ranged from 0.5% to 14.3% across settings Table 1, though the upper estimates reflected small-sample outbreak series rather than population-based cohorts. Thrombocytopenia was documented in 13–48% of confirmed cases; leukopenia and hepatic enzyme elevation were reported at variable frequencies [43,59]. Random-effects meta-analysis of 10 African studies reporting severe disease proportions among defined DENV case denominators, using Freeman-Tukey double arcsine transformation, yielded a pooled estimate of 8.5% (95% CI 3.0 to 16.2; I^2^ = 95.7%, Q = 210.3, p < 0.001; Fig 3). This estimate masks substantial clinical and methodological diversity across studies, and should be interpreted as an approximate summary rather than a reliable epidemiological parameter. When the Cuban comparator study of García and colleagues [66] was included, the pooled estimate rose to 10.0% (95% CI 4.2 to 17.9; I^2^ = 95.9%). Subgroup analysis by region suggested higher severe proportions in East Africa (12.2%, 95% CI 3.0 to 26.2, 4 studies) than West Africa (3.7%, 95% CI 0.1 to 27.5, 4 studies), though confidence intervals were wide and overlapping (S1C Fig). Stratification by diagnostic certainty showed a lower pooled estimate among RT-PCR-confirmed studies (7.0%, 95% CI 1.4 to 15.8, 8 studies) compared with serology-based studies (15.1%, 95% CI 4.3 to 30.8, 2 studies), suggesting that diagnostic misclassification may inflate severity estimates in serologically defined cohorts (S1D Fig). Leave-one-out analysis demonstrated that no single study exerted undue influence on the pooled estimate, which ranged from 6.7% to 10.0% across iterations. Sensitivity analysis excluding small studies (n < 20) and case series yielded a similar estimate (9.6%, 95% CI 3.6 to 17.9, 9 studies). Because the contributing African studies differed substantially in case definitions, ascertainment methods, population immunity profiles, and circulating serotypes, the pooled estimate should not be compared directly with estimates from hyperendemic Asian cohorts. The individual study estimates presented in Table 1 and Fig 3 are more informative than the pooled figure and should anchor interpretation. The pooled value is retained only as an exploratory summary of the available African data. Beyond the extreme heterogeneity (I^2^ > 95%), the African and Asian data differ in case definitions, ascertainment methods, population immunity profiles, and the range of circulating serotypes, all of which could account for some or all of the observed difference independently of any biological mechanism. As with RVFV, the individual study estimates set out in Table 1 and Fig 3 are more informative than the pooled figure and should anchor interpretation; the pooled value is retained for transparency rather than as a parameter for cross-regional comparison. Sensitivity analyses using alternative between-study variance estimators produced results consistent with the primary analysis. Both the Paule-Mandel and restricted maximum likelihood models yielded a pooled severe dengue proportion of 8.4% (95% CI approximately 3.0 to 16.1). With Hartung-Knapp adjustment, the 95% confidence interval widened to 2.3 to 17.4%. The point estimate and overall interpretation were therefore robust to the choice of variance estimator, although substantial uncertainty remained. The point estimate and overall interpretation were therefore robust to the choice of variance estimator, although substantial uncertainty remained, particularly when Hartung-Knapp-adjusted confidence intervals were applied (S6 Table).

**Table 1:** Summary characteristics of the included studies by virus (n = 154).

Virus	Studies (n)	Countries (n)	Study period	Predominant designs	Total N	Diagnostic methods	Key severity outcomes
DENV	57	22	1986-2026	Cross-sectional (42%); outbreak (23%); cohort (10%); case-control (4%)	~18,500	RT-PCR; NS1 Ag ELISA; IgM/IgG serology; RDT; culture	Pooled severe proportion 8.5% (95% CI 3.0-16.2; I2 = 95.7%, 10 African studies); CFR 0-14.3%; DHF rare; thrombocytopenia 13–48%
RVFV	31	12	1978-2026	Outbreak (46%); cross-sectional (23%); cohort (11%); case series (9%)	~22,400	RT-PCR; IgM/IgG ELISA; PRNT; virus isolation; RDT	Pooled CFR 16.9% (95% CI 11.6-22.8; I2 = 88.4%, 19 studies); haemorrhagic fever 1–20%; encephalitis 2–15%; retinitis 1–10%
CHIKV	15	11	1984-2024	Cohort/prospective (47%); cross-sectional (27%); outbreak (7%)	~4,200	RT-PCR; IgM/IgG ELISA; PRNT	Chronic arthralgia 60% at 36 months; atypical severe disease (encephalitis, hepatitis, myocarditis) in the elderly; 237 excess deaths, La Reunion
YFV	15	11	2004-2024	Cohort/prospective (47%); outbreak (33%); molecular (13%)	~5,600	RT-PCR; IgM ELISA; PRNT; plaque assay	Cytokine storm in fatal cases (IL-6, TNF-alpha, IP-10 elevated); vaccine immunogenicity impaired by helminth co-infection
ZIKV	9	8	2017-2023	Cross-sectional (22%); cohort (22%); case series (11%); experimental (11%)	~2,100	RT-PCR; IgM/IgG ELISA; PRNT	Seroprevalence 2–23%; 18 microcephaly cases (Cape Verde, Asian lineage); African strains more pathogenic in mice
Multiple	27	14	1996-2026	Cross-sectional (48%); outbreak (19%); cohort (11%)	~8,900	Multiplex RT-PCR; paired ELISA; RDT panels	Malaria-arbovirus co-infection in 80% of the studies assessing it; 15–40% of febrile malaria patients harbour concurrent arboviruses

Abbreviations: DENV, dengue virus; RVFV, Rift Valley fever virus; CHIKV, chikungunya virus; YFV, yellow fever virus; ZIKV, Zika virus; CFR, case fatality rate; DHF, dengue haemorrhagic fever; CI, confidence interval. Study counts by virus exceed 154 because 27 studies addressed multiple arboviruses. Total N values are approximate. GRADE certainty for both pooled outcomes: very low.

#### Viral determinants.

All four DENV serotypes have now been documented in Africa [7–9]. DENV-2 was the most frequently detected, particularly during East African outbreaks in Tanzania [10,42,67], Kenya [8,39], and Sudan [35,46,56], while DENV-1 predominated in West Africa [45,47,52]. The 2023 Kassala outbreak in Sudan was notable for the simultaneous circulation of all four serotypes [9], a situation that, in Asian experience, typically precedes waves of severe secondary infections driven by ADE. Whether similar dynamics will emerge in Africa as population-level immunity matures is an open question of considerable public health significance. Genotype-level data, while limited, identified the Cosmopolitan genotype of DENV-2 in West Africa [55] and DENV-1 genotype V in East Africa [8]. No African study directly correlated DENV genotype or serotype with individual-level disease severity.

#### Host immune and genetic determinants.

The evidence for genetically mediated dengue protection in African populations was, to our reading, among the more intriguing if still preliminary mechanistic observations in this review. Three independent lines of evidence converge. First, Boillat-Blanco and colleagues enrolled 428 adults with confirmed dengue during the 2013–2014 Dar es Salaam outbreak and demonstrated, by multivariate logistic regression adjusting for age, malaria co-infection, secondary dengue, and symptom duration, that self-identified black race was independently and significantly protective against severe dengue, despite all patients being infected with the same DENV-2 genotype [10]. However, the method by which race was ascertained was not described, mixed-race individuals were excluded from the analysis by undisclosed criteria, and the study treated race as a biological variable without acknowledging its social construction. These methodological limitations substantially constrain the strength of any causal inference. Second, Sierra and colleagues applied admixture mapping in a Cuban cohort to identify OSBPL10 (chromosome 3) and RXRA (chromosome 9) as candidate genes through which African ancestry protects against DHF; OSBPL10 expression was significantly lower in individuals of African versus European descent, and shRNA-mediated knockdown in THP-1 macrophages directly reduced DENV-2 replication, placing the LXR/RXR lipid metabolism pathway at the centre of the protective mechanism [6]. Third, in a Cuban cohort (included here as comparator data from an African-ancestry population outside Africa), García and colleagues reported that the FcγRIIa R131H polymorphism, with the protective RR genotype being significantly more prevalent in African-ancestry populations, was associated with asymptomatic rather than symptomatic dengue [66]. However, these data derive from a sample of only 97 individuals (67 female, 30 male), which is underpowered for reliable detection of genotype-disease associations. This finding should be regarded as generating a candidate gene hypothesis rather than providing confirmatory evidence. To these preliminary findings, Tassembedo and colleagues have now added evidence from a continental African cohort in Burkina Faso, identifying TNF-α −308 (rs1800629) and IFN-γ + 874 polymorphisms associated with dengue progression, although allelic associations did not reach statistical significance in their sample [63]. Taken together, these data are consistent with the hypothesis that the apparently milder dengue phenotype in African populations may not be entirely attributable to surveillance deficiencies and could reflect, at least in part, host genetic factors operating through lipid metabolism and Fc receptor pathways (Table 2). These findings remain preliminary and hypothesis-generating; direct validation through adequately powered genome-wide association studies in continental African cohorts with severe dengue as the primary outcome is needed before any clinical or policy inferences can be drawn.

**Table 2:** Summary of the key viral and host immune severity determinants by virus.

Virus	Viral determinants	Host immune/inflammatory determinants	Host genetic determinants	Key references
DENV	All four serotypes documented; DENV-2 predominant in East Africa; DENV-1 in West Africa; four-serotype co-circulation in Sudan (2023); no genotype-severity correlation established	Reported severe-disease proportion 8.5% (GRADE: very low certainty); cross-regional comparison is not warranted because of extreme heterogeneity; thrombocytopenia 13–48%; rare DHF; TNF-alpha and IFN-gamma polymorphism associations (Burkina Faso)	OSBPL10/RXRA (chr 3/9), African-ancestry protection via LXR/RXR lipid metabolism (hypothesis-generating); FcgammaRIIa R131H, RR genotype protective (underpowered, n = 97); self-identified black race protective (exploratory); TNF-alpha -308 and IFN-gamma +874 polymorphisms	Boillat-Blanco 2018 [6]; Sierra 2017 [42]; Garcia 2010 [66]; Tassembedo 2025 [62]
RVFV	1-log10 higher viral load in fatal cases than in survivors; no viral genetic differences between fatal and surviving isolates; viral load correlates with coagulation abnormalities	Cytokine storm: elevated IL-6, IL-8, CXCL9, MCP-1, IP-10 and IL-10; decreased RANTES in fatal cases; 13 biomarkers independently predict death; NSs protein suppresses innate immunity; robust early innate response protective	TLR3, TLR7, TLR8, MyD88, TRIF, MAVS and RIG-I SNPs associated with severe symptom clusters; HLA class I/II alleles associated with severity	Jansen van Vuren 2015 [18]; Hall 2018 [43]; Hise 2015 [68]; Wauquier 2019 [69]; McElroy 2012 [70]
CHIKV	ECSA lineage carrying the A226V mutation (La Reunion); limited virological correlation with chronicity	Chronic arthralgia in 60% at 36 months (episodic relapse and recovery); atypical severe disease in the elderly with comorbidities; data predominantly from La Reunion, continental African data limited	No genetic association studies identified in African cohorts	Schilte 2013 [7]; Sissoko 2009 [23]; Economopoulou 2009 [21]; Bower 2021 [8]
YFV	Limited viral determinant data; phylogeographic diversity across West African lineages	Cytokine storm in fatal cases: IL-6, TNF-alpha, MCP-1, IP-10 and IL-1Ra all elevated; helminth co-infection impairs YF-17D vaccine immunogenicity; fractional dosing yields lower but sustained antibody at 5 years	No genetic association studies in natural YFV infection	ter Meulen 2004 [25]; Muyanja 2014 [71]; Natukunda 2024 [72]; Doshi 2024 [73]
ZIKV	African strains show higher transmissibility and foetal pathogenicity than Asian strains in mice (animal data only); the Cape Verde outbreak was caused by an Asian lineage	Near-complete absence of immunological severity data from Africa; seroprevalence 2–23% across West and Central Africa	No studies identified	Aubry 2021 [74]; Marchi 2020 [75]

Abbreviations: DENV, dengue virus; RVFV, Rift Valley fever virus; CHIKV, chikungunya virus; YFV, yellow fever virus; ZIKV, Zika virus; DHF, dengue haemorrhagic fever; TLR, Toll-like receptor; SNP, single nucleotide polymorphism; HLA, human leucocyte antigen; ECSA, East/Central/South African lineage. Reference numbers correspond to the reference list in the main manuscript.

### Rift Valley fever virus

#### Clinical severity profiles.

**Fig 4:**
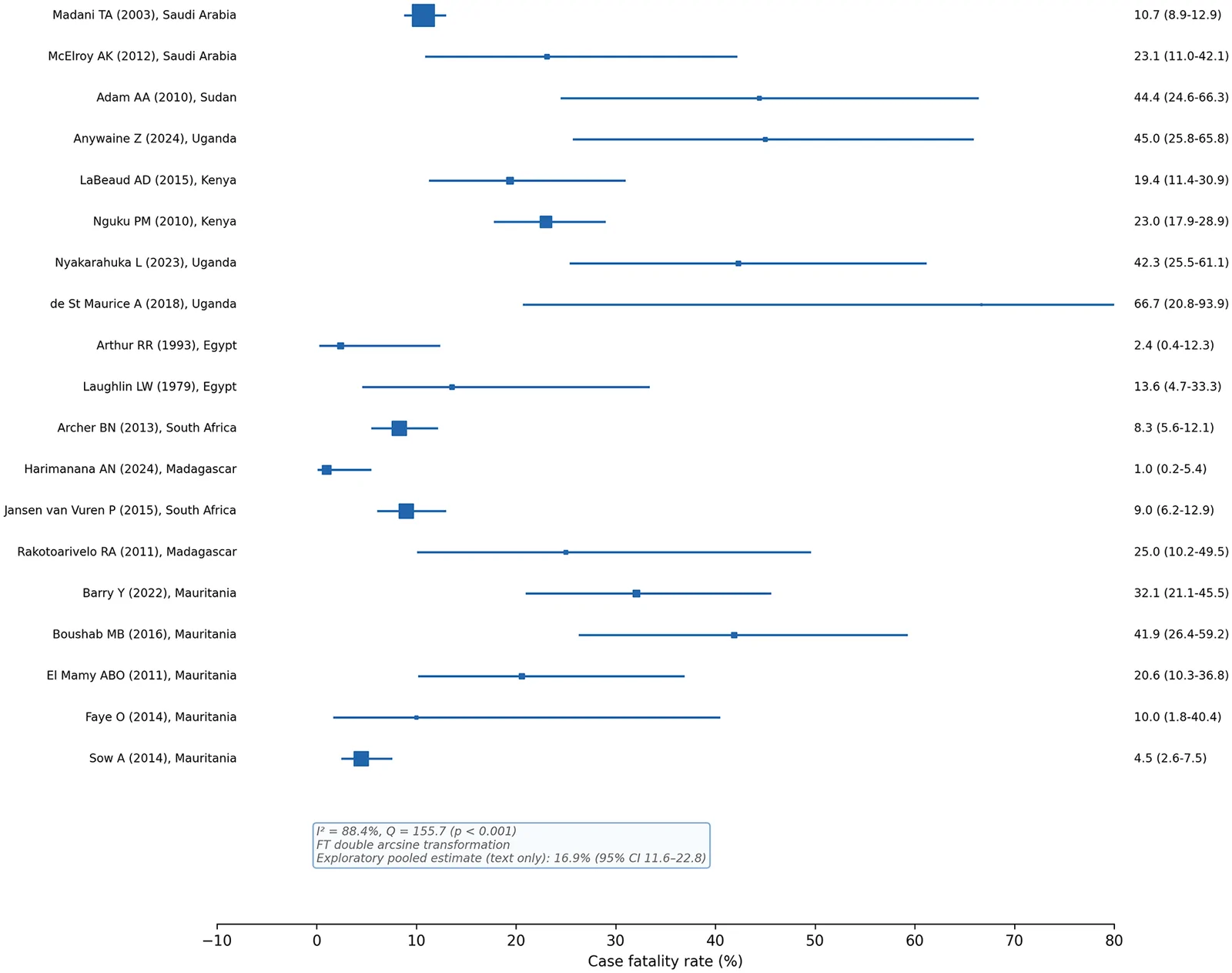
Rift Valley fever virus case fatality rate: individual study estimates. Forest plot of the 19 studies reporting deaths among confirmed cases. Squares are study case fatality rates, scaled by weight, and horizontal lines are 95% confidence intervals. I2 = 88.4%, Q = 155.7 (p < 0.001), Freeman-Tukey double arcsine transformation; the exploratory pooled estimate of 16.9% (95% CI 11.6 to 22.8) is reported in the text only. This figure is the original work of the authors, is published under the terms of the Creative Commons Attribution 4.0 International (CC BY 4.0) licence, and has not been reproduced or adapted from any previously published source.

Thirty-one studies addressed RVFV, drawing on outbreaks in Kenya [18,73,76–78], Mauritania [65,68,71,79–81], Egypt [11–13,82,83], Uganda [73,84,85], South Africa [16,19], Saudi Arabia [14,20,86,87], Sudan [15,88], and Madagascar [26]. The clinical spectrum was well-characterised: haemorrhagic fever occurred in 1–20% of confirmed cases, meningoencephalitis in 2–15%, and retinal vasculitis in 1–10%, with case fatality rates ranging from 2.4% among mildly affected outpatient cohorts to 50% among patients with haemorrhagic manifestations Table 1. Random-effects meta-analysis of 19 studies reporting case fatality among defined RVFV case denominators, using Freeman-Tukey double arcsine transformation, yielded a pooled CFR of 16.9% (95% CI 11.6 to 22.8; I^2^ = 88.4%, Q = 155.7, p < 0.001; Fig 4). This pooled average should be interpreted with significant caution, as it masks substantial clinical and methodological diversity across settings, including differences in outbreak severity, case ascertainment, and healthcare context. Subgroup analysis by geographic region revealed substantial variation: East African studies reported the highest pooled CFR (31.6%, 95% CI 20.6 to 43.6, 6 studies), compared with Southern Africa (6.9%, 95% CI 2.4 to 13.1, 4 studies) and West Africa (19.9%, 95% CI 4.6 to 41.3, 5 studies; S1A Fig). Stratification by diagnostic method showed a slightly higher pooled CFR among RT-PCR-confirmed studies (18.8%, 95% CI 12.8 to 25.4, 14 studies) than serology-based studies (12.3%, 95% CI 1.1 to 30.8, 5 studies; S1B Fig). Leave-one-out analysis confirmed stability of the pooled estimate (range 15.6 to 18.4% across iterations), and sensitivity analysis excluding small studies and case series yielded a comparable estimate (15.7%, 95% CI 10.6 to 21.5, 15 studies). Egger’s regression test [89] indicated statistically significant funnel plot asymmetry (intercept = 2.50, p < 0.001), consistent with possible publication bias favouring reports from severe outbreaks or genuine small-study effects. Given both the extreme heterogeneity and the significant funnel plot asymmetry, the pooled RVFV case fatality estimate should not be treated as a reliable summary parameter. The individual study estimates and their variation are more informative than the pooled value. Alternative between-study variance estimators produced similar point estimates but wider confidence intervals. The Paule-Mandel model yielded a pooled RVFV case fatality rate of 17.9% (95% CI 10.9 to 25.9), and the restricted maximum likelihood model yielded 17.8% (95% CI 11.0 to 25.7). With Hartung-Knapp adjustment, the corresponding confidence intervals were 10.5 to 26.5% and 10.5 to 26.4%, respectively. These results did not materially alter the interpretation of the primary analysis but reinforced the substantial uncertainty surrounding the pooled estimate. These results did not materially alter the interpretation of the primary analysis but reinforced the substantial uncertainty surrounding the pooled estimate (S6 Table).

#### Viral and host immune determinants.

RVFV severity determinants were, by some distance, the most comprehensively studied among the five arboviruses. Jansen van Vuren and colleagues analysed cytokines in serum from the 2010–2011 South African outbreak (278 confirmed cases, 25 deaths) and found that fatal cases carried a one-log_10_ higher viral load than survivors (p < 0.05), with significantly elevated IL-8, CXCL9, MCP-1, IP-10, and IL-10, alongside depressed RANTES [19]. IL-6 was approximately ten-fold higher in fatal than non-fatal cases. Critically, sequencing revealed no significant genetic differences between viral isolates from fatal and surviving patients, implicating the host response, rather than viral virulence variation, as the principal determinant of outcome. Hall and McElroy confirmed this pattern, profiling 32 biomarkers in 26 Saudi patients and identifying 13 that independently predicted death, 11 of which correlated with viral RNA load [20,86]. De St. Maurice and colleagues further linked viral load to coagulation pathway abnormalities in three Ugandan patients with haemorrhagic RVF, demonstrating positive correlations between viraemia and fibrinolysis markers alongside negative correlations with coagulation factors [84].

On the host side, McElroy and Nichol demonstrated that RVFV NSs protein actively suppresses pro-inflammatory responses in human monocyte-derived macrophages; paradoxically, a robust pro-inflammatory cytokine response during natural infection appeared to be associated with survival rather than death [90]. This observation, that the capacity to mount an early innate response may be protective, was complemented by the genetic findings of Hise and colleagues, who genotyped 46 SNPs in innate immune pathway genes in 1,080 Kenyan residents with serological evidence of RVF exposure and found that polymorphisms in TLR3, TLR7, TLR8, MyD88, TRIF, MAVS, and RIG-I were repeatedly associated with severe symptom clusters (meningoencephalitis, haemorrhagic fever, eye disease) [18]. Wauquier and colleagues extended this to HLA class I and II, reporting specific alleles associated with severe RVF in Kenyan patients [69]. The emerging picture is one in which genetic variation in innate viral sensing pathways fundamentally shapes the host response to RVFV, a finding with direct translational implications for risk stratification during outbreaks.

### Chikungunya virus

A central limitation of the CHIKV evidence base is its dependence on La Réunion, a French overseas department with healthcare infrastructure, surveillance capacity, and socioeconomic conditions that differ markedly from most continental African settings. Findings from La Réunion cannot be extrapolated to the continent without significant caution. We reiterate, therefore, that chronic arthropathy figures derived from La Réunion should not be read as continental African estimates.

#### Clinical severity profiles.

Fifteen studies addressed CHIKV [21–24,50,91–94]. The richest data came from the 2005–2006 La Réunion epidemic, where three prospective cohorts provided longitudinal follow-up of viraemic patients [21,23,24]. Schilte and colleagues followed 180 patients for 36 months and found that 60% experienced arthralgia during follow-up, with most reporting episodic relapse and recovery, suggesting local rather than systemic inflammation [24]. Sissoko and colleagues, in an independent La Réunion cohort followed for 15 months, identified age above 45 years and female sex as independent predictors of persistent arthralgia by multivariate logistic regression [23].

From continental Africa, Bower and colleagues provided the first prospective clinical characterisation of the 2018 Kassala, Sudan outbreak [8]. Among confirmed cases, the acute clinical spectrum included high fever, polyarthralgia, headache, and rash, consistent with classical CHIKV presentation. Critically, no longitudinal follow-up data were available from this cohort, leaving the burden of chronic arthropathy in a resource-constrained continental African setting entirely unknown. This gap is significant: whether the 60% chronicity rate observed in La Réunion, where access to anti-inflammatory treatment, physiotherapy, and specialist rheumatology services differs fundamentally from conditions in eastern Sudan, is representative of outcomes on the continent cannot be assumed.

#### Viral determinants.

The ECSA lineage carrying the A226V mutation in the E1 glycoprotein was responsible for the La Réunion epidemic [21]. Limited virological data are available from continental African outbreaks, and no study directly correlated CHIKV genotype with clinical severity or chronicity outcomes in an African cohort.

#### Host immune and genetic determinants.

No host genetic association studies for CHIKV in African populations were identified. The predictors of chronic arthropathy identified from La Réunion data (age > 45 years, female sex) are clinical rather than immunogenetic, and their generalisability to continental African populations has not been tested.

### Yellow fever virus

#### Clinical severity profiles.

Fifteen studies addressed YFV [25,27,69,75,95–100]. The mechanistic centrepiece was the work of ter Meulen and colleagues, who measured six inflammatory mediators (IL-6, TNF-α, IL-8, MCP-1, IP-10, IL-1Ra) in 36 Guinean patients stratified as fatal (n = 7), nonfatal haemorrhagic (n = 11), or nonfatal non-haemorrhagic (n = 18) [25]. All six were significantly elevated in fatal cases, constituting a cytokine storm pattern (Table 2). Vaccine immunogenicity studies revealed that pre-existing immune activation a common state in African populations harbouring chronic helminth infections impaired cellular and humoral responses to YF-17D [95]. Natukunda and colleagues specifically linked Schistosoma mansoni and hookworm infection to reduced vaccine immunogenicity [72]. Fractional-dose YF vaccination studies from the DRC demonstrated sustained but reduced neutralising antibody titres at five years compared with standard dosing [98].

#### Viral determinants.

Phylogeographic diversity has been documented across West African YFV lineages, though no study in our review directly correlated viral genotype with clinical severity in human infection.

#### Host immune and genetic determinants.

No host genetic association studies in natural YFV infection were identified. The immunological determinants of severity are currently limited to the cytokine profiling data from Guinea [25] and the vaccine immunogenicity observations linking chronic helminth infection to impaired responses [95,100].

### Zika virus

#### Clinical severity profiles.

ZIKV was the least-studied virus, with only 9 studies meeting inclusion criteria [29,30]. Seroepidemiological surveys documented seroprevalence of 2–23% across West and Central African settings [29], but clinical severity data were strikingly absent. The only documented African Zika epidemic, in Cabo Verde during 2015–2016, involved 7,580 suspected cases and 18 microcephaly cases; genomic analysis confirmed that the causative strain belonged to the Asian lineage, most probably introduced from northeast Brazil [101]. The African ZIKV lineage is endemic to West Africa, including neighbouring Senegal [102], and was not the strain responsible in Cabo Verde; the appearance of the Asian rather than the regionally endemic African lineage in the archipelago is plausibly explained by an earlier cryptic introduction of the Asian lineage [101]. A single confirmed case of congenital Zika syndrome in continental Africa was reported from Angola, again involving the Asian lineage.

### Viral determinants

Raulino and colleagues demonstrated in a mouse model (not human clinical data) that African ZIKV strains display higher transmissibility and foetal pathogenicity than Asian strains [30]. These experimental animal findings, while raising concern, cannot be directly extrapolated to human clinical outcomes and require confirmation in prospective human studies.

#### Host immune and genetic determinants.

No immunological or host genetic studies of ZIKV severity in African populations were identified. The near-complete absence of clinical ZIKV severity data from Africa was the single most conspicuous evidence gap identified by this review.

### Publication bias assessment

**Fig 5:**
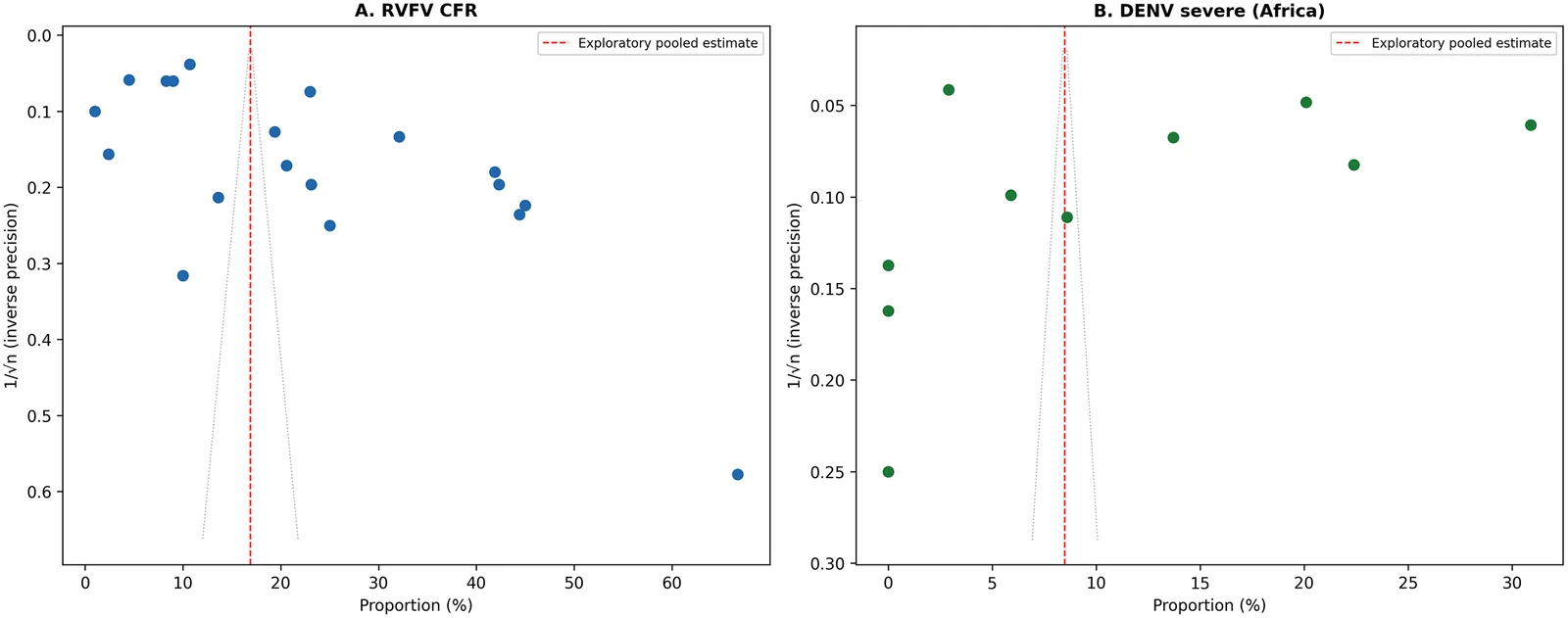
Funnel plots for the two meta-analyses. **(A)** Rift Valley fever virus case fatality rate (19 studies). **(B)** Severe dengue proportion, African studies only (10 studies). The vertical dashed line marks the exploratory pooled estimate and the dotted lines the pseudo 95% confidence limits; the vertical axis is the inverse precision 1/vn. This figure is the original work of the authors, is published under the terms of the Creative Commons Attribution 4.0 International (CC BY 4.0) licence, and has not been reproduced or adapted from any previously published source.

Funnel plot analysis was performed for both meta-analyses (Fig 5). For the RVFV case fatality rate, Egger’s regression test [89] indicated statistically significant asymmetry (intercept = 2.50, p < 0.001; funnel plot asymmetry was considered significant at p < 0.10, following the recommendation of Egger et al., given the limited statistical power of this test with fewer than 20 studies), with smaller studies tending to report higher CFRs. For the DENV severe proportion (Africa only), Egger’s test suggested potential asymmetry (intercept = −0.42, p = 0.021), though the small number of studies (n = 10) limits the power of this test. Given these findings, the pooled estimates from both meta-analyses are best regarded as indicative summaries rather than precise parameters, and readers should attend to the individual study estimates and subgroup analyses when drawing inferences.

### Cross-cutting themes

#### Co-infection and diagnostic overlap.

Malaria-arbovirus co-infection was documented in 80% of studies that assessed for it [1,2,38,44,48,56,58,64,88]. The overlapping clinical presentations of malaria, dengue, and chikungunya, all of which cause acute febrile illness, have clear diagnostic consequences: in settings where malaria rapid diagnostic testing is the default (and often only) diagnostic modality, arboviral infections are systematically missed [3,103]. Specifically, Baba et al. detected arboviral antibodies in 25% of 236 febrile patients in Maiduguri using ELISA [16]; Ayorinde et al. found dengue IgM in 17% of 504 malaria-positive patients in Lagos [78]; Monamele et al. identified DENV NS1 antigen in 15% of 200 children with confirmed malaria in Yaoundé [44]; and Galani et al. reported co-infection rates of 38% among 150 febrile patients using multiplex RT-PCR in Douala [104]. The wide range in reported co-infection rates (15–40%) reflects differences in diagnostic methods (serology vs. antigen detection vs. RT-PCR), study populations (paediatric vs. all ages), and geographic settings. The immunological consequences of co-infection, whether Plasmodium parasitaemia modulates the severity of concurrent arboviral disease, were not formally assessed in any included study, representing a substantial gap.

#### Flavivirus cross-reactivity.

**Fig 6:**
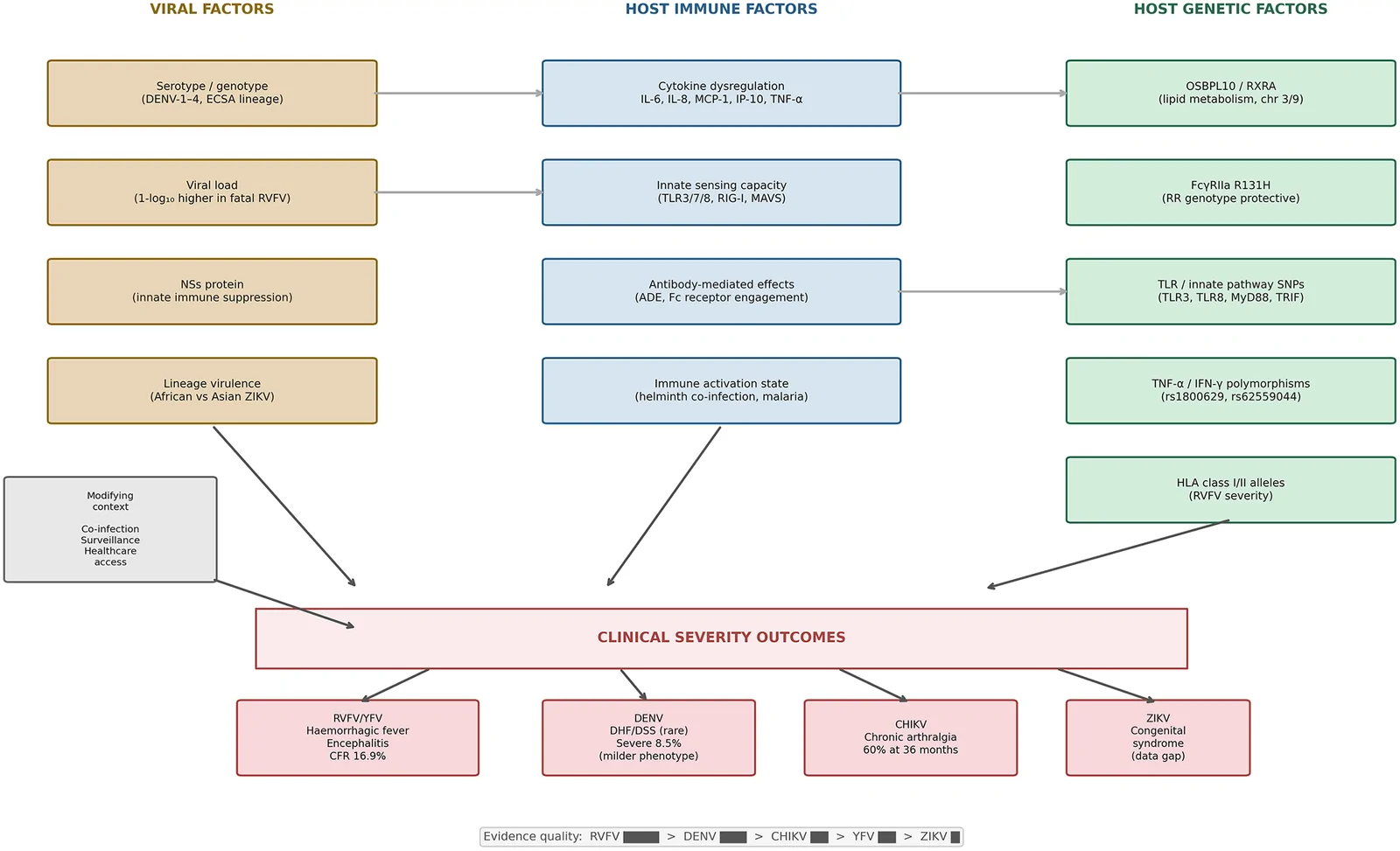
Conceptual framework of arboviral severity determinants in African populations. Viral, host immune and host genetic factors identified across the five arboviruses, with the modifying context of co-infection, surveillance and healthcare access, and their relation to the clinical severity outcomes recorded for each virus. The bar at the foot of the figure ranks the relative depth of the evidence base by virus. This figure is the original work of the authors, is published under the terms of the Creative Commons Attribution 4.0 International (CC BY 4.0) licence, and has not been reproduced or adapted from any previously published source.

The co-circulation of DENV, YFV, ZIKV, and West Nile virus across much of Africa creates a complex immunological landscape in which prior flavivirus exposure could plausibly either protect against or exacerbate subsequent heterologous infection [64,72]. Several studies noted high background flavivirus seropositivity that complicated serological diagnosis [29,72]. However, no included study directly examined whether prior YFV vaccination or natural flavivirus infection modulated the severity of a subsequent heterologous infection in an African cohort. The relationships among viral, host immune, and host genetic determinants across the five arboviruses are summarised in Fig 6.

## Discussion

This review is, to our knowledge, the first to provide a comparative synthesis across all five major arboviruses in African populations. It includes 154 studies from 38 countries and identifies several findings that merit particular attention. The very high heterogeneity observed in both meta-analyses (I^2^ > 85%) indicates that the pooled estimates should be interpreted primarily as indicative summaries of the available evidence rather than precise epidemiological parameters, and readers should attend to the subgroup and sensitivity analyses when drawing inferences. The certainty of evidence for both pooled outcomes was rated as very low by GRADE criteria (S5 Table), reflecting serious concerns about risk of bias, inconsistency, indirectness, and suspected publication bias.

Perhaps the most consequential observation is the convergence on inflammatory cytokine dysregulation as both a marker and possible mediator of fatal disease across different arboviruses. The profiles associated with fatal RVFV (elevated IL-6, IL-8, MCP-1, IP-10, IL-10; decreased RANTES) [19] and fatal YFV (elevated IL-6, TNF-α, MCP-1, IP-10, IL-1Ra) [25] show considerable overlap. This convergence suggests that the terminal pathway to multi-organ failure may be shared across pathogens, a hypothesis with therapeutic implications. In settings where rapid aetiological diagnosis is unavailable, treatments targeting common downstream inflammatory mediators could, in principle, benefit patients regardless of the specific causative virus. Whether this can be translated into clinical practice will require prospective intervention trials, which have yet to be conducted for any arbovirus in an African setting.

The putative genetic protection of African ancestry against severe dengue is, in our assessment, one of the more suggestive, though still preliminary, findings to emerge from this body of literature. Three independent lines of evidence support this hypothesis: the clinical observation of milder dengue in black Tanzanians [10], the admixture-mapped identification of OSBPL10/RXRA as protective genes with functional validation [6], and the FcγRIIa polymorphism data from Cuban cohorts [66]. However, the FcγRIIa data derive from a sample of only 97 individuals, which is underpowered for reliable genotype-disease associations and should be regarded as hypothesis-generating rather than confirmatory [66]. Similarly, the Boillat-Blanco finding that self-identified black race was protective must be interpreted with caution: race was treated as a biological variable without acknowledging its social construction, mixed-race individuals were excluded by undisclosed criteria, and the method of racial ascertainment was not described. This does not mean that severe dengue cannot occur in African populations; the DHF cases documented in Sudan [46,105] and the four-serotype co-circulation now observed in several countries [8,9] should temper any complacency.

We recognise that ‘race’ is a social and political category, not a biological one. Greater genetic diversity exists within continental African populations than between continental groups. The observed association between self-identified black race and lower dengue severity in Dar es Salaam may reflect underlying genetic variation at specific loci, but it could equally reflect unmeasured social determinants including differences in healthcare access, nutritional status, occupational exposure, and prior pathogen experience that correlate with racial categorisation. Social dynamics may partly account for the observed severity differences: Dar es Salaam and Cuba are demographically heterogeneous settings where racial categories correlate with socioeconomic gradients that could independently influence disease outcomes. Gene-environment interactions, including the influence of social conditions on epigenetic modifications and immune priming, add further complexity. Until studies explicitly measure and characterise social determinants alongside genetic data, the relative contribution of biological versus social factors will remain unclear.

The available evidence is consistent with the possibility that the lower severity burden observed in Africa is not entirely attributable to surveillance deficiencies. The practical implications would be substantial: if African populations are indeed partly protected against DHF, then the risk-benefit calculus for dengue vaccine deployment in Africa could differ from that in Asia. Alternative explanations including underdiagnosis of severe cases, surveillance limitations, differential healthcare access, and differences in circulating serotype prevalence may also contribute to the observed severity patterns, and the relative contribution of genetic versus environmental factors cannot be disentangled from the available data.

For RVFV, the host determinant evidence is the most mature. The combination of cytokine profiling [19,20,84,86], viral load quantification [19], and host genetic studies [18,106] provides a coherent model in which the capacity to mount an early, appropriately calibrated innate immune response, rather than either an exuberant or a suppressed one, determines survival.

The CHIKV data highlight a severity paradigm qualitatively distinct from the other four viruses. Rather than acute organ failure, the principal morbidity is chronic arthralgia that can persist for years. The 60% arthralgia rate at 36 months in the La Réunion cohort [24], the demonstration of persistent symptoms at 3–5 years in South Africa Table 1, and the identification of age and sex as independent predictors of chronicity [23] provide a basis for clinical counselling and post-outbreak follow-up. It must be acknowledged, however, that the most detailed longitudinal data derive from La Réunion, an overseas French territory with healthcare resources, including access to anti-inflammatory treatment and specialist rheumatology services, that are not representative of most continental African countries. Whether similar chronicity rates apply to recent outbreaks in Sudan [94], Ethiopia, and the DRC remains unknown. This representativeness limitation substantially constrains the generalisability of the CHIKV chronicity evidence to the continent.

The paucity of ZIKV data from Africa warrants emphasis. The virus was first isolated on the continent in 1947, serological evidence of circulation has been documented across West, Central, and East Africa for decades [29], and mouse-model data suggest African strains may be more pathogenic than those responsible for the 2015–2016 Americas epidemic [30]. Yet we found only 9 studies meeting our inclusion criteria, and none provided clinical severity data from the African lineage in a natural human infection cohort.

### Limitations

Several limitations should be noted. The predominance of cross-sectional and outbreak investigation designs constrained NOS scores and limited our ability to assess causal relationships between determinants and outcomes. Only 3 of 154 studies were classified as low risk of bias (NOS ≥ 7). Because individual patient data were not available for any included study, cross-study confounder adjustment could not be performed. Observed severity differences across populations may therefore reflect unmeasured confounding by age, comorbidities, healthcare access, or prior immune exposure, and the relative contributions of genetic versus environmental factors cannot be disentangled at the study level. The scope of meta-analysis was restricted to two virus-outcome combinations (RVFV case fatality and DENV severe disease proportion); even within these pooled analyses, heterogeneity was substantial (I^2^ = 88.4% and 95.7%, respectively). The DerSimonian-Laird variance estimator may underestimate between-study variance when heterogeneity is high [107]; consequently, confidence intervals around the pooled estimates may be narrower than warranted. Future updates of this review should consider GLMM-based approaches. Egger’s test indicated significant funnel plot asymmetry for the RVFV meta-analysis, consistent with possible publication bias or genuine small-study effects. The CHIKV evidence base is heavily dependent on data from La Réunion, whose healthcare infrastructure differs substantially from continental African settings, limiting the generalisability of chronicity estimates. Heterogeneity in diagnostic methods from clinical diagnosis alone to RT-PCR confirmation creates a further source of misclassification. The inclusion of Cuban admixture studies as comparator data, while methodologically justified for examining the role of African ancestry in dengue protection, introduces a population and epidemiological context that differs substantially from continental Africa. The certainty of evidence for both pooled meta-analytic outcomes was rated very low under the GRADE framework (S5 Table), and this low certainty is itself a limitation that should temper the weight placed on the pooled values throughout.

### Implications for research and policy

Given that the certainty of evidence for both pooled outcomes was rated as very low by GRADE criteria, the following research priorities should be understood as directions warranting further investigation rather than as recommendations for immediate policy change. Several priorities emerge. First, prospective multi-site cohort studies examining clinical and immunological outcomes of arboviral infection across diverse African settings are needed to replace the current reliance on cross-sectional outbreak data. Second, the preliminary protective effect of African ancestry on dengue severity warrants direct investigation in African cohorts using genome-wide association designs coupled with functional assays, a study that, to our knowledge, has not been conducted on the continent. Third, the ZIKV evidence gap demands enhanced clinical surveillance and, where feasible, pregnancy registries in areas of known circulation. Fourth, the shared cytokine signatures across severe RVFV and YFV point towards host-directed therapeutic strategies, potentially cytokine-modulating agents, that may merit investigation. Fifth, the impact of prior YFV vaccination on subsequent dengue or Zika outcomes in African populations is entirely unstudied and should be a research priority as YF vaccination campaigns expand.

## Conclusions

This systematic review of 154 studies identifies distinct immunopathological signatures associated with arboviral disease severity in African populations. These include convergent inflammatory cytokine dysregulation in fatal RVFV and YFV infection, preliminary, hypothesis-generating evidence for a genetically mediated protective effect of African ancestry against severe dengue that requires direct validation through large-scale genome-wide association studies in continental African cohorts, and a high burden of chronic arthropathy following CHIKV infection (though predominantly characterised in a non-continental African setting). The evidence base remains heavily weighted towards descriptive epidemiology. The near-complete absence of clinical ZIKV severity data from Africa, the limited understanding of flavivirus cross-reactive immunity in co-endemic settings, and the scarcity of prospective multi-site studies represent the most pressing gaps. Climate-driven expansion of Aedes and Culex vector ranges [108,109] may bring these arboviruses into African regions where population immunity is low and health systems are unprepared. The severity determinants identified in this review, including host genetic polymorphisms in innate immune pathways and the immunological consequences of sequential flavivirus exposure, may be relevant to understanding clinical outcomes in these newly exposed populations.

## Methods

### Protocol and registration

This systematic review adhered to the Preferred Reporting Items for Systematic Reviews and Meta-Analyses (PRISMA) 2020 statement [32]. The protocol was registered prospectively on PROSPERO (CRD420261334251) on 7 March 2026, before the literature search commenced. Database searching began on 8 March 2026. The completed PRISMA 2020 checklist is provided as S2 Table.

### Population, Exposure, Comparison, Outcome (PECO)

Population: Humans infected with DENV, CHIKV, YFV, RVFV, or ZIKV in African populations or populations of documented African ancestry. Exposure: Natural infection with one or more of the five target arboviruses. Comparison: Between severity strata (mild vs. severe/fatal) within and across arboviruses; secondary comparison with non-African cohort data where available. Outcome: Clinical severity (hospitalisation, haemorrhagic manifestations, organ involvement, neurological complications, chronic arthralgia, mortality) and viral/host immune/genetic determinants of severity. Eligible designs: Cross-sectional, cohort, case-control, outbreak investigation, and case series with ≥5 patients.

### Search strategy

We searched PubMed/MEDLINE, Scopus, Web of Science, and the WHO Global Index Medicus from inception through March 2026. The complete search strategy, including Boolean operators, MeSH headings, and database-specific syntax for all four databases, is provided in S4 Table. Search terms combined virus-specific identifiers (dengue, chikungunya, “yellow fever”, “Rift Valley fever”, Zika) with clinical outcome terms (severity, fatal, haemorrhagic, encephalitis, arthralgia, hospitalisation, “case fatality”) and immune determinant terms (cytokine, interferon, antibody, “T cell”, polymorphism, genotype, “viral load”, serotype), restricted geographically to Africa. Grey literature sources included WHO outbreak situation reports, ProMED alerts, and Africa CDC bulletins. Reference lists of included studies and relevant systematic reviews were hand-searched. No language restrictions were applied.

### Eligibility criteria

Studies were included if they reported on human infection with DENV, CHIKV, YFV, RVFV, or ZIKV in African populations or populations of documented African ancestry; provided data on clinical severity outcomes or on viral or host immune determinants of disease outcome; and constituted original research (cross-sectional, cohort, case-control, outbreak investigation, or case series with ≥5 patients). The threshold of five patients was selected because individual case reports and very small series lack the denominators required to estimate disease proportions and are prone to extreme selection bias. This threshold is consistent with prior arbovirus systematic reviews [110]. Exclusion criteria were: seroprevalence studies without clinical outcome data; vector or animal studies without human correlates; vaccine immunogenicity trials without natural infection comparators; reviews, editorials, and conference abstracts; and non-African populations without documented African-ancestry genetic data.

### Rationale for inclusion of non-African diaspora populations

An exception was made for Cuban admixture-mapping studies [6,66] that specifically identified African-ancestry alleles conferring protection against dengue severity. These were judged directly relevant despite their non-African geographic setting, as they provide mechanistic data on the role of African genetic ancestry that cannot currently be obtained from continental African cohorts. These Cuban data are presented separately as comparator observations and are excluded from the primary Africa-only meta-analyses. For diaspora populations, eligibility required that African ancestry be established by genetic admixture mapping or a comparable genomic method rather than by self-reported racial category alone; where an included study used self-identified racial category, this is reported as such and read with the limitations of that method in mind.

### Study selection and data extraction

Two reviewers (PAM and VK) independently screened titles and abstracts, and disagreements were resolved by consensus. Full texts of potentially eligible articles were assessed against the pre-specified eligibility criteria. Data were extracted independently by PAM and VK using a standardised 39-field form encompassing study identification, population characteristics, sample size, age, sex, diagnostic methods, viral determinants, severity outcomes, host immune determinants, co-infection status, laboratory parameters, key severity predictors, and study limitations. The completed extraction dataset and the corresponding field definitions are provided in S3 Table.

### Quality assessment

Study quality was evaluated using the Newcastle-Ottawa Scale (NOS) (Wells GA, Shea B, O’Connell D, et al. Ottawa Hospital Research Institute) adapted for cross-sectional, cohort, and case-control designs. Scoring addressed selection (0–4 stars), comparability (0–2 stars), and outcome assessment (0–3 stars). Studies scoring ≥7 were classified as low risk of bias, 4–6 as moderate, and <4 as high risk. The certainty of evidence for meta-analytic outcomes was assessed using the Grading of Recommendations, Assessment, Development, and Evaluations (GRADE) framework [111], evaluating risk of bias, inconsistency, indirectness, imprecision, and publication bias. GRADE summary-of-findings tables are presented in S5 Table.

### Data synthesis

Results were synthesised narratively, organised by virus, and structured around three domains: clinical severity profiles, viral determinants, and host immune determinants. Where sufficient data were available, specifically, studies reporting the number of severe or fatal cases among a defined denominator of confirmed infections, random-effects meta-analysis of proportions was performed using the DerSimonian-Laird method [112] with Freeman-Tukey double arcsine transformation [113] to stabilise variances, particularly for studies with proportions near zero or one. As a sensitivity analysis, between-study variance was re-estimated using both the Paule-Mandel and restricted maximum likelihood estimators. Hartung-Knapp adjustments were additionally applied to provide more conservative confidence intervals under substantial heterogeneity. These analyses used the same Freeman-Tukey double-arcsine transformation and study-level data as the primary DerSimonian-Laird analyses. The results of the alternative-estimator analyses are presented in S6 Table. The Freeman-Tukey approach was selected because several included studies reported proportions near zero, which standard logit or log transformations handle poorly. We acknowledge that generalised linear mixed models (GLMMs) offer theoretical advantages, including estimation on the original scale and potentially more natural handling of between-study variance [114,115]. The limitations of the DerSimonian-Laird variance estimator under high heterogeneity [107,116] are acknowledged, and we emphasise throughout that pooled estimates should be interpreted as indicative summaries rather than precise parameters. All analyses were conducted in Python 3.11 using custom scripts built on NumPy (v1.26) for array operations and SciPy (v1.12) for statistical functions. Forest plots and funnel plots were generated using matplotlib (v3.8). The complete analytical code is provided as S1 File. Heterogeneity was quantified using the I^2^ statistic [117], Cochran’s Q test, and the between-study variance (τ^2^).

Studies with zero events were retained in the analysis, as the Freeman-Tukey transformation accommodates zero cells without requiring continuity corrections. Pre-specified subgroup analyses were conducted by geographic region and diagnostic method. Sensitivity analyses included leave-one-out analysis and exclusion of small studies (n < 20) and case series. Publication bias was assessed by visual inspection of funnel plots and Egger’s regression test [89]. Funnel plot asymmetry was considered statistically significant at p < 0.10, following the recommendation of Egger et al., given the limited power of this test with fewer than 20 studies. For CHIKV, YFV, and ZIKV, heterogeneity in study designs, diagnostic methods, and severity definitions precluded formal pooling, and results were synthesised narratively.

### Handling of missing data

Studies with incomplete severity numerator/denominator data were included in the narrative synthesis but excluded from meta-analysis. No data imputation was performed. Where studies reported severity outcomes using different definitions (e.g., WHO 1997 vs. 2009 dengue severity classifications), the classification used by the original authors was retained.

## Supporting information

S1 Fig Subgroup analyses for RVFV case fatality rate and DENV severe disease proportion. (A) RVFV CFR by geographic region. (B) RVFV CFR by diagnostic method. (C) DENV severe proportion by region, Africa only. (D) DENV severe proportion by diagnostic method, Africa only. This figure is the original work of the authors and is published under the terms of the Creative Commons Attribution 4.0 International (CC BY 4.0) licence.
(TIFF)

S1 Table Characteristics of the 154 included studies and summary of full-text exclusion categories and counts, sorted by virus. (XLSX)

S2 Table Completed PRISMA 2020 checklist. (DOCX)

S3 Table Completed 39-field extraction dataset for all included studies, with field definitions and coding instructions. (XLSX)

S4 Table Complete search strategy with Boolean operators, MeSH headings, and database-specific syntax for all four databases. (XLSX)

S5 Table GRADE summary-of-findings table for RVFV case fatality rate and DENV severe disease proportion. (XLSX)

S6 Table Sensitivity analyses using Paule-Mandel and restricted maximum likelihood estimators, with and without Hartung-Knapp adjustment, for the RVFV case fatality rate and DENV severe disease proportion meta-analyses. (DOCX)

S1 File Python analysis scripts for meta-analysis, subgroup analysis, sensitivity analysis, forest plots, and funnel plots. (PY)
