# Supplementary material for: Viral and host determinants of arboviral disease severity in African populations: A systematic review with meta-analysis
Source: PLoS Negl Trop Dis. 2026 Aug 20;20(8):e0014663. doi: 10.1371/journal.pntd.0014663 (PMC13515024; doi:10.1371/journal.pntd.0014663)
Supplement: Supplementary file 1 [file pntd.0014663.s009.pdf]

Comments to the Authors

General comment

1. Manuscript formatting for the review process

For ease of peer review, manuscripts submitted to *PLOS NTD* must include consecutive page and line numbering from the title page through the reference list. The absence of page and line numbering in the present submission made it difficult to locate issues precisely.

Accordingly, my comments refer to the page numbers displayed in Adobe Acrobat file, together with the relevant section and approximate paragraph. I strongly recommend correcting this in the next revised version to facilitate accurate and efficient evaluation.

Major comments

1. Abstract

The abstract is well structured, methodologically transparent, and provides a clear, quantitatively supported synthesis of the severity of arboviral diseases in African populations. It effectively highlights both convergent immunopathological mechanisms and critical evidence gaps, particularly regarding Zika virus (ZIKV) and immunological profiling. Minor revisions are recommended to improve wording precision, avoid potential overinterpretation of comparative findings, and correct minor typographical inconsistencies. For example, please verify the notation used for heterogeneity. In the draft manuscript table on page 1 in Adobe Acrobat view document, the heterogeneity statistic appears twice as "I2" rather than "I^2." This may simply reflect a copy-paste or formatting problem introduced during submission to the editorial manager system, but it should nevertheless be corrected.

2. Introduction

Several issues of presentation and contextualization should be addressed to improve the clarity and positioning of the manuscript.

First, the manuscript would benefit from stronger epidemiological contextualization. The authors should provide basic information on the prevalence and mortality of the arboviral diseases under study in the African countries represented in the review, ideally with brief comparisons to Asia and Latin America. This would help situate the study within a broader global perspective and clarify why the African context deserves focused synthesis.

Second, a concise clinical description of the symptom profiles associated with each of the five arboviral infections would improve accessibility, especially for non-specialist readers. In addition, a brief conceptual introduction to PRISMA, PROSPERO, and the Newcastle-Ottawa Scale would be useful, together with a clear explanation of why these frameworks and tools were selected and how they fit the design of the present study.

Third, references are needed to support the statements made in the second part of the first paragraph of the Introduction. More broadly, the manuscript would benefit from a more comprehensive exposition of previous meta-analyses and related reviews so that the present study can be more clearly positioned within the existing literature.

Finally, the Introduction should articulate the manuscript's specific contribution with greater precision. The distinctiveness of the study, its explicit research purpose, and the question it seeks to answer should all be stated more directly.

3. Methods

All statistical procedures described in the Methods should be appropriately referenced so that readers can consult the original methodological literature and assess the suitability of the chosen approaches. In addition, each procedure should be clearly justified and explained in accessible language to improve transparency and interpretability.

For example, the authors should clarify why they selected the Freeman-Tukey double arcsine transformation rather than alternative approaches such as generalized linear mixed models. This rationale should be explicit in the text. Depending on the structure of the data and the research objective, GLMMs may offer advantages, including modeling on the original scale without transformation, more natural handling of zero counts, more robust treatment of between-study variability, and potentially more accurate estimation under heterogeneity. Even if the authors ultimately retain the Freeman-Tukey approach, the choice should be justified with methodological references.

This concern is especially relevant in the Data synthesis subsection. As noted above, the statistical procedures should not only be named but also justified and properly cited. The rationale for the Freeman-Tukey double arcsine transformation should therefore be made explicit and supported by the appropriate methodological literature.

In addition, the use of Python 3.11, including NumPy and SciPy, is currently under described. The manuscript should provide more detail on how these tools were used analytically. It would also substantially strengthen transparency and reproducibility if the authors were to provide the analytical code or syntax, for example in an appendix or supplementary file, so that interested readers could replicate the study in other contexts or regions. Is the full syntax set publicly available to the scientific community and the wider audience?

4. Results

The Results section represents the most critical component of the manuscript. However, its internal organization could be improved to ensure greater consistency across the five arboviruses, particularly on pages 16 to 19. For example, dengue is structured using three analytic categories: clinical severity proportions, viral determinants, and host immune or genetic determinants. In contrast, Rift Valley fever virus includes an additional mixed category addressing viral–host interaction determinants, whereas chikungunya, yellow fever, and Zika are not presented within a consistently parallel framework. Although Table 2 provides a clear and effective summary of key viral and host immune determinants of severity across viruses, the narrative description of the Results (pages 16 to 19) lacks structural uniformity.

General issues regarding figures presentation

All figure numbers should be carefully checked, as it appears inconsistent with the order in which the figures are cited in the manuscript. For example, what is currently labeled Figure 2 may need to be renumbered as Figure 3. The authors should verify all figures numbers systematically.

[redacted]

In addition, the font size across the figures and supplementary figures is currently too small for comfortable reading. All figures should be revised to improve readability, including larger fonts and closer compliance with *PLOS NTD* figure presentation standards.

The manuscript would also benefit from improved visualization of study locations. A georeferenced map of Africa, for example using coordinate-based visualization tools, could represent the included countries more precisely and more informatively than a conventional static map generated by AI. The current AI-generated image of Africa should be improved in both quality and contemporary visual presentation.

General comments on publication bias assessment section

Egger’s regression test, like all the other statistical procedures used in the study, should be appropriately referenced using the original or standard methodological sources. The authors should also specify the bibliographic basis or interpretive criteria used to evaluate Egger’s test results. This recommendation applies broadly across the statistical methods section and not only to publication bias assessment section.

General comments on cross-cutting themes

This section is well written, clear, and valuable. It adds transparency to the manuscript, particularly with regard to co-infection, diagnostic overlap, and flavivirus cross-reactivity.

Other issues in Results

On page 17, the manuscript reports lower dengue severity estimates in African studies, around 6.7% to 10.0%, than in Asian cohorts, around 20% to 30%. This cross-regional comparison would benefit from additional supporting references from the Asian literature to substantiate the stated range. Moreover, given the substantial heterogeneity reported, with I^2 values above 95%, the authors should clarify how comparable these estimates truly are across regions, especially in light of possible differences in case definitions, surveillance systems, and population immunity.

On page 18, there are also minor language issues that must be corrected: “Third, In a Cuban cohort…” should read “Third, [**in**] a Cuban cohort…”. Likewise, “Taken together, taken together, these data are…” should be corrected to “[**Taken together**], these data are…”.

5. Discussion and conclusion

In my opinion, the Discussion is one of the strongest sections of the manuscript. It stands out in both quality and clarity relative to earlier sections, which do not yet reach the same level. That said, however, there are still a few grammatical and syntactic issues that should be corrected.

For example, on page 27, the opening paragraph of the Discussion contains a sentence with missing verbal structure and awkward construction:

“This review to our knowledge the first to provide a comparative synthesis across all five major arboviruses in African populations included 155 studies from 38 countries and identified several findings that, we believe, merit particular attention.”

This could be improved as follows:

“This review is, to our knowledge, the first to provide a comparative synthesis across all

five major arboviruses in African populations. It includes 155 studies from 38 countries and identifies several findings that merit particular attention."

Also on page 27, the sentence beginning "Three independent lines of evidence…" appears to be missing a verb. A more complete construction would be something like: "Three independent lines of evidence are the clinical observation of milder dengue in black Tanzanians [48], the admixture-mapped identification of OSBPL10/RXRA as protective genes with functional validation [47], …"

These are relatively small language issues, but they should be corrected to match the level expected in a Q1 publication.

6. References

The reference list departs substantially from Vancouver style and requires systematic revision. Many references are missing essential bibliographic elements, including volume, issue, page range, or article identifiers. The use of "et al." also appears inconsistent with Vancouver conventions, particularly where full author listing may be required under journal style. In addition, title capitalization often follows APA-like patterns rather than Vancouver sentence-style capitalization. There also appear to be duplicate references, for example references 14 and 15, and several entries are incomplete or lack DOI information.

Overall, the references require a careful and comprehensive review to meet *PLOS NTD* standards. Examples of entries that appear to contain one or more of these problems include references 1 to 5, 9, 12, 14, 15, 18, 21, 24, 27, 33, 35, 38, 43, 47, 50, 53, 62, 65, 71, 80, and 94 to 95. In its current form, the manuscript is not yet suitable for acceptance and requires major revision in this section as well.

Minor comments

7. General comments regarding English language

The authors are advised to revise the manuscript carefully for wording, grammar, and English syntax. The remaining errors are individually minor, but their cumulative presence is not acceptable for a Q1 journal publication. The use of AI-assisted language tools or professional scientific language editing may help eliminate these issues.

Final recommendation

I recommend **major revision**, based on the points detailed above.
