# Supplementary material for: Viral and host determinants of arboviral disease severity in African populations: A systematic review with meta-analysis
Source: PLoS Negl Trop Dis. 2026 Aug 20;20(8):e0014663. doi: 10.1371/journal.pntd.0014663 (PMC13515024; doi:10.1371/journal.pntd.0014663)
Supplement: Supplementary file 2 [file pntd.0014663.s011.pdf]

Review Report 2 – Manuscript PNTD-D-26-00516

Dr. Sujatha Sunil, Ph.D.
Section Editor
PLOS Neglected Tropical Diseases

1 July 2026

Dear Dr. Sunil,

Please find attached my Review Report 2 for manuscript PNTD-D-26-00516.

I have carefully evaluated the revised manuscript with particular attention to the concerns and recommendations raised in my original review. Overall, I appreciate the considerable effort invested by the authors in revising their work. In my opinion, the manuscript has improved substantially in methodological transparency, statistical justification, reproducibility, and overall scientific quality. My detailed assessment is presented below.

Methods

The Introduction has been strengthened through a clearer epidemiological context, a more explicit statement of the study objectives, and a better articulation of the novelty and significance of the work. The Methods section has also improved considerably.

The authors now provide more comprehensive descriptions and appropriate references for the statistical procedures employed, including a well-reasoned justification for the use of the Freeman–Tukey transformation and a thoughtful discussion of alternative analytical approaches. The inclusion of additional methodological details, together with the availability of analytical code and supplementary materials, substantially enhances the transparency and reproducibility of the study.

Overall, I consider that the principal methodological and statistical concerns raised in my previous review have been satisfactorily addressed.

Results

The Results section has been substantially reorganized, improving clarity and consistency across the five arboviral diseases included in the review. The presentation of the pooled estimates is now more balanced, and the authors appropriately acknowledge the implications of the high between-study heterogeneity observed in several analyses.

The discussion of publication bias and the interpretation of Egger's regression test have also been strengthened through additional methodological explanation and appropriate referencing.

I have no additional major concerns regarding the presentation or interpretation of the results.

Conclusions

The Conclusions are now better aligned with the evidence presented in the manuscript. The revised discussion appropriately reflects the limitations of the available evidence, the uncertainty associated with several pooled estimates, and the need for additional high-quality studies.

The manuscript also provides a clearer discussion of the public health implications while avoiding the overinterpretation identified during the initial review.

Overall, I believe that the conclusions are adequately supported by the data presented.

Editorial and Data Presentation Modifications

Only a few relatively minor issues remain before publication.

I recommend a final editorial review of the reference list to ensure complete compliance with Vancouver style and consistency across all citations.

Please verify that all supplementary files are correctly cited, consistently numbered, and accurately cross-referenced throughout the manuscript.

A final proofreading of the English language would further improve readability by correcting a few remaining stylistic inconsistencies.

During production, it would be advisable to verify that all figures retain adequate font size, resolution, and readability in the final published version.

These recommendations are editorial in nature and do not affect the scientific validity of the manuscript.

Summary and General Comments

The authors have responded carefully and constructively to the concerns raised in my previous review. The revised manuscript demonstrates substantial improvements, particularly in methodological transparency, statistical justification, reproducibility, and the interpretation of highly heterogeneous evidence.

The expanded methodological descriptions, strengthened statistical justification, improved discussion of heterogeneity and study limitations, clearer articulation of the research objectives, and provision of supplementary analytical materials have significantly enhanced the scientific quality of the manuscript.

After evaluating the revised version, I consider that the principal concerns raised in my original review have been satisfactorily addressed. The remaining observations are limited to minor editorial and presentation refinements that can be addressed during the final revision.

Overall, this manuscript represents a valuable contribution to the literature on arboviral disease epidemiology in Africa and provides a comprehensive synthesis that will be of interest to researchers, epidemiologists, and public health professionals.

Recommendation: Minor Revision.

Confidential Comments to the Editor

I have no confidential concerns regarding research integrity, publication ethics, or the peer-review process.

Thank you for the opportunity to review this interesting manuscript. I hope that my comments will be helpful in informing your editorial decision.

Sincerely,

Benicio Gutiérrez-Doña, B.Sc., Lic., M.Sc., Ph.D.
Research Professor of Psychology
Psychological and Behavioral Sciences Research Program
Universidad Estatal a Distancia (UNED)
San José, Costa Rica
