# Supplementary material for: Viral and host determinants of arboviral disease severity in African populations: A systematic review with meta-analysis
Source: PLoS Negl Trop Dis. 2026 Aug 20;20(8):e0014663. doi: 10.1371/journal.pntd.0014663 (PMC13515024; doi:10.1371/journal.pntd.0014663)
Supplement: Supplementary file 3 [file pntd.0014663.s003.docx]

**Supplementary Table S2. PRISMA 2020 Checklist**

*Manuscript: Viral and host determinants of arboviral disease severity in African populations: a systematic review with meta-analysis*

From: Page MJ, McKenzie JE, Bossuyt PM, et al. The PRISMA 2020 statement: an updated guideline for reporting systematic reviews. BMJ 2021;372:n71.

| **Section / Topic** | **#** | **Checklist Item** | **Location Where Reported** |
| --- | --- | --- | --- |
| TITLE | 1 | Identify the report as a systematic review, meta-analysis, or both. | Title page, line 1 |
| ABSTRACT | 2 | See PRISMA for Abstracts checklist. | Abstract, pages 1–2 |
| **INTRODUCTION** |  |  |  |
| Rationale | 3 | Describe the rationale for the review in the context of existing knowledge. | Introduction, paragraphs 1–6 |
| Objectives | 4 | Provide an explicit statement of the objective(s) or question(s) the review addresses. | Introduction, final paragraph: “We set out to address a specific question...” |
| **METHODS** |  |  |  |
| Eligibility criteria | 5 | Specify the inclusion and exclusion criteria for the review and how studies were grouped for the syntheses. | Methods: Eligibility criteria; PECO box |
| Information sources | 6 | Specify all databases, registers, websites, organisations, reference lists and other sources searched or consulted. | Methods: Search strategy; Supplementary Table S4 |
| Search strategy | 7 | Present the full search strategies for all databases, registers and websites, including any filters and limits used. | Methods: Search strategy (summary); full strategies with Boolean operators, MeSH terms, and database-specific syntax in Supplementary Table S4 |
| Selection process | 8 | Specify the methods used to decide whether a study met the inclusion criteria, including how many reviewers screened and the agreement metric. | Methods: Study selection. Two reviewers (PAM, VK) independently screened; κ = 0.82 (title/abstract), κ = 0.91 (full-text) |
| Data collection process | 9 | Specify the methods used to collect data from reports, including how many reviewers collected data and any processes for obtaining or confirming data. | Methods: Data extraction. Standardised 39-field form (Supplementary Table S3). Extractors: PAM and VK. |
| Data items | 10 | List and define all outcomes for which data were sought and all other variables for which data were extracted. | Methods: Data extraction; Supplementary Table S3 (full 39-field form with definitions) |
| Study risk of bias assessment | 11 | Specify the methods used to assess risk of bias in the included studies. | Methods: Quality assessment. Newcastle–Ottawa Scale adapted for cross-sectional, cohort, and case-control designs. |
| Effect measures | 12 | Specify for each outcome the effect measure(s) used in the synthesis or presentation of results. | Methods: Data synthesis. Proportions with 95% CI; Freeman–Tukey double arcsine transformation. |
| Synthesis methods | 13 | Describe the processes used to decide which studies were eligible for each synthesis and describe the synthesis methods. | Methods: Data synthesis. DerSimonian–Laird random-effects model; subgroup analyses by region and diagnostic method; sensitivity analyses (leave-one-out, exclusion of small studies); narrative synthesis for CHIKV, YFV, ZIKV. |
| Reporting bias assessment | 14 | Describe any methods used to assess risk of bias due to missing results in a synthesis. | Methods: Data synthesis. Funnel plots and Egger’s regression test (Egger et al. BMJ 1997). Significance threshold p < 0.10. |
| Certainty assessment | 15 | Describe any methods used to assess certainty (or confidence) in the body of evidence for an outcome. | Certainty of evidence was assessed using the GRADE framework for the two outcomes included in quantitative synthesis: RVFV case fatality rate and DENV severe disease proportion. Both outcomes were rated as very low certainty. Reported in Methods, Results, Discussion, and Supplementary Table S5. |
| **RESULTS** |  |  |  |
| Study selection | 16 | Describe the results of the search and selection process. | Results: Study selection and characteristics; Figure 1 (PRISMA flow diagram) |
| Study characteristics | 17 | Cite each included study and present its characteristics. | Results: Study selection; Table 1; Supplementary Table S1 (all 155 studies with characteristics) |
| Risk of bias in studies | 18 | Present assessments of risk of bias for each included study. | Results: Study selection (median NOS 5; IQR 4–5). Individual NOS scores in extraction workbook and Supplementary Table S1. |
| Results of individual studies | 19 | For all outcomes, present for each study: a summary of the data, the effect estimate and its precision. | Results sections by virus; Figures 2–3 (individual study estimates); Table 2 |
| Results of syntheses | 20 | For each synthesis, briefly summarise the characteristics and risk of bias among contributing studies. | Results: RVFV CFR 16.9% (95% CI 11.6–22.8; I² = 88.4%); DENV severe 8.5% (95% CI 3.0–16.2; I² = 95.7%); subgroup analyses; Supplementary Figure S1 |
| Reporting biases | 21 | Present assessments of risk of bias due to missing results for each synthesis. | Results: Publication bias assessment; Figure 4 (funnel plots with pseudo-95% CI lines); Egger’s test results |
| Certainty of evidence | 22 | Present assessments of certainty (or confidence) in the body of evidence for each outcome assessed. | GRADE assessment was presented for RVFV case fatality rate and DENV severe disease proportion. Both outcomes were rated as very low certainty. Full details are provided in Supplementary Table S5. |
| **DISCUSSION** |  |  |  |
| Discussion | 23a | Provide a general interpretation of the results in the context of other evidence. | Discussion, paragraphs 1–8 |
| Discussion | 23b | Discuss any limitations of the evidence included in the review. | Discussion: Limitations subsection |
| Discussion | 23c | Discuss any limitations of the review processes used. | Discussion: Limitations subsection (DerSimonian–Laird limitations, diagnostic heterogeneity, absence of IPD) |
| Discussion | 23d | Discuss implications of the results for practice, policy, and future research. | Discussion: Implications for research and policy. All recommendations framed as conditional on GRADE very low certainty. |
| **OTHER INFORMATION** |  |  |  |
| Registration and protocol | 24a | Provide registration information for the review, including register name and registration number. | Methods: Protocol and registration (PROSPERO CRD420261334251) |
| Registration and protocol | 24b | Indicate where the review protocol can be accessed, or state that a protocol was not prepared. | Protocol accessible on PROSPERO (CRD420261334251) |
| Support | 25 | Describe sources of financial or non-financial support for the review. | Declarations: Funding. WHO/TDR Grant 1013487-0 to PAM. Funders had no role in study design, data collection, analysis, decision to publish, or manuscript preparation. |
| Competing interests | 26 | Declare any competing interests of review authors. | Declarations: “The authors have declared that no competing interests exist.” |
| Availability of data, code, and other materials | 27 | Report which of the following are publicly available and where they can be found. | Declarations: Data availability. All supplementary materials provided with submission: extraction workbook, search strategy (Table S4), PRISMA checklist (Table S2), extraction form (Table S3), GRADE table (Table S5), Python scripts (Supplementary File S1). |
