# Supplementary material for: Viral and host determinants of arboviral disease severity in African populations: A systematic review with meta-analysis
Source: PLoS Negl Trop Dis. 2026 Aug 20;20(8):e0014663. doi: 10.1371/journal.pntd.0014663 (PMC13515024; doi:10.1371/journal.pntd.0014663)
Supplement: Supplementary file 4 [file pntd.0014663.s007.docx]

**Supplementary Table S6. Sensitivity analyses using alternative between-study variance estimators for the RVFV and DENV meta-analyses**

| **Outcome** | **Variance estimator** | **Confidence-interval method** | **Pooled estimate** | **95% CI** |
| --- | --- | --- | --- | --- |
| RVFV case fatality rate | DerSimonian-Laird (primary analysis) | Normal approximation | 16.9% | 11.6-22.8% |
| RVFV case fatality rate | Paule-Mandel | Normal approximation | 17.9% | 10.9-25.9% |
| RVFV case fatality rate | REML | Normal approximation | 17.8% | 11.0-25.7% |
| RVFV case fatality rate | Paule-Mandel | Hartung-Knapp adjustment | 17.9% | 10.5-26.5% |
| RVFV case fatality rate | REML | Hartung-Knapp adjustment | 17.8% | 10.5-26.4% |
| DENV severe disease proportion | DerSimonian-Laird (primary analysis) | Normal approximation | 8.5% | 3.0-16.2% |
| DENV severe disease proportion | Paule-Mandel | Normal approximation | 8.4% | 3.0-16.1% |
| DENV severe disease proportion | REML | Normal approximation | 8.4% | 3.0-16.1% |
| DENV severe disease proportion | Paule-Mandel | Hartung-Knapp adjustment | 8.4% | 2.3-17.4% |
| DENV severe disease proportion | REML | Hartung-Knapp adjustment | 8.4% | 2.3-17.4% |

**Footnotes:** CI, confidence interval; DENV, dengue virus; REML, restricted maximum likelihood; RVFV, Rift Valley fever virus. All analyses used the Freeman-Tukey double-arcsine transformation. The alternative estimators produced pooled point estimates consistent with the primary DerSimonian-Laird analyses, although the Hartung-Knapp adjustment produced wider confidence intervals.

**Methodological note:** DerSimonian-Laird was the primary between-study variance estimator. Paule-Mandel and REML were applied as sensitivity analyses, and Hartung-Knapp confidence intervals were calculated as a more conservative assessment under substantial heterogeneity.
