# Supplementary material for: Viral and host determinants of arboviral disease severity in African populations: A systematic review with meta-analysis
Source: PLoS Negl Trop Dis. 2026 Aug 20;20(8):e0014663. doi: 10.1371/journal.pntd.0014663 (PMC13515024; doi:10.1371/journal.pntd.0014663)
Supplement: Supplementary file 5 [file pntd.0014663.s010.docx]

**Response to Editor and Reviewers' Comments**

Manuscript PNTD-D-26-00516

Viral and host determinants of arboviral disease severity in African populations:

a systematic review with meta-analysis

Dear Editors,

We thank the editors and all three reviewers for their careful and constructive evaluation of our manuscript. The comments have materially strengthened the work. Below, we provide a detailed point-by-point response. All changes are marked in the revised manuscript using colour-coded text: **blue for Editor responses**, **dark green for Reviewer 1**, **purple for Reviewer 2**, and **dark red for Reviewer 3**.

# EDITOR COMMENTS

***Comment:*** *Please ensure that the CRediT author contributions listed for every co-author are completed accurately and in full.*

**Response:** Done. We have updated the CRediT author contributions in the submission form to reflect each co-author’s specific roles. The revised statement reads: “Conceptualization: PAM, AK. Data curation: PAM, VK, PA, DCI. Formal analysis: PAM. Investigation: PAM, VK, PA, DAD, DCI. Methodology: PAM, AK. Project administration: PAM. Supervision: AK. Validation: VK, PA. Writing – original draft: PAM. Writing – review & editing: PAM, VK, PA, DAD, DCI, AK.”

***Comment:*** *Your manuscript’s sections are not in the correct order. Please amend to the following order: Abstract, Introduction, Results, Discussion, and Methods.*

**Response:** Corrected. The revised manuscript now follows the PLOS NTD section order: Abstract, Introduction, Results, Discussion, Methods. We have verified that all internal cross-references remain accurate after reordering.

***Comment:*** *Please upload all main figures as separate Figure files in .tif or .eps format.*

**Response:** All six main figures and the supplementary figure have been regenerated as high-resolution (300 dpi) .tif files using matplotlib and exported at print-quality dimensions. They will be uploaded as separate files in the revised submission.

***Comment:*** *Tables should not be uploaded as individual files. Please include the Tables in your manuscript file as editable, cell-based objects.*

**Response:** Tables 1 and 2 have been integrated directly into the manuscript as editable, cell-based objects. The separate table file has been removed from the submission.

***Comment:*** *We have noticed that you have uploaded Supporting Information files, but you have not included a list of legends. Please add a full list of legends for your Supporting Information files after the references list.*

**Response:** A complete Supporting Information legends section has been appended after the reference list in the revised manuscript. This covers: Figure S1 (subgroup forest plots), Table S1 (complete list of included and excluded studies with exclusion reasons), Table S2 (PRISMA 2020 checklist), Table S3 (data extraction form), and Supplementary File S1 (Python analysis scripts).

***Comment:*** *Please amend your detailed Financial Disclosure statement.*

**Response:** The revised statement reads: “This work was supported by the World Health Organization Special Programme for Research and Training in Tropical Diseases (WHO/TDR), Grant 1013487-0, awarded to PAM. The funders had no role in study design, data collection and analysis, decision to publish, or preparation of the manuscript. No authors received salary from the funder.”

***Comment:*** *Please ensure your manuscript or supplementary information includes the required data availability items: a numbered table of all studies identified (including excluded ones with reasons), all extracted data, risk of bias assessments, and an explanation of how missing data were handled.*

**Response:** We have prepared the following supplementary materials to satisfy all data availability requirements: (1) Table S1: a numbered table of all 297 screened records, indicating inclusion/exclusion status and, for each excluded study, the specific reason for exclusion; (2) The complete 39-field data extraction workbook is provided as a supplementary Excel file (the file already uploaded as Copy_of_master_extraction_final.xlsx); (3) Newcastle–Ottawa Scale scores for all 155 included studies are tabulated within the extraction workbook, specifying each domain score; (4) A “Handling of missing data” paragraph has been added to the Methods section explaining that studies with incomplete severity numerator/denominator data were included in the narrative synthesis but excluded from meta-analysis, and that no imputation was performed. Data extractors (PAM and VK) and extraction dates are recorded in the workbook.

***Comment:*** *Figure 6: Please provide a direct link to the base layer of the map and the terms of use / license information.*

**Response:** The revised Figure 6 has been regenerated programmatically in Python using the Natural Earth 1:110m Admin-0 Countries shapefile (public domain, https://www.naturalearthdata.com/about/terms-of-use/). The figure legend now includes: “Base map: Natural Earth (public domain, naturalearthdata.com).” The previous AI-generated map has been replaced. We can confirm full compatibility with the CC BY 4.0 licence.

***Comment:*** *Please ensure that the files are uploaded in correct numerical order; that captions are included for all six figures; and that the competing interests statement aligns with journal style.*

**Response:** (a) File upload order has been verified and corrected. (b) Captions are present for all six main figures and the supplementary figure. (c) The competing interests statement has been amended to: “The authors have declared that no competing interests exist.”

# REVIEWER 1 (Anonymous)

## General Comment: Manuscript Formatting

***Comment:*** *Manuscripts submitted to PLOS NTD must include consecutive page and line numbering. The absence of page and line numbering made it difficult to locate issues precisely.*

**Response:** We apologise for this oversight. The revised manuscript now includes continuous page numbering (bottom centre) and line numbering (every 5 lines, restarting each page) throughout all sections from the title page to the reference list.

## Major Comment 1: Abstract

***Comment:*** *The abstract is well structured. Minor revisions are recommended to improve wording precision, avoid potential overinterpretation, and correct typographical inconsistencies. For example, the heterogeneity statistic appears as “I2” rather than “I².”*

**Response:** We appreciate this positive assessment. The following corrections have been made in the Abstract: (a) All instances of “I2” have been changed to “I²” throughout the manuscript for proper typographic rendering. (b) We have added hedging language around the DENV–Asia comparison, replacing “suggestive of lower severity” with: “though any comparison with Asian cohort estimates must be interpreted with considerable caution given the substantial heterogeneity (I² > 95%), which masks meaningful clinical and methodological diversity across studies.” (c) The numerical inconsistencies flagged by Reviewer 2 (see below) have been resolved; the Abstract now reports the corrected, internally consistent estimates throughout.

## Major Comment 2: Introduction

***Comment:*** *Several issues: (1) The manuscript needs stronger epidemiological contextualisation with prevalence/mortality data and comparisons to Asia and Latin America. (2) A concise clinical description of symptom profiles for each virus is needed. (3) A brief conceptual introduction to PRISMA, PROSPERO, and the NOS is needed. (4) References to support statements in the first paragraph. (5) More comprehensive exposition of previous meta-analyses. (6) A more precise articulation of the study’s specific contribution.*

**Response:** We have substantially expanded the Introduction to address each of these points: (1) Epidemiological context: A new paragraph has been added providing WHO/Global Burden of Disease estimates for dengue (390 million annual infections globally, 96 million symptomatic), RVFV outbreak mortality data from East Africa, and regional comparison figures. We cite Bhatt et al. (Nature 2013) and recent WHO arboviral burden estimates to anchor the African burden within its global context. (2) Clinical profiles: A condensed clinical summary paragraph now describes the predominant manifestations for each virus — dengue haemorrhagic fever and shock syndrome for DENV; haemorrhagic fever, encephalitis, and retinitis for RVFV; chronic polyarthralgia for CHIKV; viscerotropic hepatorenal disease for YFV; and congenital syndrome and microcephaly for ZIKV. (3) Methodological framework: We now briefly introduce PRISMA 2020 as the reporting standard for systematic reviews, PROSPERO as the international prospective register for review protocols, and the Newcastle–Ottawa Scale as a validated quality assessment tool for observational studies, with appropriate citations (Page et al. BMJ 2021; Wells et al. Ottawa Hospital Research Institute). (4) Missing references: The second paragraph of the Introduction (on arboviral co-circulation and surveillance gaps) now includes supporting references [Bhatt et al. 2013; Brady et al. 2012; Messina et al. 2019]. (5) Prior reviews: We have added a paragraph citing existing virus-specific systematic reviews (Simmonds et al. 2017 for DENV severity; Baudin et al. 2020 for RVFV case fatality; Paixao et al. 2016 for ZIKV complications) and explicitly state how the present review fills the gap left by the absence of a cross-cutting, multi-arbovirus comparative synthesis for African populations. (6) The final paragraph of the Introduction now reads: “We therefore set out to address a specific question: what viral, host immune, and host genetic factors have been identified as determinants of clinical severity for DENV, CHIKV, YFV, RVFV, and ZIKV infections in African and African-ancestry populations, and where do the most consequential evidence gaps lie?”

## Major Comment 3: Methods

***Comment:*** *All statistical procedures should be referenced. The rationale for the Freeman–Tukey double arcsine transformation over alternatives such as GLMMs should be explicit. The use of Python 3.11 (NumPy, SciPy) is under-described. Analytical code or syntax should be provided.*

**Response:** We have addressed each point as follows: (a) Statistical procedure references: The DerSimonian–Laird method is now cited (DerSimonian & Laird, Controlled Clinical Trials 1986). The Freeman–Tukey double arcsine transformation is cited (Freeman & Tukey, Annals of Mathematical Statistics 1950; Miller, 1978). Egger’s regression test is cited (Egger et al., BMJ 1997). Cochran’s Q and I² are cited (Higgins et al., BMJ 2003). (b) Justification for Freeman–Tukey: We have added an explicit rationale paragraph: “The Freeman–Tukey double arcsine transformation was selected because several included studies reported proportions near zero, which standard logit or log transformations handle poorly. This approach stabilises variances across the full range of proportions and accommodates zero-event studies without requiring ad hoc continuity corrections. We acknowledge that generalised linear mixed models (GLMMs) offer theoretical advantages, including estimation on the original scale and potentially more natural handling of between-study variance (Stijnen et al. 2010; Schwarzer et al. 2019). However, we retained the Freeman–Tukey approach to maintain comparability with prior arbovirus meta-analyses (Baudin et al. 2020; Baba et al. 2022). The limitations of the DerSimonian–Laird variance estimator under high heterogeneity (Veroniki et al. 2016) are acknowledged, and we have accordingly emphasised throughout the manuscript that the pooled estimates should be interpreted as indicative summaries rather than precise parameters.” We also cite the cautionary methodological literature flagged by Reviewer 3 (Schwarzer et al. 2019, doi:10.1002/jrsm.1348; Barker et al. 2021, doi:10.1002/hsr2.178). (c) Python methodology: We have expanded the description to read: “All statistical analyses were conducted in Python 3.11. Meta-analytic pooling was implemented using custom scripts built on NumPy (v1.26) for array operations and SciPy (v1.12) for statistical functions, including the Freeman–Tukey transformation, DerSimonian–Laird between-study variance estimation, inverse-variance weighting, and Egger’s regression test. Forest plots and funnel plots were generated using matplotlib (v3.8). All computational steps are fully documented.” (d) Code availability: The complete Python scripts have been deposited as Supplementary File S1, including all analytical code for meta-analysis, subgroup analysis, sensitivity analysis, forest plot generation, and funnel plot generation. We have also added a Data Availability statement directing readers to these scripts.

## Major Comment 4: Results

***Comment:*** *The Results section’s internal organisation could be improved to ensure greater consistency across the five arboviruses. Dengue uses three analytic categories; RVFV includes an additional mixed category; CHIKV, YFV, and ZIKV are not presented within a consistently parallel framework.*

**Response:** We agree that structural parallelism strengthens readability. The Results section has been reorganised so that each virus is now presented using a uniform three-part framework: (1) Clinical severity profiles, (2) Viral determinants, and (3) Host immune and genetic determinants. For RVFV, the previously separate “viral–host interaction” subsection has been integrated into the host immune determinants section. For CHIKV, YFV, and ZIKV, we have added explicit subheadings corresponding to each of the three domains, even where data are sparse in which case we state that no studies were identified (e.g., “No host genetic association studies for CHIKV in African populations were identified”). This parallel structure makes cross-viral comparisons more transparent.

## General Issues: Figures Presentation

***Comment:*** *Figure numbers should be checked for consistency with in-text citation order. Font sizes are too small. The AI-generated map of Africa should be improved. A georeferenced map is recommended.*

**Response:** (a) Figure numbering: We have systematically renumbered all figures to match the order of first citation in the text. The corrected order is: Figure 1 (PRISMA), Figure 2 (RVFV forest plot), Figure 3 (DENV forest plot), Figure 4 (funnel plots), Figure 5 (conceptual framework), Figure 6 (geographic distribution map). (b) Font size: All figures have been regenerated with a minimum font size of 8 pt for labels and 10 pt for axis titles, compliant with PLOS figure standards. (c) Map: As noted in our response to the Editor, Figure 6 has been completely regenerated programmatically using Natural Earth public domain shapefiles in Python (geopandas + matplotlib). Country-level study counts are plotted using a graduated colour scheme on actual geographic boundaries, replacing the previous AI-generated illustration. The revised figure provides accurate georeferencing and is fully compatible with CC BY 4.0.

## Publication Bias Assessment

***Comment:*** *Egger’s regression test should be appropriately referenced. The authors should specify the bibliographic basis or interpretive criteria used to evaluate Egger’s test results.*

**Response:** Egger’s test is now cited (Egger M, Davey Smith G, Schneider M, Minder C. Bias in meta-analysis detected by a simple, graphical test. BMJ. 1997;315:629–634). We have also added the interpretive criterion: “Funnel plot asymmetry was considered statistically significant at p < 0.10, following the recommendation of Egger et al. (1997), given the limited statistical power of this test with fewer than 20 studies.”

## Major Comment 5: Discussion and Conclusion

***Comment:*** *The Discussion is one of the strongest sections. However, there are grammatical and syntactic issues, including a sentence with missing verbal structure on page 27.*

**Response:** Thank you. We have corrected all flagged issues: (a) The opening sentence has been revised to: “This review is, to our knowledge, the first to provide a comparative synthesis across all five major arboviruses in African populations. It includes 155 studies from 38 countries and identifies several findings that merit particular attention.” (b) The sentence beginning “Three independent lines of evidence…” has been restructured to: “Three independent lines of evidence support this hypothesis: the clinical observation of milder dengue in black Tanzanians [48], the admixture-mapped identification of OSBPL10/RXRA as protective genes with functional validation [47], and the FcγRIIa polymorphism data from Cuban cohorts [14,15].” (c) A thorough language edit has been performed across the entire Discussion, correcting the remaining grammatical and syntactic issues. We engaged a professional scientific language editor for the final round of revision.

## Major Comment 6: References

***Comment:*** *The reference list departs substantially from Vancouver style. Many references are missing essential elements. Duplicate references (e.g., 14 and 15). Inconsistent use of “et al.” and title capitalisation.*

**Response:** We have systematically revised the entire reference list to comply with Vancouver style as specified by PLOS NTD. Specific corrections include: (a) Full author listing: All references now list up to six authors followed by “et al.” where applicable, consistent with PLOS conventions. (b) Missing elements: Volume, issue, page ranges, and article identifiers (DOIs or PMIDs) have been added to all references where available. We cross-checked each entry against PubMed and CrossRef. (c) Duplicate entries: References 14 and 15, which cited the same García et al. (2010) DOI, have been consolidated into a single entry. All subsequent reference numbers have been renumbered accordingly, and all in-text citations have been updated. (d) Title capitalisation: Corrected to sentence case throughout, as required by Vancouver style. (e) We systematically addressed the specific references flagged (1–5, 9, 12, 14, 15, 18, 21, 24, 27, 33, 35, 38, 43, 47, 50, 53, 62, 65, 71, 80, 94–95) and verified all remaining entries.

## Minor Comment 7: English Language

***Comment:*** *The authors are advised to revise carefully for wording, grammar, and English syntax.*

**Response:** The manuscript has undergone a thorough language revision. A professional scientific language editor reviewed the full text. We have also used Grammarly Pro as a secondary check. Specific recurring issues corrected include: inconsistent hyphenation of compound modifiers, occasional misplaced commas in restrictive clauses, and a few instances of British/American English inconsistency (we have standardised to British English throughout, consistent with PLOS NTD convention).

# REVIEWER 2 (Nnamso W. Ikpeida)

## Methods: PECO Framework

***Comment:*** *Structured Population, Exposure, Comparison, Outcome and eligible study designs need to be clearly defined in organised form rather than having to be rebuilt from prose.*

**Response:** We have added a structured PECO box in the Methods section: Population: Humans infected with DENV, CHIKV, YFV, RVFV, or ZIKV in African populations or populations of documented African ancestry. Exposure: Natural infection with one or more of the five target arboviruses. Comparison: Between severity strata (mild vs. severe/fatal) within and across arboviruses; secondary comparison with non-African cohort data where available. Outcome: Clinical severity (hospitalisation, haemorrhagic manifestations, organ involvement, neurological complications, chronic arthralgia, mortality) and viral/host immune/genetic determinants of severity. Eligible designs: Cross-sectional, cohort, case-control, outbreak investigation, and case series with ≥5 patients.

## Methods: Search Strategy

***Comment:*** *No Boolean operators, MeSH terms, or database syntax included in the search string. A complete search plan should be given as an additional appendix.*

**Response:** The complete search strategy, including Boolean operators, MeSH headings, and database-specific syntax for all four databases (PubMed/MEDLINE, Scopus, Web of Science, WHO Global Index Medicus), has been prepared as Supplementary Table S4. For example, the PubMed strategy reads: (("dengue"[MeSH] OR "chikungunya"[MeSH] OR "yellow fever"[MeSH] OR "Rift Valley fever"[MeSH] OR "Zika virus infection"[MeSH]) OR (dengue OR chikungunya OR "yellow fever" OR "Rift Valley fever" OR Zika)) AND ((severity OR fatal* OR haemorrhag* OR hemorrhag* OR encephalitis OR arthralgia OR hospitali* OR "case fatality") OR (cytokine* OR interferon OR antibod* OR "T cell" OR polymorphism* OR genotype* OR "viral load" OR serotype*)) AND (Africa[MeSH] OR [54 individual African country names]) Adapted versions for Scopus, Web of Science, and GIM are provided in full. Grey literature sources (WHO, ProMED, Africa CDC) are also documented.

## Methods: Eligibility Criteria (≥5 Patients)

***Comment:*** *The ≥5 number of patients as a case series inclusion criterion is not justified. Small case series add heterogeneity at a disproportionate rate to their epidemiological signal.*

**Response:** We agree that this threshold warranted explicit justification. We have added the following rationale: “Case series with fewer than five patients were excluded because individual case reports and very small series lack the denominators required to estimate disease proportions and are prone to extreme selection bias. The threshold of five patients is consistent with prior arbovirus systematic reviews (Baudin et al. 2020; Bettis et al. 2022) and balances the need to capture rare outbreak data from under-studied African settings against the disproportionate heterogeneity introduced by very small samples.” Additionally, we note that our sensitivity analysis excluding small studies (n < 20) and case series demonstrated that the pooled meta-analytic estimates were not materially altered, providing reassurance that these small studies did not distort the quantitative findings.

## Methods: Screening Agreement

***Comment:*** *Cohen’s kappa or percentage agreement at the two screening stages is not reported. This is not insignificant with 297 screened records and 191 full texts.*

**Response:** We appreciate this point. Inter-rater agreement has been calculated retrospectively from the screening records and is now reported: “Inter-rater agreement was substantial at the title/abstract stage (κ = 0.82, 95% CI 0.75–0.89) and near-perfect at the full-text stage (κ = 0.91, 95% CI 0.84–0.98). Disagreements (n = 14 at title/abstract; n = 3 at full text) were resolved by consensus discussion between PAM and VK, with AK consulted for the three full-text disagreements.”

## Methods: Data Extraction Instrument

***Comment:*** *The 39-field extraction form is described but not reproduced or given as a supplement. Readers cannot determine consistent application across heterogeneous study designs.*

**Response:** The complete 39-field data extraction form has been added as Supplementary Table S3, listing every field with its definition, response categories, and coding instructions. The completed extraction workbook (Copy_of_master_extraction_final.xlsx) is also provided as supplementary data.

## Methods: Quality Assessment — GRADE

***Comment:*** *The Newcastle–Ottawa Scale is implemented but GRADE evaluation of overall certainty of evidence is entirely absent. The authors recommend policy on vaccines, host-directed therapy, and pregnancy registries based on what they describe as mainly moderate-quality cross-sectional evidence. GRADE was created specifically to fill this gap. Its absence is the most significant methodological lack of the manuscript.*

**Response:** We accept this criticism. In the revised manuscript, we have taken a two-pronged approach: (1) We have performed a GRADE assessment for the two outcomes where quantitative synthesis was conducted (RVFV case fatality rate and DENV severe disease proportion). Both were rated as “Very Low” certainty, downgraded for serious risk of bias (predominantly cross-sectional designs), serious inconsistency (I² > 85%), serious indirectness (heterogeneous case definitions and populations), and suspected publication bias (RVFV). The GRADE summary-of-findings table has been added as Supplementary Table S5 and is referenced in the Results. (2) In light of this assessment, we have substantially tempered the policy implications language. The relevant paragraph in the Discussion now reads: “Given that the certainty of evidence for both pooled outcomes was rated as very low by GRADE criteria, the following research priorities should be understood as directions warranting further investigation rather than as recommendations for immediate policy change.” Each specific suggestion (vaccine risk–benefit recalibration, host-directed therapy, pregnancy registries) is now explicitly framed as conditional on future higher-quality evidence. We have avoided language that could be read as actionable clinical or policy guidance.

## Results: Numerical Inconsistencies

***Comment:*** *There are numerical inconsistencies between the abstract, results text, and figure legends of both meta-analyses. For RVFV, the abstract reports 16.9% (95% CI 11.6–22.8); Figure 2 legend cites 17.4% (95% CI 11.8–23.6). For DENV: abstract states 10 African studies, 8.5% (95% CI 3.0–16.2; I² = 95.7%, Q = 210.3); Figure 3 legend states 12 studies, 11.1% (95% CI 5.1–18.7; I² = 95.0%, Q = 198.7). These are substantively different and reflect a quality control failure.*

**Response:** We acknowledge this error without reservation — it is unacceptable, and we thank the reviewer for catching it. The discrepancy arose because the figure legends were drafted from an earlier analytical iteration that included two additional studies subsequently excluded during a final eligibility re-check, while the abstract and results text were updated to reflect the final analysis. We should have caught this during our own internal review. The correct, final estimates are: • RVFV CFR: 16.9% (95% CI 11.6–22.8; I² = 88.4%, Q = 155.7, p < 0.001; 19 studies). This is reported consistently across the abstract, results text, and all figure legends in the revised manuscript. • DENV severe proportion (Africa only): 8.5% (95% CI 3.0–16.2; I² = 95.7%, Q = 210.3, p < 0.001; 10 African studies). The Cuba comparator is presented separately as stated in the results text. All figure legends, Table 1, and the abstract have been aligned to these figures. We have re-run all analyses from the final dataset and verified every reported number. The Python analysis scripts are provided as Supplementary File S1 for full reproducibility.

## Results: CHIKV Representativeness

***Comment:*** *CHIKV is overly reliant on La Réunion data — a French overseas territory whose health system is not representative of continental Africa. The one continental African study (Kassala, Sudan) gets one sentence. This representativeness issue must be foregrounded.*

**Response:** Point well taken. In the revised manuscript, we have: (a) Added a new paragraph at the start of the CHIKV section explicitly flagging this representativeness concern: “A central limitation of the CHIKV evidence base is its dependence on La Réunion, a French overseas department with a health system infrastructure, surveillance capacity, and socioeconomic context that differ markedly from most continental African settings. Findings from La Réunion cannot be extrapolated to the continent without considerable caution.” (b) Expanded the Kassala, Sudan paragraph to describe the clinical findings of Bower et al. (2021) in greater detail, including the acute clinical spectrum, the absence of longitudinal follow-up data, and the implications for understanding CHIKV burden in a resource-constrained African setting. (c) Added this issue as a named limitation in the Limitations section, rather than relegating it to a passing remark.

## Results: ZIKV Animal Data

***Comment:*** *The Raulino mouse-model data should be clearly marked as animal experimentation. Non-experts might confuse experimental findings with clinical human burden.*

**Response:** We have revised the relevant sentence to read: “Raulino and colleagues demonstrated in a mouse model (not human clinical data) that African ZIKV strains display higher transmissibility and foetal pathogenicity than Asian strains. These experimental animal findings, while raising concern, cannot be directly extrapolated to human clinical outcomes and require confirmation in prospective human studies.”

## Results: Co-infection Claims

***Comment:*** *The assertion that 15–40% of malaria patients harboured concurrent arboviral infections needs systematic synthesis specifying the studies, arboviruses, denominator populations, and diagnostic methods.*

**Response:** We agree that this claim required better structural support. We have revised this passage to provide a study-level breakdown: “Several studies from Nigeria and Cameroon documented concurrent arboviral infections among febrile patients initially diagnosed with malaria. Specifically, Baba et al. (2013) detected arboviral antibodies in 25% of 236 febrile patients in Maiduguri; Ayorinde et al. (2016) found dengue IgM in 17% of 504 malaria-positive patients in Lagos using ELISA; Monamele et al. (2018) identified DENV NS1 antigen in 15% of 200 children with confirmed malaria in Yaoundé; and Galani et al. (2020) reported co-infection rates of 38% among 150 febrile patients using multiplex RT-PCR in Douala. The wide range in reported co-infection rates (15–40%) reflects differences in diagnostic methods (serology vs. antigen detection vs. RT-PCR), study populations (paediatric vs. all ages), and geographic settings.”

## Results: PRISMA Compliance

***Comment:*** *No reference is made to the PRISMA 2020 checklist as having been filled in and submitted as a supplement. The PRISMA 2020 checklist should be filled in and sent as supplementary.*

**Response:** The completed PRISMA 2020 checklist has been prepared as Supplementary Table S2. A statement referencing its availability has been added to the Methods section: “The completed PRISMA 2020 checklist is available as Supplementary Table S2.”

## Methods: PROSPERO Registration Timing

***Comment:*** *It is not made clear whether PROSPERO registration took place prior to the literature search itself. PROSPERO stipulates prospective registration prior to both search and extraction.*

**Response:** We have clarified this in the Methods: “The protocol was registered prospectively on PROSPERO (CRD420261334251) prior to the commencement of the literature search. Registration was completed on 07 March 2026, and the database search was initiated on 08 March 2026.

## Methods: Cuban Studies Rationale

***Comment:*** *The explanation for including Cuban studies is correct but should be relocated to a specific sentence under a heading: “Rationale for inclusion of non-African populations.”*

**Response:** Done. A clearly labelled subsection “Rationale for inclusion of non-African diaspora populations” has been added within the Eligibility Criteria section, explaining why the Cuban admixture-mapping studies were included as comparator data and how they were handled analytically (presented separately from the primary Africa-only analyses).

## Data Presentation: Tables

***Comment:*** *Tables 1 and 2 are mentioned throughout but were not completely included in the submitted text. Their accuracy and completeness cannot be evaluated.*

**Response:** Both tables are now fully integrated into the manuscript body as editable cell-based objects. They were previously submitted as a separate file. We apologise for the confusion this caused during review.

## Data Presentation: Proofreading

***Comment:*** *The repeated words “taken together, taken together” (DENV host immune section) should be removed.*

**Response:** Corrected. The sentence now reads: “Taken together, these data are consistent with the hypothesis that the apparently milder dengue phenotype in African populations may not be entirely attributable to surveillance deficiencies.”

## Conclusions: Genetic Protection Framing

***Comment:*** *The genetic dengue protection argument is based on indirect evidence and is hypothesis-generating, not confirmatory. This difference is not well represented in the conclusions and needs rewording.*

**Response:** We have reworded the relevant conclusion: “Suggestive, though still preliminary, evidence from clinical, admixture-mapping, and Fc receptor polymorphism data points towards a genetically mediated component of dengue protection in populations of African ancestry. These findings are hypothesis-generating and require direct validation through large-scale genome-wide association studies in continental African cohorts before any clinical or policy inferences can be drawn.”

## Conclusions: Climate Change Paragraph

***Comment:*** *The climate change paragraph appears as a generic appended statement. Unless related to the findings of the review, it should be integrated with specific epidemiological projections.*

**Response:** We have revised this paragraph to tie it directly to the review findings: “Climate-driven expansion of Aedes and Culex vector ranges (Kraemer et al. 2019; Ryan et al. 2019) may bring these arboviruses into African regions where population immunity is low and health systems are unprepared. The severity determinants identified in this review including host genetic polymorphisms in innate immune pathways and the immunological consequences of sequential flavivirus exposure will be relevant to understanding clinical outcomes in these newly exposed populations.”

## Conclusions: Individual Patient Data Limitation

***Comment:*** *Two gaps in the limitations: (1) the absence of individual patient data prevents cross-study confounder adjustment, and (2) this should be stated as a formal limitation, not a caveat.*

**Response:** We have added the following to the Limitations section: “Because individual patient data were not available for any included study, cross-study confounder adjustment could not be performed. Observed severity differences across populations may therefore reflect unmeasured confounding by age, comorbidities, healthcare access, or prior immune exposure, and the relative contributions of genetic versus environmental factors cannot be disentangled at the study level.” This is now stated as a formal limitation rather than a parenthetical caveat.

# REVIEWER 3 (Anonymous)

## Meta-Analysis: Heterogeneity and Pooled Estimates

***Comment:*** *Given the very high I², I am concerned about misinterpretation of the pooled results. Caution should be flagged early and consistently. I strongly recommend presenting forest plots without pooling. The DerSimonian–Laird estimator can underestimate between-study variance when heterogeneity is high. GLMMs have been recommended instead. Where Egger’s test flags significant publication bias, describing estimates as “indicative rather than definitive” is insufficient; removing the pooled estimate would be more defensible.*

**Response:** We have given this recommendation careful thought and have made the following changes: (1) Cautionary framing: We now flag interpretive caution immediately after presenting I² values, rather than deferring to the Discussion. The revised text reads: “Substantial heterogeneity was observed (I² = 88.4%), indicating that this pooled average masks considerable clinical and methodological diversity across studies. It should be interpreted as an approximate central tendency of the available data rather than a reliable epidemiological parameter.” Equivalent language has been added for the DENV analysis. (2) Forest plots: We have revised the forest plots to present the individual study estimates without the pooled diamond. The pooled estimate is instead reported only in the text, accompanied by the caveat that it represents a summary statistic under conditions of extreme heterogeneity. The I², Q statistic, and p-value are displayed below each plot, but the pooled diamond has been removed. This approach aligns with the reviewer’s recommendation and with the treatment we already applied to CHIKV, YFV, and ZIKV. (3) Statistical estimator: We have added explicit discussion of the DerSimonian–Laird estimator’s limitations under high heterogeneity, citing Veroniki et al. (2016) and the references provided by the reviewer (Schwarzer et al. 2019; Barker et al. 2021). We now state: “The DerSimonian–Laird estimator may underestimate between-study variance when heterogeneity is high; consequently, confidence intervals around the pooled estimates may be narrower than warranted. Future updates of this review should consider GLMM-based approaches.” (4) Publication bias: For the RVFV analysis, where Egger’s test was significant (p < 0.001), we now state: “Given both the extreme heterogeneity and the significant funnel plot asymmetry, the pooled RVFV case fatality estimate should not be treated as a reliable summary parameter. The individual study estimates and their variation are more informative than the pooled value.” (5) Funnel plot: Figure 4A has been revised to include 95% confidence interval diagonal lines (pseudo-95% CI limits) to better visualise the expected distribution under the null hypothesis of no publication bias, as suggested.

## García et al. (2010): Sample Size Concerns

***Comment:*** *García et al. had only 67 females and 30 males far too small for causal inferences or generalisability. Findings should be flagged as candidate gene hypotheses, not reliable evidence. This is precisely the kind of preliminary signal that justifies calls for large-scale GWAS.*

**Response:** We fully agree. The revised Discussion now includes: “The FcγRIIa polymorphism data from García et al. [14] derive from a sample of only 97 individuals (67 female, 30 male), which is underpowered for reliable detection of genotype–disease associations. These findings should be regarded as generating a candidate gene hypothesis rather than providing confirmatory evidence. The modest sample size, combined with the absence of genome-wide approaches, means that unmeasured confounding and population stratification cannot be excluded. This observation underscores the need for adequately powered GWAS in continental African populations with severe dengue as the primary outcome a study that, to our knowledge, has not yet been conducted.”

## Boillat-Blanco et al. (2018): Race as Social Construct

***Comment:*** *The original authors did not elucidate how race was determined, presented race entirely as a biological construct, and excluded mixed-race individuals by undisclosed criteria. Results should be interpreted with great caution and on an exploratory basis only; causal inferences are not warranted. “Black race” should not be treated as a biological category; it is a social and political construction.*

**Response:** This is a critically important point, and we are grateful for the reviewer’s precision on this issue. We have made the following changes: (1) In the Results section, we now explicitly note the methodological concerns: “Boillat-Blanco et al. reported that self-identified black race was independently protective against severe dengue after multivariate adjustment. However, the method by which race was ascertained was not described, mixed-race individuals were excluded from the analysis by undisclosed criteria, and the study treated race as a biological variable without acknowledging its social construction. These methodological limitations substantially constrain the strength of any causal inference.” (2) In the Discussion, we have added a paragraph explicitly addressing race as a social construct: “We recognise that ‘race’ is a social and political category, not a biological one. Greater genetic diversity exists within continental African populations than between continental groups. The observed association between self-identified black race and lower dengue severity in Dar es Salaam may reflect underlying genetic variation at specific loci (as suggested by the admixture-mapping data), but it could equally reflect unmeasured social determinants including differences in healthcare access, nutritional status, occupational exposure, and prior pathogen experience that correlate with racial categorisation. Disentangling genetic from social and environmental contributions will require studies that directly genotype candidate loci in well-characterised cohorts with careful measurement of socioeconomic and behavioural confounders, rather than relying on self-reported race as a proxy.” (3) All language referring to this finding throughout the manuscript now frames it as exploratory and hypothesis-generating rather than confirmatory.

## Social Determinants and Gene–Environment Interactions

***Comment:*** *Social dynamics likely influence the differences observed, including in comparative populations with African ancestry outside Africa. Social determinants may be driving perceived protection as much as biological ones. Gene–environment interactions warrant consideration.*

**Response:** We have added a new paragraph in the Discussion addressing social determinants: “Social dynamics may partly account for the observed severity differences. Dar es Salaam and Cuba the two settings where African ancestry appeared protective are demographically heterogeneous cities where racial categories correlate with socioeconomic gradients, healthcare access patterns, and residential exposures that could independently influence disease severity. Gene–environment interactions, including the influence of social conditions on epigenetic modifications and immune priming, add further complexity. Until studies explicitly measure and adjust for these social determinants alongside genetic data, the relative contribution of biological versus social factors to the observed severity differences will remain unclear.”

## Minor: Typographical Corrections

***Comment:*** *Multiple minor typographical issues: missing commas (“cross-cutting themes [,] the role of African genetic ancestry”; eligibility criteria comma; data synthesis comma), inconsistent quoting in search terms, “Taken together, taken together” repetition, and Figure 1 PRISMA box rearrangement suggestion.*

**Response:** All flagged typographical issues have been corrected: (a) Missing commas inserted at all three flagged locations. (b) Search strategy terms now use consistent quotation marks throughout. (c) The “Taken together, taken together” duplication has been removed. (d) Figure 1 (PRISMA): We have revised the flow diagram to disaggregate the virus counts within the qualitative synthesis box (DENV: 58, RVFV: 31, Multiple: 27, CHIKV: 15, YFV: 15, ZIKV: 9) and added a parallel disaggregation for the meta-analysis box (RVFV: 19, DENV: 10). The final box listing all viruses has been removed as suggested.

## Suggestion: Tabular Study Overview by Disease

***Comment:*** *Create a figure/box with the studies included in the systematic analysis, sorted by disease, including year of publication, region, title, etc.*

**Response:** We have prepared Supplementary Table S1, which lists all 155 included studies sorted by virus, with columns for: first author, year, country, region, study design, sample size, diagnostic method, key severity outcome, and NOS score. Excluded studies are listed in a separate sheet with exclusion reasons. This table is referenced in the Results section and available as supplementary material.

We trust that these revisions satisfactorily address all concerns raised. We are grateful for the thorough and constructive review, which has substantially strengthened the manuscript. We remain available to address any further queries the editors or reviewers may have.

Sincerely,

Peter Asaga Mac, on behalf of all authors
