# Supplementary material for: Viral and host determinants of arboviral disease severity in African populations: A systematic review with meta-analysis
Source: PLoS Negl Trop Dis. 2026 Aug 20;20(8):e0014663. doi: 10.1371/journal.pntd.0014663 (PMC13515024; doi:10.1371/journal.pntd.0014663)
Supplement: Supplementary file 6 [file pntd.0014663.s012.docx]

**Response to Reviewers**

**Manuscript PNTD-D-26-00516R1**

*Viral and host determinants of arboviral disease severity in African populations: a systematic review with meta-analysis*

Journal: PLOS Neglected Tropical Diseases

Dear Dr Sunil,

Thank you for the opportunity to revise our manuscript, and please pass on our thanks to the three reviewers. Their careful reading has strengthened the paper. Reviewers 1 and 2 recommended minor revision and raised editorial and clarifying points, which we address below. Reviewer 3 renewed a substantive concern about the pooled estimates, which we have considered carefully and answer in full below. We set out our replies point by point. Each reviewer is answered in a distinct colour, and the same colour is used to mark the corresponding change in the revised manuscript: Reviewer 1 in blue, Reviewer 2 in red, and Reviewer 3 in purple.

Two conventions apply throughout the revision. British spelling is used throughout, and we have removed en and em dashes from the running text in line with the journal style we are following, retaining hyphens only where a term is conventionally hyphenated.

**Response to the Editorial Office and Journal Requirements**

***Journal requirement 1 (Financial Disclosure).*** *The Financial Disclosure states WHO/TDR, while the submission form records grant 1013487-0. Please confirm the full and correct funding information, the order of funders, and whether the funder had any role.*

**Response.** There is a single funder, so no ordering question arises. The complete and correct statement, as it now reads in the Declarations, is that the work was supported by the World Health Organization Special Programme for Research and Training in Tropical Diseases (WHO/TDR), grant 1013487-0, awarded to PAM. The funder had no role in study design, data collection and analysis, the decision to publish, or preparation of the manuscript, and no author received salary from the funder. We will confirm this wording to the editorial office by return email as requested.

***Journal requirement 2 (Data availability).*** *Please provide a numbered table of all studies identified, including those excluded with reasons; a table of all extracted data with extractor names and dates, eligibility confirmation, and everything needed to replicate the analyses; risk of bias and certainty assessments for each study or outcome; and an explanation of how missing data were handled.*

**Response. Supplementary Table S1 contains the 155 study records included in the review, with study characteristics and study-level Newcastle-Ottawa scores, together with the full-text exclusion categories and counts. Supplementary Table S3 contains the completed 39-field extraction dataset and field definitions, with data extraction undertaken independently by PAM and VK. Supplementary Table S5 gives the GRADE certainty assessment for each pooled outcome, including judgements across all domains. The Methods explain that studies with incomplete numerator or denominator data were retained in the narrative synthesis but excluded from pooling, and that no imputation was performed.**

**Response to Reviewer 1 (shown in blue)**

We are grateful for the positive assessment and for the recommendation of minor revision. The remaining points were editorial, and we have worked through each.

***Comment 1.1.*** *A final editorial review of the reference list to ensure consistency with Vancouver style, including journal abbreviations, punctuation, capitalization, bibliographic completeness, and DOI formatting.*

**Response.** We have gone through the reference list again for Vancouver consistency, checking abbreviated journal titles, author and page formatting, and DOI presentation, and correcting completeness where entries were short. As one example, reference 60 (Wauquier and colleagues) was missing its DOI, which we have supplied and formatted to match the surrounding entries.

***Text inserted or revised in the manuscript:*** “PLoS Negl Trop Dis. 2019;13(3):e0007224. doi:10.1371/journal.pntd.0007224.”

***Comment 1.2.*** *Please verify that all supplementary files are correctly cited, consistently numbered, and accurately cross-referenced throughout the manuscript.*

**Response. We checked the supplementary numbering and cross-references. Figure S1 and Tables S1 to S5 are cited consistently. The supplementary package includes the study list, completed extraction dataset, PRISMA checklist, search strategy, GRADE assessment, and the analysis scripts identified as Supplementary File S1.**

***Text inserted or revised in the manuscript:*** “The full list of screened records, with inclusion status and the reason for each exclusion, is provided as Supplementary Table S1.”

***Comment 1.3.*** *A final proofread of the English text would improve readability by correcting minor stylistic inconsistencies and enhancing sentence flow.*

**Response.** The manuscript has been proofread in full. Beyond the specific corrections requested by the other reviewers, we repaired several sentences where an earlier round of dash removal had left broken clauses, and we tightened a number of phrasings for flow. One example in the Introduction now reads more cleanly.

***Text inserted or revised in the manuscript:*** “The reasons for these divergent trajectories remain poorly understood, particularly in African populations.”

***Comment 1.4.*** *During production, authors should ensure that figures and tables are legible in the final publication format, including font size, axis labels, legends, and image resolution.*

**Response.** Agreed. The figures have been prepared as high-resolution TIFF files and processed through the journal NAAS tool to meet the print size and resolution requirements, and we have checked that axis labels, legends and fonts remain legible at the final column width. We will confirm the production-ready versions at acceptance.

**Response to Reviewer 2 (shown in red)**

We thank Reviewer 2 for a close and constructive reading, and for the detailed endorsement of the design and analysis. We address each point below.

***Comment 2.1 (population definition).*** *The eligibility criterion admits diaspora populations of documented African ancestry, but the Cuban cohorts used self-reported race and admixture mapping, which differ in genetic accuracy. The Methods would benefit from specifying that African ancestry should be determined by genetic admixture mapping or similar rather than by self-report.*

**Response.** We agree, and we have made the criterion explicit in the Methods. The Cuban cohorts were admitted precisely because ancestry there was established by admixture mapping rather than by self-report, and we now say so, while noting how self-identified categories, where used within included studies, are handled.

***Text inserted or revised in the manuscript:*** “For diaspora populations, eligibility required that African ancestry be established by genetic admixture mapping or a comparable genomic method rather than by self-reported racial category alone; where an included study used self-identified racial category, this is reported as such and read with the limitations of that method in mind.”

***Comment 2.2 (PROSPERO number).*** *Please ensure the PROSPERO number is correctly written and, if in error, corrected immediately. The one-day gap between registration and search start is compliant.*

**Response.** We are grateful to the reviewer for checking this. We have confirmed that the identifier is correct exactly as issued by PROSPERO, and it now reads identically in the Abstract, Introduction, Methods and Data availability statement. The impression that the format is non-standard is understandable, and worth explaining. The PROSPERO platform, relaunched for registrations from 2025 onward, assigns a longer identifier than the legacy scheme, extending the serial component by one digit. A 2026 registration such as ours therefore reads as CRD4, followed by the year, followed by a seven digit serial, giving CRD420261334251, rather than the shorter eleven digit form familiar from earlier years. The record can be verified directly at https://www.crd.york.ac.uk/PROSPERO/view/CRD420261334251. We are also grateful that the reviewer confirms the one day interval between registration and the start of the search satisfies the prospective registration requirement.

***Comment 2.3 (explicit hypothesis).*** *The main mechanistic proposition is implied rather than stated. A clear statement of the central hypothesis would help focus the Introduction, for example that African genetic ancestry is hypothesised to partially protect against severe dengue via lipid metabolism and Fc receptor pathways.*

**Response.** This is a helpful suggestion. We have added a short statement of the central proposition at the close of the Introduction, framed as a hypothesis to be weighed rather than as an assumption, so that the reader meets it plainly before the research question.

***Text inserted or revised in the manuscript:*** “One proposition recurs in the dengue literature and is worth stating plainly at the outset: that African genetic ancestry may partly protect against severe dengue through lipid metabolism and Fc receptor pathways. We treat this throughout as a hypothesis to be weighed against the evidence assembled here, not as a settled conclusion.”

***Comment 2.4 (Abstract date typo).*** *The registration date in the Abstract reads 7th Marc 2026, a typographical error for 7th March 2026. The Methods are correct.*

**Response.** Corrected in the Abstract.

***Text inserted or revised in the manuscript:*** “on 7th March 2026”

***Comment 2.5 (Introduction apostrophe).*** *The sentence beginning Five arthropod-borne viruses dengue (DENV) carries an incorrect possessive apostrophe and needs a separator.*

**Response.** Corrected. The sentence now uses a clean appositive construction without a dash, in keeping with the journal style we are following.

***Text inserted or revised in the manuscript:*** “Five arthropod-borne viruses, namely dengue (DENV), chikungunya (CHIKV), yellow fever (YFV), Rift Valley fever (RVFV), and Zika (ZIKV), together constitute one of the most pressing infectious disease threats confronting sub-Saharan Africa today.”

***Comment 2.6 (climate conclusion).*** *The word will in will be relevant to understanding clinical outcomes in these newly exposed populations should be may be relevant, to reflect the speculative nature of the extrapolation.*

**Response.** Agreed, and changed to may. The point is genuinely speculative and the softer verb is the honest one.

***Text inserted or revised in the manuscript:*** “the immunological consequences of sequential flavivirus exposure, may be relevant to understanding clinical outcomes in these newly exposed populations.”

***Comment 2.7 (PECO in Abstract).*** *PECO is used as a heading in the Methods but the acronym is not introduced where the reader first meets the methodological framing. Either spell it out on first use or avoid the acronym there.*

**Response.** We have introduced the framework, spelled out in full with the acronym, in the Abstract Methods, so that it is defined at first encounter; the Methods heading continues to carry the full term.

***Text inserted or revised in the manuscript:*** “Eligibility followed a Population, Exposure, Comparison, Outcome (PECO) framework.”

***Comment 2.8 (variance estimator).*** *The DerSimonian-Laird estimator can narrow confidence intervals under high heterogeneity, particularly for DENV. A REML or Paule-Mandel sensitivity analysis would address this without overhauling the method.*

**Response.** We thank the reviewer for this important recommendation. We have now repeated both meta-analyses using the Paule–Mandel and restricted maximum likelihood estimators for between-study variance, while retaining the Freeman–Tukey double-arcsine transformation used in the primary analysis. We additionally calculated Hartung–Knapp-adjusted confidence intervals as a more conservative assessment under substantial heterogeneity.

For RVFV, the Paule–Mandel estimate was 17.9% (95% CI 10.9–25.9) and the restricted maximum likelihood estimate was 17.8% (95% CI 11.0–25.7), compared with 16.9% (95% CI 11.6–22.8) using DerSimonian–Laird. Hartung–Knapp adjustment widened the confidence intervals to 10.5–26.5% and 10.5–26.4%, respectively.

For DENV, both alternative estimators yielded a pooled severe disease proportion of approximately 8.4% (95% CI 3.0–16.1), compared with 8.5% (95% CI 3.0–16.2) in the primary analysis. Hartung–Knapp adjustment widened the confidence interval to 2.3–17.4%.

The alternative estimators therefore did not materially change either pooled point estimate or the substantive interpretation. They did, however, confirm that uncertainty, particularly for RVFV and under Hartung–Knapp adjustment, is wider than suggested by reliance on a single conventional random-effects analysis. These results have now been reported in the Methods and Reseults.

**Response to Reviewer 3 (shown in purple)**

We thank Reviewer 3 for a rigorous reading across both rounds, and in particular for the sustained pressure on how we present the pooled estimates. We have moved a good distance towards the reviewer position, and we set out where and how below.

***Comment 3.1 and 3.2 (pooled estimates, DENV and RVFV).*** *Given the authors own account of extreme heterogeneity, the pooled DENV and RVFV estimates are difficult to justify in the main text and invite selective citation. The individual study estimates are more informative. Consider moving the pooled results to the supplementary materials and restructuring the main text around narrative synthesis, explaining why estimates were not pooled.*

**Response.** We have thought hard about this. The manuscript already foregrounds the individual study estimates, and for RVFV it states in terms that the individual estimates and their variation are more informative than the pooled value. We have now made the same point explicit for DENV, and we state plainly that the pooled figures are kept for transparency and reproducibility rather than as parameters for cross-regional comparison. We have also added the very low GRADE certainty to the Limitations, so that the low confidence guides what the reader foregrounds (see comment 3.6). We took this middle course, keeping the values visible and fully reproducible while making unmistakably clear that they are secondary to the narrative and per-study synthesis, in part because the two reviewers who assessed the statistics directly both supported retaining the estimates with the current caveats. That said, we recognise this is a presentational judgement on which reasonable people differ, and we would be glad to relocate the two pooled estimates and their forest plots to the Supporting Information if the editor considers that the better course.

***Text inserted or revised in the manuscript:*** “As with RVFV, the individual study estimates set out in Table 1 and Figure 3 are more informative than the pooled figure and should anchor interpretation; the pooled value is retained for transparency rather than as a parameter for cross-regional comparison.”

***Comment 3.3 (CHIKV, La Réunion).*** *The introductory paragraph on CHIKV should state explicitly that findings from La Réunion cannot be extrapolated to continental Africa without significant caution, given differences in health infrastructure and access to care.*

**Response.** The CHIKV section already opens with a dedicated paragraph making this point. We have strengthened its wording to significant caution and added a closing sentence that names the specific risk, namely reading La Réunion chronicity figures as if they were continental African estimates.

***Text inserted or revised in the manuscript:*** “Findings from La Réunion cannot be extrapolated to the continent without significant caution. [...] We reiterate, therefore, that chronic arthropathy figures derived from La Réunion should not be read as continental African estimates.”

***Comment 3.4 (ZIKV lineage).*** *The African ZIKV lineage was not endemic in Cabo Verde. The authors should specify where it was endemic, for example Senegal, a neighbouring country, and note that Faye and colleagues offer a plausible explanation for why the Asian lineage rather than the endemic African lineage was observed in Cabo Verde.*

**Response.** Thank you for the correction, which we have made. We now state that the African lineage is endemic to West Africa, including neighbouring Senegal, that it was not the strain responsible in Cabo Verde, and that the appearance of the Asian lineage there is plausibly explained by an earlier cryptic introduction, citing Faye and colleagues 2020 and 2014. These two references have been added to the reference list as 99 and 100.

***Text inserted or revised in the manuscript:*** “The African ZIKV lineage is endemic to West Africa, including neighbouring Senegal [100], and was not the strain responsible in Cabo Verde; the appearance of the Asian rather than the regionally endemic African lineage in the archipelago is plausibly explained by an earlier cryptic introduction of the Asian lineage [99].”

***Comment 3.5 (adjusting for social determinants).*** *The suggestion to adjust for social determinants at line 445 is not advisable. Adjusting would limit generalisability to real-world contexts, where these factors interact with health outcomes, including genetic factors.*

**Response.** We agree and have changed the wording. The manuscript now calls for social determinants to be measured and characterised alongside genetic data, rather than adjusted for, which is the correct framing for the reasons the reviewer gives.

***Text inserted or revised in the manuscript:*** “Until studies explicitly measure and characterise social determinants alongside genetic data, the relative contribution of biological versus social factors will remain unclear.”

***Comment 3.6 (GRADE in Limitations).*** *The low GRADE ratings should be explicitly acknowledged in the Limitations section.*

**Response.** Added. The Limitations now state the very low certainty explicitly and note that it should temper the weight placed on the pooled values.

***Text inserted or revised in the manuscript:*** “The certainty of evidence for both pooled meta-analytic outcomes was rated very low under the GRADE framework (Supplementary Table S5), and this low certainty is itself a limitation that should temper the weight placed on the pooled values throughout.”

***Comment 3.7 (typographical).*** *Line 30, 7th of March. Line 292, response to RVFV, a finding, requires a comma.*

**Response.** Both corrected. The Abstract date now reads 7th March 2026, and the comma has been added in the RVFV determinants paragraph.

***Text inserted or revised in the manuscript:*** “genetic variation in innate viral sensing pathways fundamentally shapes the host response to RVFV, a finding with direct translational implications for risk stratification during outbreaks.”

***Comment 3.8 (figures).*** *The Egger plot and the map are more interpretable. Consider individual forest plots for each of the five arboviruses to illustrate within-disease heterogeneity and support narrative synthesis.*

**Response.** We are pleased the revised Egger plot and map read more clearly. On the individual forest plots, the per-region subgroup forest plots in Supplementary Figure S1 already display the within-disease dispersion for the two viruses that were pooled, RVFV and DENV. For CHIKV, YFV and ZIKV no pooling was undertaken, since heterogeneity in design and severity definitions precluded it, so a forest plot is not applicable; these are handled entirely by narrative synthesis, with the individual study values given in Table 1. We hope this, combined with the reframing under comments 3.1 and 3.2, meets the intent of the suggestion.

We are grateful to the editor and reviewers for their time, and we hope the revised manuscript now meets the requirements of the journal.

Yours sincerely, Peter Asaga Mac, on behalf of all authors
