# Supplementary material for: Viral and host determinants of arboviral disease severity in African populations: A systematic review with meta-analysis
Source: PLoS Negl Trop Dis. 2026 Aug 20;20(8):e0014663. doi: 10.1371/journal.pntd.0014663 (PMC13515024; doi:10.1371/journal.pntd.0014663)
Supplement: Peer review history

# Peer review: Decision Letter 0

**DOI:** 10.1371/journal.pntd.0014663.r001

**Authors:** Mabel Carabali

29 Apr 2026

Viral and host determinants of arboviral disease severity in African populations: a systematic review with meta-analysis

PLOS Neglected Tropical Diseases

Dear Dr. Asaga,

Thank you for submitting your manuscript to PLOS Neglected Tropical Diseases. After careful consideration, we feel that it has merit but does not fully meet PLOS Neglected Tropical Diseases's publication criteria as it currently stands. Therefore, we invite you to submit a revised version of the manuscript that addresses the points raised during the review process.

Please submit your revised manuscript within by Jun 28 2026 11:59PM. If you will need more time than this to complete your revisions, please reply to this message or contact the journal office at plosntds@plos.org. When you're ready to submit your revision, log on to https://www.editorialmanager.com/pntd/ and select the 'Submissions Needing Revision' folder to locate your manuscript file.

Please include the following items when submitting your revised manuscript:

* A letter that responds to each point raised by the editor and reviewer(s). You should upload this letter as a separate file labeled 'Response to Reviewers'. This file does not need to include responses to any formatting updates and technical items listed in the 'Journal Requirements' section below.

* A marked-up copy of your manuscript that highlights changes made to the original version. You should upload this as a separate file labeled 'Revised Manuscript with Track Changes'.

* An unmarked version of your revised paper without tracked changes. You should upload this as a separate file labeled 'Manuscript'.

If you would like to make changes to your financial disclosure, competing interests statement, or data availability statement, please make these updates within the submission form at the time of resubmission. Guidelines for resubmitting your figure files are available below the reviewer comments at the end of this letter.

We look forward to receiving your revised manuscript.

Kind regards,

Mabel Carabali, M.D., M.Sc., Ph.D.,

Section Editor

PLOS Neglected Tropical Diseases

Mabel Carabali

Section Editor

PLOS Neglected Tropical Diseases

Shaden Kamhawi

co-Editor-in-Chief

PLOS Neglected Tropical Diseases

orcid.org/0000-0003-4304-636XX

Paul Brindley

co-Editor-in-Chief

PLOS Neglected Tropical Diseases

orcid.org/0000-0003-1765-0002

**Additional Editor Comments:**

Thank you for submitting your manuscript. Please provide the responses tp the reviewers below and the attached document.

**Journal Requirements:**

If the reviewer comments include a recommendation to cite specific previously published works, please review and evaluate these publications to determine whether they are relevant and should be cited. There is no requirement to cite these works unless the editor has indicated otherwise.

1) Please ensure that the CRediT author contributions listed for every co-author are completed accurately and in full.

At this stage, the following Authors/Authors require contributions: PETER MAC ASAGA. Please ensure that the full contributions of each author are acknowledged in the "Add/Edit/Remove Authors" section of our submission form.

The list of CRediT author contributions may be found here: https://journals.plos.org/plosntds/s/authorship#loc-author-contributions

2) Your manuscript's sections are not in the correct order.  Please amend to the following order: Abstract, Introduction, Results, Discussion, and Methods

3) Please upload all main figures as separate Figure files in .tif or .eps format. For more information about how to convert and format your figure files please see our guidelines:

https://journals.plos.org/plosntds/s/figures

4) Tables should not be uploaded as individual files. Please remove these files and include the Tables in your manuscript file as editable, cell-based objects. For more information about how to format tables, see our guidelines:

https://journals.plos.org/plosntds/s/tables

5) We have noticed that you have uploaded Supporting Information files, but you have not included a list of legends. Please add a full list of legends for your Supporting Information files after the references list.

6) Please amend your detailed Financial Disclosure statement. This is published with the article. It must therefore be completed in full sentences and contain the exact wording you wish to be published.

1) State the initials, alongside each funding source, of each author to receive each grant. For example: "This work was supported by the National Institutes of Health (####### to AM; ###### to CJ) and the National Science Foundation (###### to AM)."

2) State what role the funders took in the study. If the funders had no role in your study, please state: "The funders had no role in study design, data collection and analysis, decision to publish, or preparation of the manuscript."

3) If any authors received a salary from any of your funders, please state which authors and which funders..

If you did not receive any funding for this study, please simply state: u201cThe authors received no specific funding for this work.u201d

7) As required by our policy on Data Availability, please ensure your manuscript or supplementary information includes the following:

- A numbered table of all studies identified in the literature search, including those that were excluded from the analyses.

- For every excluded study, the table should list the reason(s) for exclusion.

- If any of the included studies are unpublished, include a link (URL) to the primary source or detailed information about how the content can be accessed.

- A table of all data extracted from the primary research sources for the systematic review and/or meta-analysis. The table must include the following information for each study:

- Name of data extractors and date of data extraction

- Confirmation that the study was eligible to be included in the review.

- All data extracted from each study for the reported systematic review and/or meta-analysis that would be needed to replicate your analyses.

- If data or supporting information were obtained from another source (e.g. correspondence with the author of the original research article), please provide the source of data and dates on which the data/information were obtained by your research group.

- If applicable for your analysis, a table showing the completed risk of bias and quality/certainty assessments for each study or outcome.  Please ensure this is provided for each domain or parameter assessed. For example, if you used the Cochrane risk-of-bias tool for randomized trials, provide answers to each of the signalling questions for each study. If you used GRADE to assess certainty of evidence, provide judgements about each of the quality of evidence factor. This should be provided for each outcome.

- An explanation of how missing data were handled.

This information can be included in the main text, supplementary information, or relevant data repository. Please note that providing these underlying data is a requirement for publication in this journal, and if these data are not provided your manuscript might be rejected.

8) Some material included in your submission may be copyrighted. According to PLOS's copyright policy, authors who use figures or other material (e.g., graphics, clipart, maps) from another author or copyright holder must demonstrate or obtain permission to publish this material under the Creative Commons Attribution 4.0 International (CC BY 4.0) License used by PLOS journals. Please closely review the details of PLOS's copyright requirements here: PLOS Licenses and Copyright. If you need to request permissions from a copyright holder, you may use the PLOS Content Copyright Permission Form Please respond directly to this email and provide any known details concerning your material's license terms and permissions required for reuse, even if you have not yet obtained copyright permissions or are unsure of your material's copyright compatibility. Once you have responded and addressed all other outstanding technical requirements, you may resubmit your manuscript within Editorial Manager. Potential Copyright Issues:

i) Figure 6. Please (a) provide a direct link to the base layer of the map (i.e., the country or region border shape) and ensure this is also included in the figure legend; and (b) provide a link to the terms of use / license information for the base layer image or shapefile. We cannot publish proprietary or copyrighted maps (e.g. Google Maps, Mapquest) and the terms of use for your map base layer must be compatible with our CC BY 4.0 license. Note: if you created the map in a software program like R or ArcGIS, please locate and indicate the source of the basemap shapefile onto which data has been plotted. If your map was obtained from a copyrighted source please amend the figure so that the base map used is from an openly available source. Alternatively, please provide explicit written permission from the copyright holder granting you the right to publish the material under our CC BY 4.0 license. If you are unsure whether you can use a map or not, please do reach out and we will be able to help you. The following websites are good examples of where you can source open access or public domain maps: * U.S. Geological Survey (USGS) - All maps are in the public domain. (http://www.usgs.gov) * PlaniGlobe - All maps are published under a Creative Commons license so please cite u201cPlaniGlobe, http://www.planiglobe.com, CC BY 2.0u201d in the image credit after the caption. (http://www.planiglobe.com/?lang=enl) * Natural Earth - All maps are public domain. (http://www.naturalearthdata.com/about/terms-of-use/).

9) Please ensure that the files are uploaded in the online submission form in a correct numerical order.

10) Please ensure that captions are included for all six figures in your manuscript.

11) Kindly amend your competing statement in the online submission form to align with the journal's style guidelines: 'The authors declare that there are no competing interests.'

**Reviewers' Comments:**

Reviewer's Responses to Questions

**Key Review Criteria Required for Acceptance?**

As you describe the new analyses required for acceptance, please consider the following:

**Methods**

-Are the objectives of the study clearly articulated with a clear testable hypothesis stated?

-Is the study design appropriate to address the stated objectives?

-Is the population clearly described and appropriate for the hypothesis being tested?

-Is the sample size sufficient to ensure adequate power to address the hypothesis being tested?

-Were correct statistical analysis used to support conclusions?

-Are there concerns about ethical or regulatory requirements being met?

Reviewer #1: Methods

Are the objectives of the study clearly articulated with a clear testable hypothesis stated?

Partially. The manuscript addresses a relevant and important research question; however, the specific objectives, the explicit research purpose, and the precise question guiding the study should be articulated more clearly in the Introduction. Greater precision is needed to fully meet the standards.

Is the study design appropriate to address the stated objectives?

Yes, in principle. The manuscript follows the standard methodological framework of a systematic review with meta-analysis, including study selection, appraisal, data extraction, and synthesis. Nevertheless, key methodological choices—particularly in the data synthesis section—require clearer justification and more detailed explanation.

Is the population clearly described and appropriate for the hypothesis being tested?

Partially. The focus on African populations is appropriate and represents a clear strength of the study. However, additional epidemiological contextualization and clearer characterization of the included populations would improve interpretability and strengthen the manuscript.

Is the sample size sufficient to ensure adequate power to address the hypothesis being tested?

Not fully established. While the number of included studies is substantial, the evidentiary base is uneven across arboviruses, and high heterogeneity limits the strength of some inferences. The near-absence of data for certain viruses (e.g., ZIKV) further constrains the robustness of conclusions.

Were correct statistical analyses used to support conclusions?

Partially. The analytical approaches are generally appropriate; however, they are not yet sufficiently justified. All statistical procedures should be clearly referenced and explained. In particular, the rationale for selecting the Freeman–Tukey double arcsine transformation over alternative approaches (e.g., GLMMs) should be explicitly stated. Additional detail on the use of computational tools (Python, NumPy, SciPy) is also required. Providing the analytical code or syntax would substantially strengthen transparency and reproducibility.

Are there concerns about ethical or regulatory requirements being met?

No major concerns identified. The review does not raise specific issues regarding ethical or regulatory compliance.

Reviewer #2: Structured Population, exposure, comparison, outcome and eligible study designs need to be clearly defined in organized form rather than having to be rebuilt on prose. This is one of the basic requirements of reproducibility.

Search Strategy - It should include Four databases and grey literature, which are incomplete. Nevertheless, there are no Boolean operators, MeSH terms, or database syntax included in the search string. In its absence, the review is not reproducible - which is the point of systematic review methodology. A complete search plan should be given as an additional appendix.

Eligibility Criteria is only partially justified. The ≥5 number of patients as a case series inclusion criterion is not justified. Small case series add heterogeneity at a disproportionate rate to its epidemiological signal. Either increase the threshold or explain it.

Screening Process: The kappa or percentage agreement as reported by Cohen at the two stages is not mentioned. This omission is not insignificant in the case of 297 screened records and 191 complete texts.

Data Extraction Instrument was not stated. The 39-field extraction form is not reproduced or given as a supplement, but is described. Readers are unable to determine consistent application of heterogeneous study designs.

Quality Assessment: NOS Applied but GRADE Absent The Newcastle-Ottawa Scale is implemented in cohort, case-control, and cross-sectional designs, although the particular adaptations applied to each design are not mentioned. What is more important, GRADE evaluation of the overall certainty of evidence is not present at all. The authors directly recommend policy on the use of vaccines, host directed therapy and pregnancy registries based on what they describe as an evidence base that is mainly of moderate quality and cross sectional. GRADE was created specifically to fill this gap. The most significant methodological lack of the manuscript is its absence.

Statistical Methods are defensible and in order.

Reviewer #3: Given the very high I², I am concerned about misinterpretation of the pooled results. Caution should be flagged early and consistently e.g. immediately after presenting I² results: "...this average should be interpreted with significant caution as it masks substantial clinical and methodological diversity…" rather than only at the end. The authors used Freeman-Tukey double arcsine transformation in meta-analysis, however, I am concerned about how the results of the meta-analysis could be interpreted, especially as the DerSimonian-Laird can underestimate between-study variance ( 2) when heterogeneity is high. Recent literature has advised caution about the interpretability of the Freeman-Tukey method (https://doi.org/10.1002/jrsm.1348 , https://doi.org/10.1002/hsr2.178). Generalized Linear Mixed Models (GLMMs) have been recommended in place; the between studies variance can be accounted for in the same model.

I strongly recommend presenting the forest plots without pooling (severe dengue proportions in Africa; RVFV case fatality rate). Replacing this, as they did for YFV, CHIKV, and ZIKV with a narrative synthesis and I² reported below each plot (without the pooled estimate) would push readers to engage with the variation across studies rather than to hang on to a spurious pooled number. This visual display could be applied across all five arboviruses to further emphasise the current heterogeneity in the landscape.

Where Egger's test already flags significant publication bias, describing estimates as "indicative rather than definitive" is not, in my opinion, a sufficient safeguard; removing the pooled estimate would be a much more defensible choice, especially as the authors reiterate these cautions about heterogeneity and interpretability throughout the manuscript. Otherwise, the authors present a genuinely valuable synthesis with this systematic review.

**********

**Results**

-Does the analysis presented match the analysis plan?

-Are the results clearly and completely presented?

-Are the figures (Tables, Images) of sufficient quality for clarity?

Reviewer #1: Results

Does the analysis presented match the analysis plan?

Partially. The manuscript follows a coherent analytical framework consistent with a systematic review and meta-analysis; however, the linkage between the stated analytical approach and its implementation is not always fully transparent. Greater clarity is needed in the Methods–Results alignment, particularly regarding the justification and application of specific statistical procedures (e.g., transformation methods and heterogeneity analyses).

Are the results clearly and completely presented?

Partially. The Results section contains valuable and relevant findings, and it represents one of the strongest components of the manuscript. However, its internal organization lacks consistency across the five arboviruses. The analytical structure is not applied uniformly (e.g., dengue is presented using distinct categories, whereas other viruses are not), which affects clarity and comparability. A more standardized and parallel presentation framework across all arboviruses is recommended to improve coherence and interpretability.

Are the figures (Tables, Images) of sufficient quality for clarity?

Partially. While tables (e.g., summary tables of determinants) are informative and contribute positively to the manuscript, several issues require attention. Figure numbering appears inconsistent and should be systematically verified. In addition, figure readability is limited by small font sizes, and overall presentation does not yet fully meet PLOS NTD standards. The current visualization of study locations would benefit from improvement, for example through a higher-quality georeferenced map. Revisions are needed to enhance clarity, consistency, and publication quality.

Reviewer #2: Numerical Inconsistencies exist in the result. This is a critical point of the result and unacceptable. There are numerical inconsistencies between the abstract, results text and figure legends of both meta-analyses.

For RVFV, the abstract and results text report a pooled CFR of 16.9% (95% CI 11.6–22.8); Figure 2 legend and funnel plot discussion cite 17.4% (95% CI 11.8–23.6). These do not represent differences in rounding.

For DENV: abstract and results text state 10 African studies, pooled estimate 8.5% (95% CI 3.0–16.2; I² = 95.7%, Q = 210.3); Figure 3 legend states 12 studies, pooled estimate 11.1% (95% CI 5.1–18.7; I² = 95.0%, Q = 198.7). These are figures that are substantively different between the same manuscript and are a quality control failure that needs to be addressed by providing the underlying analysis scripts.

There are areas of uneven depth of RVFV and DENV that are substantive and well-referenced. CHIKV is overly reliant on La Reunion data - a French overseas territory whose health system is not representative of continental Africa. The one study on CHIKV in continental Africa (Kassala, Sudan) has one sentence. This representativeness issue, not an issue of data constraint, must be foregrounded and not described in limitations in a few lines.

ZIKV is handled appropriately given the evidence vacuum. Nevertheless, the use of mouse-model pathogenicity data by Raulino should be marked clearly as evidence of animal experimentation. Non-experts who read the primary literature might confuse experimental findings of transmissibility with clinical burden of humans.

Cross-Cutting Sections: There are certain assertions made without structural backing. The observation that 15 to 40% of patients with malaria in studies in Nigeria and Cameroon contained concomitant arboviral infections is startling and has clinical implications. It is a narrative claim as it is put. It involves systematic synthesis of specifying the studies, the arboviruses, the denominator populations and the diagnostic methods used to obtain that number.

PRISMA Compliance hasnt been filed. No reference is made to PRISMA 2020 checklist as having been filled in and submitted as a supplement. It should be.

Reviewer #3: Citations 14 and 15 appear to share the same DOI in the references. When accessing García et al. (doi:10.4269/ajtmh.2010.09-0353), I noted a sample size of only 67 females and 30 males -- far too small to draw causal inferences or to generalise to African populations outside of Cuba. I strongly recommend flagging this in the discussion. This sample size is underpowered. Findings may serve as candidate gene hypotheses for further study (as the authors suggest in reference to Sierra B et al., which had a substantially larger sample) but should not be treated as reliable evidence, especially given the additional consideration of gene-environment interactions and social dynamics that may be at play. Indeed, this is precisely the kind of preliminary signal that justifies calls for large-scale Genome-Wide Association Studies (GWAS) to detect common diseases with small effects.

Similar caution should be brought to the interpretation of the results and discussion of the paper by Boillat-Blanco N et al. (https://doi.org/10.1186/s12879-018-3549-z). The original authors did not elucidate how race was determined, presenting race entirely as a biological construct, and excluded mixed-race individuals from their analysis (by undisclosed criteria). The authors of this manuscript themselves note that all patients were infected with the same DENV-2 genotype, yet that that “black race was independently and significantly protective against severe dengue” was purported by Boillat-Blanco N et al. Results from this article should be interpreted with great caution and on an exploratory basis only; causal inferences are not warranted, despite its more direct geographic / contextual grounding.

**********

**Conclusions**

-Are the conclusions supported by the data presented?

-Are the limitations of analysis clearly described?

-Do the authors discuss how these data can be helpful to advance our understanding of the topic under study?

-Is public health relevance addressed?

Reviewer #1: Conclusions

Are the conclusions supported by the data presented?

Yes, with minor qualifications. The conclusions are generally consistent with the data presented and reflect the main findings of the review, particularly regarding immunopathological patterns, genetic considerations, and identified evidence gaps. However, given the substantial heterogeneity reported and uneven evidence across arboviruses, some interpretations would benefit from more cautious framing to avoid potential overgeneralization.

Are the limitations of analysis clearly described?

Partially. The manuscript acknowledges several important limitations, including gaps in the evidence base (e.g., limited data for certain viruses such as ZIKV). However, the discussion of limitations could be strengthened by more explicitly addressing issues related to heterogeneity, comparability across regions, methodological constraints, and variability in study designs and diagnostic criteria.

Do the authors discuss how these data can be helpful to advance our understanding of the topic under study?

Yes. The Discussion and Conclusions effectively highlight the contribution of the study to advancing understanding of arboviral disease severity in African populations. In particular, the identification of cross-cutting immunopathological mechanisms and the integration of viral, host immune, and genetic determinants represent valuable contributions to the field.

Is public health relevance addressed?

Yes. The manuscript clearly articulates the public health implications of the findings, including their relevance for vaccine deployment, clinical management, and preparedness strategies. This aligns well with the scope and expectations of PLOS Neglected Tropical Diseases.

Reviewer #2: The conclusions are supported but with few observations. The most strongly supported finding of cytokine convergence is between fatal RVFV and YFV, based on several independent cohorts that have a consistent directional finding in a variety of outbreak settings and using a variety of different laboratory techniques.

The genetic dengue protection argument is based on three indirect lines of evidence: a Tanzanian clinical observation that race was a protective factor in a single outbreak cohort, a Cuban admixture mapping study that identified OSBPL10 and RXRA and a Cuban Fc17RIIa polymorphism study. All of them are not a direct GWAS in a cohort of continental African with severe dengue as an outcome. This is the body of evidence that is hypothesis-generating and not confirmation. This difference is not well represented in the conclusions section and needs to be reworded to describe the genetic protection finding specifically as preliminary and in need of direct continental African confirmation.

The finding of CHIKV chronicity is clinically consistent but is largely based on La Réunion data, as mentioned above.

GRADE Absent - Policy Implications Unsupported The implications section presents explicit policy recommendations - recalibrating dengue vaccine risk-benefit calculations to Africa, initiating host-directed cytokine therapy trials, creating ZIKV pregnancy registries. These are practical suggestions to health decision-makers. In the absence of GRADE ratings on top of the underlying evidence, readers cannot determine whether the evidence base underpins strong or conditional recommendations. This is not merely a scholarly nicety but a patient safety concern when systematic reviews guide clinical and vaccine policy.

There are two gaps observed in the limitation section. The honesty of publication bias in RVFV, diagnostic heterogeneity, La Reunion representativeness and concentration of cross-sectional designs are all recognized. There are two other limitations that ought to be clearly identified, the first is that since no individual patient data are available cross-study confounder adjustment cannot be performed, that is there is no way to adjust the observed severity differences across populations and this should be observed as a limitation instead of a caveat in the discussion section. The second limitation is that since no individual patient data are available, cross-study confounder adjustment cannot be done.

The work has public health relevance. The climate change and the expansion of the range of vectors paragraph in the conclusion appears as a generic appended statement, not an argument that integrates evidence. Unless these factors do not apply to the findings of the review, they should be related to certain epidemiological projections or the included studies.

Reviewer #3: Overall, this is a much-needed synthesis that raises many relevant public health findings and reminds us how many questions remain unanswered about arboviruses and their determinants; viral, immunological, genetic, and social, and at the intersection of fields beyond descriptive epidemiology (genomics, virology, immunology). While the authors address several limitations including heterogeneity, they still present pooled estimates from the meta-analysis. I strongly advise against this.

**********

**Editorial and Data Presentation Modifications?**

Use this section for editorial suggestions as well as relatively minor modifications of existing data that would enhance clarity. If the only modifications needed are minor and/or editorial, you may wish to recommend “Minor Revision” or “Accept”.

Reviewer #1: Editorial and Data Presentation Modifications

Several editorial and data presentation improvements are recommended to enhance clarity, consistency, and compliance with PLOS Neglected Tropical Diseases standards.

First, manuscript formatting should be corrected to include consecutive page and line numbering, which is essential for an efficient peer review process.

Second, language, grammar, and syntax should be carefully revised throughout the manuscript. While most issues are minor, their cumulative effect does not yet meet the standard expected for a Q1 publication. Professional or AI-assisted language editing may be beneficial.

Third, typographical and notation inconsistencies should be corrected. For example, statistical notation (e.g., “I²” vs. “I2”) should be standardized across the manuscript.

Fourth, the Results section requires improved structural consistency. A more uniform analytical framework across all five arboviruses should be implemented to enhance comparability and readability.

Fifth, figures and tables require revision. Figure numbering should be systematically checked for consistency with in-text citations. In addition, figure readability should be improved by increasing font sizes and aligning presentation with PLOS NTD standards. The current visualization of study locations would benefit from enhancement, for example through a higher-quality georeferenced map.

Sixth, the statistical reporting requires improved referencing and clarity. All statistical procedures (e.g., Egger’s test, transformation methods) should be properly cited, and interpretive criteria should be clearly specified.

Seventh, the Methods section would benefit from greater transparency in data analysis, including a clearer description of the use of computational tools (Python, NumPy, SciPy). Providing the analytical code or syntax as supplementary material is strongly recommended to improve reproducibility.

Eighth, the reference list requires substantial editorial revision to comply with Vancouver style. Missing bibliographic elements, inconsistent use of “et al.”, incorrect capitalization, duplicate entries, and absent DOIs should be systematically corrected.

Overall assessment: While many of these issues are editorial in nature, they are numerous and collectively substantial. Therefore, these modifications go beyond minor revision and support the recommendation of major revision rather than acceptance in the current form.

Reviewer #2: Should Revise Before Resubmission.

The repeated words, taken together, taken together (Page 18; Line 17), in the DENV host immune section should be removed. It is a simple error of proofreading in a peer review manuscript.

In Methodology

Add PRISMA checklist citation and check the presence of PRISMA flow diagram as a supplementary or primary figure.

The explanation why Cuban studies should be included is correct but needs to be relocated to a special sentence under the heading Rationale why non-African populations should be included.

Tables 1 and 2 have been mentioned in the entire context of the manuscript but were not completely copied in the text that was submitted. Their accuracy and fullness cannot be evaluated. They should be both given in detail.

It is mentioned in the manuscript that the PROSPERO registration took place prior to data extraction, however, it is not made clear whether it took place prior to the literature search itself - PROSPERO stipulates prospective registration prior to both. This should be clarified unambiguously.

The entire search plan should be included in a separate appendix – Boolean operators, MeSH terms and database-specific field tags of all four databases.

The PRISMA 2020 checklist should be filled in and sent as the supplementary document.

The data extraction tool (39-field form) should be attached as an additional appendix.

The abstract, text, and figure legends contain numerical discrepancies in both meta-analyses, which need to be addressed by re-running and resubmitting the analysis, with uniform reporting across abstract, text and figure legends. The Python scripts underlying should be provided as a supplement.

Reviewer #3: Introduction:

Perhaps a comma is missing here “but the cross-cutting themes [,] the role of African genetic ancestry,”

Methods Search Strategy:

In the search strategy, some words have quotes in them and others do not? Please make it consistent.

Methods Eligibility Criteria: may be a comma missing here “... that specifically identified African-ancestry alleles conferring protection against dengue severity [,] these were judged directly relevant …”

Methods Data Synthesis: “... specifically, studies reporting the number of severe or fatal cases among a defined denominator of confirmed infections [,] random-effects meta-analysis of proportions was performed using the DerSimonian–Laird method with Freeman–Tukey double arcsine transformation to stabilise variances, …”

Results Study Selection and Characteristics: Figure 1: I recommend removing the last box under the number of studies included in the meta analysis. Since it is referring to the 155 papers, authors could put this in the “Studies included in the qualitative synthesis” box, disaggregating the numbers, and do a similar disaggregation for the studies included in the meta analysis: RVFV / DENV (though upon reading the manuscript in its entirety, I do not recommend conducting a meta-analysis).

Typo to be corrected in the Dengue virus viral determinants portion: “Taken together, taken together, [sic] these data are consistent with the hypothesis that the apparently milder dengue phenotype”

Please in Figure 4A present the funnel plot with the 95% confidence interval diagonal lines for better visualisation of the asymmetry. Until further homogenous studies are conducted/ made available, I do not suggest presenting pooled estimates.

Other suggestions: Create a figure / box with the studies included in the systematic analysis, sorted by disease. Include the year of publication, region, title etc. for a tabular representation of said papers.

**********

**Summary and General Comments**

Use this section to provide overall comments, discuss strengths/weaknesses of the study, novelty, significance, general execution and scholarship. You may also include additional comments for the author, including concerns about dual publication, research ethics, or publication ethics. If requesting major revision, please articulate the new experiments that are needed.

Reviewer #1: Summary and General Comments

This manuscript follows the standard methodological architecture expected of a systematic review with meta-analysis, including problem formulation, systematic literature searching, study selection and appraisal, data extraction, statistical synthesis, interpretation, and reporting. The study is well aligned with the scope of PLOS Neglected Tropical Diseases and addresses an important gap in the literature by examining viral and host determinants of disease severity across five arboviruses in African populations.

A key strength of the manuscript lies in its comparative and integrative perspective, which highlights potentially cross-cutting mechanisms, including the role of African genetic ancestry, flavivirus cross-reactive immunity, and shared inflammatory pathways underlying severe disease. The Results, Discussion, and Conclusions are particularly strong and provide a meaningful contribution to the field, especially in relation to clinical severity patterns and immunopathological mechanisms.

At the same time, several important issues require attention before the manuscript can be considered for publication. These include aspects of contextualization, methodological transparency, analytical justification, structural consistency, and editorial presentation.

The Introduction would benefit from stronger epidemiological contextualization and a more explicit articulation of the study’s specific contribution and research question. The Methods section requires clearer justification and referencing of statistical procedures, particularly regarding the choice of transformation methods, as well as improved transparency in the use of computational tools. Providing analytical code or syntax would substantially strengthen reproducibility.

The Results section, while rich and informative, would benefit from a more consistent and parallel organizational structure across the five arboviruses, to enhance clarity and comparability. In addition, figure presentation, numbering, and readability should be improved to meet journal standards.

The Discussion is one of the strongest sections of the manuscript; however, minor language and syntactic issues remain and should be corrected. The reference list requires systematic revision to comply with Vancouver style, including completion of missing bibliographic elements and correction of formatting inconsistencies.

No major concerns regarding research ethics or dual publication are identified.

Importantly, this is a secondary data study, and therefore no new experiments are required. However, several analytical and reporting improvements are necessary for acceptance. These include:

-Clearer articulation of the research objectives and contribution

-Explicit justification and referencing of all statistical methods

-Improved transparency and reproducibility (including provision of code/syntax)

-More consistent structuring of the Results across arboviruses

-Strengthened epidemiological contextualization and comparative framing

In its current form, the manuscript has clear potential but does not yet meet the standards required for publication in a Q1 journal. A major revision is therefore recommended to fully realize its scientific contribution.

Reviewer #2: Overall Assessment

The evidence gap that is being discussed in this review is really important. None of the five major arboviruses have had severity determinants synthesised in African populations in a single comparative framework. Cross-cutting themes (common terminal cytokine pathways, the genetic dengue protection hypothesis and the vacuum of ZIKV evidence) are scientifically relevant and directly relevant to policy. The aspiration of the review is correct, and the populace health justification is highly inspired.

Strengths

The grey literature inclusion multi-database search is complete. The choice to provide evidence of genetic dengue protection using African-diaspora Cuban cohorts but to present them as comparator data as opposed to presenting them as primary African data is carefully methodological and well-managed. The strongest part of the review is the RVFV host determinant synthesis, which combines the cytokine profiling, the viral load quantification, the innate immune genetic data, well-referenced narrative with direct translational implications in the form of the risk stratification of the outbreaks. The openness of the ZIKV evidence gap is correct and the experimental African lineage pathogenicity data obviously caveated as animal-derived, establishing a valid justification of the urgency of the research. The limitations section affirms scholarly honesty that is not ubiquitous in this literature and portrays the authors at their finest.

Weaknesses

There are three areas of weakness that are in need of large-scale revision. To begin with, the meta-analyses of the numerical inconsistencies between abstract and results text and figure legends of both meta-analyses are not admissible in the submission to a peer-reviewed journal and reflect the lack of internal quality control. Second, the DENV meta-analysis with I 2 = 95.7% does not provide any reliable pooled estimate; it cannot serve as a direct comparator with Asian cohort statistics without further flaunt in the entire text (not just in the limitations section). Third, the lack of GRADE assessment makes the section of policy implications evidence-based clear. On the basis of mainly moderate-quality cross-sectional evidence, the authors present specific actionable recommendations on the topic of vaccine deployment and clinical management without giving health decision-makers any official certainty score. This is the only one most important revision that is needed.

Novelty

Confirmed. The African population multi-arbovirus comparative framework is new. The convergence of cytokines between RVFV and YFV, which, upon confirmation in the future, is a real therapeutic result. The synthesis of evidence of genetic protection against dengue offers a consistent platform of a continental African GWAS that is not yet undertaken.

Execution

Uneven. The RVFV and DENV parts are substantive. The CHIKV section is underweight in comparison with its relevance in the continent of Africa. The ZIKV section is also reduced. The cross-cutting segments locate the correct questions but in the form of narrative statements instead of the synthesis.

Reviewer #3: The authors presented a strong rationale for systematic review with clear thought put into anticipating and quantifying heterogeneity. Interesting and relevant findings were raised across all five arboviruses, notably: findings that could inform risk stratification in future RVFV outbreaks; the higher pathogenicity and foetal transmissibility of the African vs. Asian ZIKV strain (preliminary, in mice); and the need for longitudinal studies on CHIKV severity in African countries with recent outbreaks, including comparisons with Afro-descendant populations in La Réunion (which has different infrastructural and social conditions).

While the focus is on immunological and genetic determinants, social dynamics likely influence the differences observed, including in the comparative populations with African ancestry outside of Africa, and warrant consideration. The regions where Black race appeared protective; Tanzania (Dar es Salaam) and Cuba, are demographically heterogeneous, and social determinants may be driving this perceived protection as much as biological ones. This matters particularly because social dynamics can influence gene expression and epigenetic modifications (gene-environment interactions). I would also caution against treating "Black race" as a biological category; it is a social and political construction.

There is greater genetic diversity within populations than between them, which emphasises the need for further research conducted in African populations on the continent to properly elucidate this severity paradox.

For RVFV, the authors note that host response was a more significant determinant of outcome than genetic variation, and this arbovirus had comparatively more contextually grounded data in the population of interest. Yet heterogeneity remained high. If that is the case for the most data-rich arbovirus here, the concern is only greater for the others. Until more homogeneous studies are available, I do not recommend presenting pooled estimates.

**********

PLOS authors have the option to publish the peer review history of their article (what does this mean?). If published, this will include your full peer review and any attached files.

If you choose “no”, your identity will remain anonymous but your review may still be made public.

**Do you want your identity to be public for this peer review?**  For information about this choice, including consent withdrawal, please see our Privacy Policy.

Reviewer #1: No

Reviewer #2: **Yes:**  Nnamso W. Ikpeida

Reviewer #3: No

[NOTE: If reviewer comments were submitted as an attachment file, they will be attached to this email and accessible via the submission site. Please log into your account, locate the manuscript record, and check for the action link "View Attachments". If this link does not appear, there are no attachment files.]

**Figure resubmission:**

**Reproducibility:**

To enhance the reproducibility of your results, we recommend that authors of applicable studies deposit laboratory protocols in protocols.io, where a protocol can be assigned its own identifier (DOI) such that it can be cited independently in the future. Additionally, PLOS ONE offers an option to publish peer-reviewed clinical study protocols. Read more information on sharing protocols at https://plos.org/protocols?utm_medium=editorial-email&utm_source=authorletters&utm_campaign=protocols

Attachment Submitted filename: PNTD-D-26-00516_report.pdf

# Author comment: Author response to Decision Letter 1

**DOI:** 10.1371/journal.pntd.0014663.r002

4 May 2026

Attachment Submitted filename: Response_to_Reviewers and Editors comments.docx

# Peer review: Decision Letter 1

**DOI:** 10.1371/journal.pntd.0014663.r003

**Authors:** Sujatha Sunil

21 Jul 2026

Response to Reviewers Revised Manuscript with Track Changes Manuscript

Shaden Kamhawi

co-Editor-in-Chief

PLOS Neglected Tropical Diseases

orcid.org/0000-0003-4304-636XX

Paul Brindley

co-Editor-in-Chief

PLOS Neglected Tropical Diseases

orcid.org/0000-0003-1765-0002

**Additional Editor Comments (if provided):** **Journal Requirements:**

**Reviewers' comments:**

**Key Review Criteria Required for Acceptance?**

As you describe the new analyses required for acceptance, please consider the following:

**Methods**

-Are the objectives of the study clearly articulated with a clear testable hypothesis stated?

-Is the study design appropriate to address the stated objectives?

-Is the population clearly described and appropriate for the hypothesis being tested?

-Is the sample size sufficient to ensure adequate power to address the hypothesis being tested?

-Were correct statistical analysis used to support conclusions?

-Are there concerns about ethical or regulatory requirements being met?

Reviewer #1: The manuscript has been substantially revised in response to the concerns raised in my previous review. The study objectives are clearly articulated, and the research question is presented explicitly. Given the study's descriptive and systematic review/meta-analysis design, the absence of a formal hypothesis-testing framework is appropriate and does not detract from the scientific objectives.

The study design is appropriate for addressing the stated objectives. The eligibility criteria, literature search strategy, study selection procedures, and methodological framework are clearly described and adhere to established reporting standards. The population included in the review is adequately defined and appropriate for the research question.

The number of included studies and participants provides an adequate evidence base for the analyses conducted. The authors strengthened the methodological description by providing additional justification for the statistical methods employed. This includes the rationale for selecting the Freeman–Tukey transformation and a discussion of alternative approaches. They also appropriately interpreted heterogeneity and publication bias. Including analytical code and supplementary methodological materials enhances the study's transparency and reproducibility.

I do not identify any remaining major methodological or statistical concerns that require additional analyses or experiments. Similarly, I have no concerns about the study's ethical or regulatory compliance because it is based on published literature and follows accepted standards for systematic reviews and meta-analyses.

No new analyses or experiments are required before publication. The remaining observations are limited to minor editorial refinements, including final verification of reference formatting, consistency of the supplementary materials, language editing, and improving figure readability.

Reviewer #2: The objectives of the study are clearly stated in the Introduction that ends with an explicit, well-framed research question: What viral, host immune and host genetic factors have been identified as determinants of clinical severity of DENV, CHIKV, YFV, RVFV and ZIKV infections in African and African-ancestry populations and where are the most consequential gaps in evidence? This is suitably designed as a systematic review question, but not as a hypothesis-based experimental question, which is methodologically appropriate to this type of study.

The Methods section makes it clear that the PECO framework (Population, Exposure, Comparison, Outcome) is applied, with the following points being discussed: population (humans infected in African or African-ancestry populations), exposure (natural infection with one of the five arboviruses), comparison (between severity strata), and outcome (clinical severity and viral/host immune/genetic determinants). This is an ideal degree of transparency of a systematic review.

The two-fold aim of the review, synthesising severity determinants, and evidence gaps mapping are well stated. The reason behind the review is convincing: the overwhelming mechanistic arboviral literature of South-East Asia and Latin America, and the long-standing paradox of seemingly milder dengue in African contexts despite the co-circulation of serotypes. The element of testable hypothesis, however, when taken literally, is implied, as opposed to being stated as a directional hypothesis. As an example, the implicit mechanistic thesis is "African genetic ancestry is hypothesised to partially guard against severe dengue via lipid metabolism and Fc receptor pathways" but it is not presented as a formal hypothesis. To a systematic review, this is fine - the formulation of the question is adequate - but a clear statement of the main mechanistic proposition as a hypothesis would help to focus the Introduction.

A meta-analysis systematic review is quite suitable. A narrative-plus-quantitative synthesis approach is the appropriate methodological choice, based on the geographic distribution of evidence, the variety of arboviruses involved, and the variety of study designs in the primary literature. Both are verified with the current best practices of systematic reviews, the PRISMA 2020 framework with PROSPERO registration. The choice to only consider formal meta-analysis of RVFV case fatality and DENV severe disease proportion, where there was enough homogeneous data to quantify pooling, and synthesise CHIKV, YFV, and ZIKV narratively, is methodologically sound and well-grounded. The use of the Cuban admixture studies as comparator data (not explicitly included in Africa-only primary analyses) is both mechanistically justified and is rightly identified as a design choice that needs to be disclosed. The case series inclusion criterion of ≥5 patients is sensible and is appropriately consistent with precedent (Baudin et al. 2020; Bettis et al. 2022).

There is a well-characterised population. The PECO describes it as infected humans in African populations or populations of known African ancestry. The Methods section is a discussion of the Cuban diaspora exception with a methodological rationale. It is represented at country-level (38 countries; Nigeria n=17, Kenya n=14, Sudan n=14, Ethiopia n=12) which gives it a clear geographic presence. The fact that some countries predominate in the field indicates the publication trends in the area and this is understandable as opposed to a flaw in the sampling. The fact that African-diaspora groups (Cuban cohorts) are included in a reasonable design choice, which is well defined, and rightly omitted in main African meta-analyses is understandable.

One population definition issue: the inclusion criteria include populations of documented African ancestry to be included in the diaspora, but the Cuban cohort studies employed self-reported racial categories and admixture mapping - different methodologies with varying degrees of genetic accuracy. The authors do this quite well in the Discussion but would benefit the Methods by making the eligibility criterion more specific, i.e. by stating that the African ancestry should be determined by genetic admixture mapping or similar methodology rather than by self-report.

The 155 studies included in the review level of 38 countries in a 48-year period of publication (19782026) is a complete and well-powered evidence base to conduct a systematic review. The statistics of the inter-rater agreement (κ = 0.82 at title/abstract; κ = 0.91 at full-text) are great and demonstrate a strong and reproducible screening procedure. In the RVFV meta-analysis (19 studies) and DENV meta-analysis (10 African studies), the absolute number of studies is not large - especially not in the case of DENV - but is listed as such. It is not a lack of studies but rather the extreme between-study heterogeneity (I 2 = 88.4% in the case of RVFV; I 2 = 95.7% in the case of DENV) that is the root cause of limiting the interpretability of any pooled estimate despite the number of studies that contribute to it. The authors recognize this and do so throughout the manuscript and this is the right way to do it.

The statistical approach is usually suitable and well outlined. The proportion meta-analysis using Freeman-Tukey double arcsine transformation is technically correct and justified

An aggregate data systematic review based on published data does not entail any primary ethical approval, and none is alleged or required. Registration at PROSPERO (CRD420261334251, registered 7 March 2026, before the literature search was initiated 8 March 2026 - the one-day gap is technically in compliance with the prospective registration requirements).

The authors please ensure that this PROSPERO number is correctly written. In case of errors, it should be corrected immediately.

Reviewer #3: While the authors chose to retain random effects meta-analysis using Freeman-Tukey double arcsine transformation (presented with additional rationale), the pooled results for dengue severity and RVFV are still concerning due to the heterogeneity of papers in both analyses.

While I appreciate that the authors have addressed heterogeneity throughout the manuscript, the extent of these caveats itself underscores the problem: if pooled estimates require this degree of cautionary framing to avoid misinterpretation, their presence in the main text is difficult to justify. The presentation of these numbers still invite selective citation regardless of the cautions. I therefore recommend moving the pooled results to supplementary materials, where they remain available for transparency, and focusing the main text on a narrative synthesis of individual study findings.

**********

**Results**

-Does the analysis presented match the analysis plan?

-Are the results clearly and completely presented?

-Are the figures (Tables, Images) of sufficient quality for clarity?

Reviewer #1: The analyses presented are consistent with the study objectives and the authors' described methodological approach. The revised manuscript shows greater consistency between the analysis plan, statistical methods, and reported findings. The presentation of the pooled estimates, heterogeneity assessments, subgroup analyses, and publication bias evaluations is appropriate, supporting the conclusions drawn.

The results section has been substantially reorganized and is now clearer and more coherent. The authors strengthened their interpretation of the findings by acknowledging the high between-study heterogeneity and avoiding overinterpretation of the pooled estimates. These revisions have also improved the Results section's overall readability and logical flow.

The tables and figures adequately support the presentation of the results and are generally of good quality. The revised graphical presentation is an improvement over the original submission. As a minor editorial recommendation, I encourage the authors to ensure that all figures are legible in the final production version, including font size, axis labels, legends, and image resolution.

Overall, I do not identify any additional analyses or experiments necessary before publication. The remaining observations are limited to minor editorial and presentation refinements.

Reviewer #2: All of the analyses reported are in good agreement with the analysis methods described. As described in the Methods section, data synthesis, the narrative synthesis is organized by virus (DENV, RVFV, CHIKV, YFV, ZIKV), with an additional substructure for clinical severity, viral determinants and host immune/genetic determinants separately.

The importance of providing well-structured results with a clear writing style overall is recognized. The DENV section is the best detailed and most analytically complex, giving (arithmetically) a pooled severe disease proportion of ranging 8.5%, 95% CI 3.0–16.2, with the caveat of extreme heterogeneity (I² = 95.7%).

The Figures can be accessed from the editorial management system as external links. The forest plots (which are references in Figure 2 and 3), funnel plot (reference in Figure 4), PRISMA (reference in Figure 1), conceptual framework (reference in Figure 5) and geographic distribution map (reference in Figure 6) are referenced and linked.

Reviewer #3: The authors have done a good job integrating first-round comments regarding interpretability and causal inference. The cautioning around GWAS on the African continent prior to clinical or policy applications, and the emphasis on more reliable comparability before drawing inferences, are well handled.

DENV: The retention of pooled estimates in the main text is difficult to justify given the authors' own acknowledgement of extreme heterogeneity in case definitions, ascertainment methods, population immunity profiles, and circulating serotypes. If the authors themselves enumerate these sources of heterogeneity, it raises the question of what exactly is being compared. Beyond being interpreted or compared with “considerable caution”, these estimates in clinical severity profiles in African vs Asian cohorts cannot be meaningfully interpreted at all. The inclusion of the Cuban comparator compounds this issue: it represents a distinct population and is a genomic study being pooled with already heterogeneous observational and case studies. The sensitivity analysis, which yields a lower estimate, further illustrates that variability in results is itself an argument against presenting pooled ratios in the main text at all

RVFV: I recognise the methodological effort involved in deriving the pooled RVFV estimate, but the individual study estimates are more informative and should be the focus. Presenting a pooled figure that the authors themselves caution against interpreting does not serve the reader. It would be more transparent and scientifically defensible to explain explicitly why pooled estimates are not presented.

CHIKV: The introductory paragraph on CHIKV should explicitly state that findings from La Réunion cannot be extrapolated to continental Africa without significant caution, given disparities in health infrastructure and access to care. This framing is thereafter well contextualised through the results and discussion sections wherever La Réunion data appear.

ZIKV: To my knowledge, the African ZIKV lineage was not endemic in Cabo Verde (line 354); authors should specify where it was endemic: e.g. Senegal, which is a neighbouring country (https://doi.org/10.3201/eid2606.190928 ; https://doi.org/10.1371/journal.pntd.0002636). Faye et. al 2020 presents a plausible explanation for why the Asian lineage may have been observed in Cabo Verde rather than the African lineage endemic to Senegal.

**********

**Conclusions**

-Are the conclusions supported by the data presented?

-Are the limitations of analysis clearly described?

-Do the authors discuss how these data can be helpful to advance our understanding of the topic under study?

-Is public health relevance addressed?

Reviewer #1: The conclusions are supported by the presented data and are now more aligned with the strength and certainty of the available evidence than in the original submission. The authors substantially improved their interpretation of the findings by acknowledging the limitations associated with high between-study heterogeneity and varying evidence quality, thus avoiding overstatement of the results.

The limitations of the study are clearly described and discussed appropriately. The revised manuscript provides a balanced assessment of the methodological constraints, including heterogeneity among studies, variability in study quality, potential publication bias, and the limited availability of high-quality data for several arboviral diseases. These considerations are incorporated into the interpretation of the findings.

The discussion clearly explains how the results advance current knowledge of the epidemiology of major arboviral diseases in African populations. The manuscript identifies important knowledge gaps and highlights priorities for future epidemiological and genomic research. It also provides a comprehensive synthesis of the available evidence, which will be valuable for researchers and public health professionals.

The public health relevance of the study is well addressed. The authors appropriately discuss the implications of their findings for surveillance, research prioritization, and evidence-based public health planning, drawing conclusions that are proportional to the certainty of the available evidence.

Overall, I do not identify any additional analyses or experiments necessary before publication. The remaining observations are limited to minor editorial refinements.

Reviewer #2: Overall, the conclusions are well supported by the evidence provided and are expressed with commendable epistemic caution throughout. The three main conclusions are directly supported by the data presented in the Results: (1) convergent cytokine dysregulation in fatal RVFV and YFV; (2) preliminary, hypothesis-generating evidence for genetically mediated dengue protection linked to African ancestry; and (3) a high burden of chronic CHIKV arthropathy (with the appropriate caveat that evidence is predominantly from La Réunion).

The characterization of genetic protection against severe dengue as "preliminary and hypothesis-generating" is the proper epistemic stance in light of the available evidence: three independent lines of evidence (Boillat-Blanco et al., Sierra et al., García et al.) that are individually underpowered or methodologically constrained, but collectively suggestive. The Tassembedo et al. (2025) Burkina Faso data, which show allelic non-association for TNF-α −308 and IFN-γ +874, provide valuable continental African data but do introduce a certain level of contradiction with the protection hypothesis, which the authors report correctly ("allelic associations did not reach statistical significance") without over-interpretation, which is correct.

The Limitations section is well written and formally structured, and includes: predominance of cross-sectional and outbreak designs; low NOS scores (only 3 of 155 rated as low risk of bias); unavailability of individual patient data (preventing cross-study confounder adjustment); restriction of meta-analysis to two virus-outcome combinations; extreme heterogeneity; DL estimator limitations; Egger's test asymmetry for RVFV; La Réunion dependency for CHIKV; diagnostic method heterogeneity; and inclusion of Cuban data as comparator populations. This is a very extensive limitations discussion.

There is good coverage of public health relevance throughout. The burden of the five arboviruses in Africa is set in the Introduction. The Author Summary (clearly targeted at a lay/policymaker audience) communicates key findings accessibly, including the "1 in 12 dengue patients developing severe disease" figure for Africa versus the "1 in 4 or 5" comparator for Asian settings, with appropriate caveats about comparative interpretation. The ZIKV data gap is clearly stated as a public health issue. The framing of climate change in the Conclusions introduces a forward-looking public health perspective.

Reviewer #3: The authors repeatedly caution that results should be interpreted carefully, yet continue to present pooled severe dengue estimates, and attempt to make comparisons in African vs Asian cohorts. A cautionary sentence is insufficient here, as a strong basis for comparability is not established. I strongly recommend removing these comparisons and instead noting explicitly that this absence is due to the extent of heterogeneity. Further studies with more rigorous comparability are needed before such comparisons can be made ethically.

The discussion's integration of the social and political construction of race, social determinants of health, and gene-environment interactions is well done and appropriately contextualises observed differences in the reference studies. The justification for further studies measuring social determinants of health is well reasoned, however, I do not suggest mentioning “adjusting” for social determinants of health (line 445). Adjusting for social determinants would limit generalisability to real-world contexts, where these factors very well interact with health outcomes, including genetic factors.

Note: The low GRADE ratings should be explicitly acknowledged in the limitations section

**********

**Editorial and Data Presentation Modifications?**

Use this section for editorial suggestions as well as relatively minor modifications of existing data that would enhance clarity. If the only modifications needed are minor and/or editorial, you may wish to recommend “Minor Revision” or “Accept”.

Reviewer #1: Following revision, the manuscript has improved substantially, and only a few minor editorial and presentation issues remain.

1. A final editorial review of the reference list is recommended to ensure complete consistency with Vancouver style, including journal abbreviations, punctuation, capitalization, bibliographic completeness, and DOI formatting.

2. Please verify that all supplementary files are correctly cited, consistently numbered, and accurately cross-referenced throughout the manuscript.

3. A final proofread of the English text would improve readability by correcting minor stylistic inconsistencies and enhancing sentence flow.

4. During production, authors should ensure that figures and tables are legible in the final publication format. This includes font size, axis labels, legends, and image resolution.

These recommendations are editorial in nature and do not affect the scientific validity of the manuscript. I do not consider any additional analyses or experiments necessary before publication.

Accordingly, I recommend minor revision.

Reviewer #2: Date inconsistency (Minor — Correct): The PROSPERO registration date is given as "7th Marc 2026" which is a typographical error for "7th March 2026. The Methods section correctly mentions "7th March 2026. This should be corrected in the Abstract.

Introduction — Apostrophe error (Minor): Line in the Introduction reads: "Five arthropod-borne viruses' dengue (DENV), chikungunya (CHIKV)..." The possessive apostrophe after "viruses'" is incorrect; the sentence is intended to use a dash or em-dash separator. It should read: "Five arthropod-borne viruses (dengue (DENV), chikungunya (CHIKV), yellow fever (YFV), Rift Valley fever (RVFV), and Zika (ZIKV)) collectively make up..." This error is in the first paragraph of the Introduction and causes an immediate grammar error

Climate change conclusion — word choice (Minor): The word will in "will be relevant to understanding clinical outcomes in these newly exposed populations" should be changed to may be relevant to reflect the speculative nature of this extrapolation as noted in Section C.

Abbreviations — PECO not introduced in Abstract (Minor): PECO is used as a framework heading in the Methods but the acronym is not defined in the Abstract where readers first encounter the methodological framing. Either spell out "Population, Exposure, Comparison, Outcome" the first time it is used or do not use the acronym in that section.

Recommendation: Minor Revision — the manuscript is essentially correct; the changes listed above can be made without new experiments or significant reorganization.

Reviewer #3: Minor edits with typos:

line 30 “7th of [March]”

line 292 “... response to RVFV [,] a finding with….”

The Egger's plot is more interpretable than previously, as is the map. However, as noted throughout this review, I recommend against presenting pooled estimates in the forest plots. Authors may consider producing individual forest plots for each of the five arboviruses, which would effectively illustrate the heterogeneity within each disease and further support the case for narrative synthesis over pooled estimation.

**********

**Summary and General Comments**

Use this section to provide overall comments, discuss strengths/weaknesses of the study, novelty, significance, general execution and scholarship. You may also include additional comments for the author, including concerns about dual publication, research ethics, or publication ethics. If requesting major revision, please articulate the new experiments that are needed.

Reviewer #1: I appreciate the considerable effort the authors invested in revising this manuscript. After carefully evaluating the revised version, I conclude that it has improved substantially in response to the concerns I raised in my previous review.

The revised manuscript demonstrates significant advances in methodological transparency, statistical justification, reproducibility, and overall scientific presentation. Specifically, the authors strengthened the epidemiological background, clarified the study objectives and research question, expanded the description and justification of the statistical methods, improved the discussion of heterogeneity and limitations, enhanced the interpretation of the findings, and increased transparency by providing supplementary materials and analytical code.

The manuscript presents a comprehensive, well-executed systematic review and meta-analysis that addresses an important public health topic. The synthesis of available evidence across five major arboviral diseases in African populations is a valuable contribution to the field, providing useful information for researchers, epidemiologists, and public health professionals. The discussion appropriately identifies important knowledge gaps and outlines priorities for future epidemiological and genomic research.

I do not identify any major remaining methodological, statistical, ethical, or scientific concerns requiring additional analyses or experiments. The remaining observations are limited to minor editorial and presentation refinements, including final verification of the reference list, cross-referencing of the supplementary material, language editing, and improving figure readability.

Overall, I consider the principal concerns raised in my previous review to have been satisfactorily addressed. In my opinion, the manuscript is ready for publication once the minor editorial issues have been addressed.

**Recommendation: Minor Revision.**

Reviewer #2: Overall Assessment:

This is a well-executed and timely systematic review with meta-analysis focusing on a real evidence gap, synthesis of viral and host determinants of arboviral disease severity specifically in African and African-ancestry populations in particular. The size of the review (five arboviruses, 155 studies, 38 countries, 48 years of publication) is impressive, and the authors have expended a significant amount of effort to ensure the methodological rigour, GRADE assessment, and transparent reporting.

Strengths:

There are certain good features in the manuscript. The research question is clearly expressed and the PECO framework is clearly presented (a methodological detail that is not widely used in the infectious disease literature). Pre-registration to PROSPERO is confirmed and adherence to PRISMA 2020 is confirmed. There is excellent inter-rater agreement at the screening stage (κ = 0.82 and 0.91). Technically, the Freeman–Tukey transformation decision is warranted. Appropriate use of GRADE and an appropriate certainty level of Very Low is used and reported correctly. The analytical code transparency (as Python scripts in Supplementary File S1) is exemplary, and full reproducibility is observed, as is not the case for many systematic reviews in the field. I would also note that the GRADE summary-of-findings tables (supplementary Table S5), the detailed, 39-field data extraction form (supplementary Table S3), and the full search strategy (supplementary Table S4) are unusually rigorous in the reporting of methodological details.

The Discussion is well informed on scientific matters, and is substantive, attending the mechanistic implications of the main findings whilst being epistemically cautious. Using the language of race as a social construct, with due notice to the careful epistemological nuance taken in the use of the Boillat-Blanco et al. data, evidences a scholarly maturity and is just what the field needs. This is correct and significant: The identification of a paucity of clinical data from Africa as ‘the single most conspicuous evidence gap'.

Weaknesses:

In spite of editing, there are a few flaws:

While the DL estimator is known to be problematic when there is high heterogeneity, it is still used for all pooled estimates. They may be artificially narrowed, especially for DENV (I² = 95.7%). A REML or Paule–Mandel sensitivity analysis is a way of solving this without having to overhaul the method completely.

The word 'will' in the Conclusions is still inferentially reaching too far the climate change extrapolation. It's a small word difference in the conclusion that makes a huge impression to those who are policy makers.

PROSPERO number format is not standard and needs to be checked.

Novelty and Significance:

The novelty of this review is obvious: a systematic review has not previously synthesised across all 5 major arboviruses in a comparative framework for Africa to date. The convergent cytokine signature of fatal RVFV and YFV (IL-6, MCP-1, IP-10) suggests a common terminal inflammatory pathway, which has real therapeutic implications for host-directed therapy in the context of resource-limited countries where aetiological diagnosis is often not available.

There are no dual publication concerns identified. Study is a systematic review of published literature and primary sources of literature are handled appropriately.

General Recommendation: Minor revision.

Reviewer #3: Overall the authors present a much stronger paper with integrated suggestions. The discussion portion especially is much more robust. While the authors have acknowledged heterogeneity in study design, clinical diversity, and methodological variation across included papers, they have nonetheless retained pooled estimates in the main text. The volume of cautionary language required to contextualise these estimates, in my opinion, undermines the case for presenting them at all. I recommend moving pooled estimates to supplementary materials, with a note in the main text directing interested readers there, and restructuring the main results around individual study findings and narrative synthesis, explaining why estimates were not pooled. This is further supported by the low GRADE ratings, which should guide decisions about what is foregrounded in the main paper.

**********

PLOS authors have the option to publish the peer review history of their article (what does this mean?). If published, this will include your full peer review and any attached files.

If you choose “no”, your identity will remain anonymous but your review may still be made public.

**Do you want your identity to be public for this peer review?**  For information about this choice, including consent withdrawal, please see our Privacy Policy.

Reviewer #1: No

Reviewer #2: **Yes:**  Nnamso Ikpeida

Reviewer #3: No

**Figure resubmission:**

**Reproducibility:**

To enhance the reproducibility of your results, we recommend that authors of applicable studies deposit laboratory protocols in protocols.io, where a protocol can be assigned its own identifier (DOI) such that it can be cited independently in the future. Additionally, PLOS ONE offers an option to publish peer-reviewed clinical study protocols. Read more information on sharing protocols at https://plos.org/protocols?utm_medium=editorial-email&utm_source=authorletters&utm_campaign=protocols

Attachment Submitted filename: renamed_1df65.pdf

# Author comment: Author response to Decision Letter 2

**DOI:** 10.1371/journal.pntd.0014663.r004

22 Jul 2026

Attachment Submitted filename: PNTD_Response_to_Reviewers_FINAL.docx

# Editor report: Decision Letter 2

**DOI:** 10.1371/journal.pntd.0014663.r005

**Authors:** Sujatha Sunil

10 Aug 2026

Dear DR Asaga,

We are pleased to inform you that your manuscript 'Viral and host determinants of arboviral disease severity in African populations: a systematic review with meta-analysis' has been provisionally accepted for publication in PLOS Neglected Tropical Diseases.

Before your manuscript can be formally accepted you will need to complete some formatting changes, which you will receive in a follow up email. A member of our team will be in touch with a set of requests.

Please note that your manuscript will not be scheduled for publication until you have made the required changes, so a swift response is appreciated.

IMPORTANT: The editorial review process is now complete. PLOS will only permit corrections to spelling, formatting or significant scientific errors from this point onwards. Requests for major changes, or any which affect the scientific understanding of your work, will cause delays to the publication date of your manuscript.

Should you, your institution's press office or the journal office choose to press release your paper, you will automatically be opted out of early publication. We ask that you notify us now if you or your institution is planning to press release the article. All press must be co-ordinated with PLOS.

Thank you again for supporting Open Access publishing; we are looking forward to publishing your work in PLOS Neglected Tropical Diseases.

Best regards,

Sujatha Sunil, PhD

Section Editor

PLOS Neglected Tropical Diseases

Sujatha Sunil

Section Editor

PLOS Neglected Tropical Diseases

Shaden Kamhawi

co-Editor-in-Chief

PLOS Neglected Tropical Diseases

orcid.org/0000-0003-4304-636XX

Paul Brindley

co-Editor-in-Chief

PLOS Neglected Tropical Diseases

orcid.org/0000-0003-1765-0002

***********************************************************

# Editor report: Acceptance letter

**DOI:** 10.1371/journal.pntd.0014663.r006

**Authors:** Sujatha Sunil

Dear DR Asaga,

We are delighted to inform you that your manuscript, "Viral and host determinants of arboviral disease severity in African populations: a systematic review with meta-analysis," has been formally accepted for publication in PLOS Neglected Tropical Diseases.

We have now passed your article onto the PLOS Production Department who will complete the rest of the publication process. All authors will receive a confirmation email upon publication.

The corresponding author will soon be receiving a typeset proof for review, to ensure errors have not been introduced during production. Please review the PDF proof of your manuscript carefully, as this is the last chance to correct any scientific or type-setting errors. Please note that major changes, or those which affect the scientific understanding of the work, will likely cause delays to the publication date of your manuscript. Note: Proofs for Front Matter articles (Editorial, Viewpoint, Symposium, Review, etc...) are generated on a different schedule and may not be made available as quickly.

Soon after your final files are uploaded, the early version of your manuscript will be published online unless you opted out of this process. The date of the early version will be your article's publication date. The final article will be published to the same URL, and all versions of the paper will be accessible to readers.

For Research Articles, you will receive an invoice from PLOS for your publication fee after your manuscript has reached the completed accept phase. If you receive an email requesting payment before acceptance or for any other service, this may be a phishing scheme. Learn how to identify phishing emails and protect your accounts at https://explore.plos.org/phishing.

Thank you again for supporting open-access publishing; we are looking forward to publishing your work in PLOS Neglected Tropical Diseases.

Best regards,

Shaden Kamhawi

co-Editor-in-Chief

PLOS Neglected Tropical Diseases

Paul Brindley

co-Editor-in-Chief

PLOS Neglected Tropical Diseases
